# Modulated Calcium Homeostasis and Release Events Under Atrial Fibrillation and Its Risk Factors: A Meta-Analysis

**DOI:** 10.3389/fcvm.2021.662914

**Published:** 2021-07-20

**Authors:** Sarah Pei Ting Fong, Shaleka Agrawal, Mengqi Gong, Jichao Zhao

**Affiliations:** ^1^Auckland Bioengineering Institute, The University of Auckland, Auckland, New Zealand; ^2^Department of Cardiology, The First Affiliated Hospital of Anhui Medical University, Hefei, China

**Keywords:** atrial fibrillation, calcium handling, calcium release events, Ca^2+^ sparks, primary AF prevention, secondary AF prevention

## Abstract

**Background:** Atrial fibrillation (AF) is associated with calcium (Ca^2+^) handling remodeling and increased spontaneous calcium release events (SCaEs). Nevertheless, its exact mechanism remains unclear, resulting in suboptimal primary and secondary preventative strategies.

**Methods:** We searched the PubMed database for studies that investigated the relationship between SCaEs and AF and/or its risk factors. Meta-analysis was used to examine the Ca^2+^ mechanisms involved in the primary and secondary AF preventative groups.

**Results:** We included a total of 74 studies, out of the identified 446 publications from inception (1982) until March 31, 2020. Forty-five were primary and 29 were secondary prevention studies for AF. The main Ca^2+^ release events, calcium transient (standardized mean difference (SMD) = 0.49; *I*^2^ = 35%; confidence interval (CI) = 0.33–0.66; *p* < 0.0001), and spark amplitude (SMD = 0.48; *I*^2^ = 0%; CI = −0.98–1.93; *p* = 0.054) were enhanced in the primary diseased group, while calcium transient frequency was increased in the secondary group. Calcium spark frequency was elevated in both the primary diseased and secondary AF groups. One of the key cardiac currents, the L-type calcium current (I_CaL_) was significantly downregulated in primary diseased (SMD = −1.07; *I*^2^ = 88%; CI = −1.94 to −0.20; *p* < 0.0001) and secondary AF groups (SMD = −1.28; *I*^2^ = 91%; CI = −2.04 to −0.52; *p* < 0.0001). Furthermore, the sodium–calcium exchanger (I_NCX_) and NCX1 protein expression were significantly enhanced in the primary diseased group, while only NCX1 protein expression was shown to increase in the secondary AF studies. The phosphorylation of the ryanodine receptor at S2808 (pRyR-S2808) was significantly elevated in both the primary and secondary groups. It was increased in the primary diseased and proarrhythmic subgroups (SMD = 0.95; *I*^2^ = 64%; CI = 0.12–1.79; *p* = 0.074) and secondary AF group (SMD = 0.66; *I*^2^ = 63%; CI = 0.01–1.31; *p* < 0.0001). Sarco/endoplasmic reticulum Ca^2+^-ATPase (SERCA) expression was elevated in the primary diseased and proarrhythmic drug subgroups but substantially reduced in the secondary paroxysmal AF subgroup.

**Conclusions:** Our study identified that I_CaL_ is reduced in both the primary and secondary diseased groups. Furthermore, pRyR-S2808 and NCX1 protein expression are enhanced. The remodeling leads to elevated Ca^2+^ functional activities, such as increased frequencies or amplitude of Ca^2+^ spark and Ca^2+^ transient. The main difference identified between the primary and secondary diseased groups is SERCA expression, which is elevated in the primary diseased group and substantially reduced in the secondary paroxysmal AF subgroup. We believe our study will add new evidence to AF mechanisms and treatment targets.

## Introduction

Atrial fibrillation (AF) is the most common sustained arrhythmia, with markedly increasing prevalence (1, 2). It is associated with significant mortality and morbidity and becomes more challenging to treat as it advances (3–5). AF is mainly managed by primary and/or secondary preventative therapies. Primary prevention includes early detection and intervention on risk factors before AF develops, while secondary prevention involves diagnosing and treating AF (4). However, current pharmacological strategies are often associated with limited efficacy and adverse consequences, mainly due to an incomplete understanding of underlying cellular mechanisms related to AF (4, 6). In particular, calcium (Ca^2+^) is one of the most crucial ions for cardiac excitation–contraction coupling and Ca^2+^-dependent signaling pathways for maintaining cardiac function (7, 8).

Intracellular Ca^2+^ release events are exclusively investigated in myocardial physiology and pathophysiology, as they hold the key to understanding how cardiomyocyte Ca^2+^ signaling is regulated by ionic channels and Ca^2+^ proteins (7, 8). In a single cardiac cycle, the L-type calcium channels (LTCCs) localized on the sarcolemma and tubules are first activated (9). The opening of LTCCs results in the movement of Ca^2+^ into the cytosol, which induces the cardiac type 2 ryanodine receptors (RyR2) located on the junctional sarcoplasmic reticulum (SR) to release Ca^2+^ from its stores into the cytosol (9–11). This elementary Ca^2+^ release event is observed as a form of a Ca^2+^ spark, and the process is known as calcium-induced calcium release (12). Increases in highly localized, short-lived Ca^2+^ signals raise intracellular Ca^2+^ [Ca^2+^]_i_, which contributes to global Ca^2+^ waves or transients that propagate through the cell (10, 13). [Ca^2+^]_i_ then binds to troponin to allow myosin adenosine triphosphatase (ATPase) to bind to actin in the sarcomere to initiate cardiac contraction (9, 14). Ca^2+^ is mainly recycled back into the SR *via* the SR Ca^2+^-ATPase (SERCA2a) pump or extruded across the cell membrane through the cardiac sodium–calcium exchanger (NCX1) (15). The reduction in [Ca^2+^]_i_ causes Ca^2+^ to dissociate from troponin and terminate myofilament cross-bridge cycling for cardiac relaxation (9, 16). SERCA2a activity is directly modulated by phospholamban (PLN). In its unphosphorylated state, PLN acts as an inhibitor to SERCA2a. When phosphorylated by protein kinase A (PKA), PLN dislodges from SERCA2a to enable the reuptake of Ca^2+^ (9). Another important signaling protein besides PKA is the Ca^2+^/calmodulin-dependent protein kinase II (CAMKII), which is responsible for transducing cytosolic Ca^2+^, and calmodulin, a Ca^2+^-binding messenger protein that modulates RyR activity and transduces Ca^2+^ signals to other protein kinases or phosphatases (17–19).

In diseased states, spontaneous Ca^2+^ release events (SCaEs) are observed as spontaneous Ca^2+^ sparks or arrhythmogenic Ca^2+^ waves are substantially enhanced (20, 21). Such defective Ca^2+^ homeostasis often results from remodeled Ca^2+^-handling proteins (22–24). However, current studies reported conflicting results on how these Ca^2+^-handling proteins were remodeled in AF and its risk factors, which hinders the development of effective AF treatment and prevention. In this study, we aim to illustrate the precise mechanisms and targeted therapies for AF and AF prevention by investigating the pathophysiological role of Ca^2+^ and its arrhythmogenicity. This systematic review has compared the different Ca^2+^ mechanisms between the primary and secondary AF preventative groups in the existing studies to date.

## Methods

This systematic review was conducted according to the Preferred Reporting Items for Systematic Reviews and Meta-Analysis (PRISMA) guidelines (refer to the PRISMA 2009 checklist in Supplementary Table 1) (25).

### Search Strategy and Eligibility

The systematic electronic search was performed using the terms “atrial fibrillation” AND “calcium wave”/“calcium transient”/“calcium spark” in all fields to identify articles in PubMed from inception through March 31, 2020. Based on their titles and abstracts, the searched articles were screened manually for inclusion. Screening criteria included publications that mapped SCaEs in atrial cardiomyocytes in sinus rhythm and/or AF. Publications that did not conduct any experimental studies on atrial cells, such as mathematical modeling, population-based or organ-level studies, review papers, and editorial reports, were excluded. We also excluded papers that focused on genes and/or miRNA, signaling pathways, tissue, and organelle calcium experimental studies. Two co-authors then reviewed the screened articles in full text for eligibility, and those that met the criteria were selected. Any discrepancies were resolved by a third author through discussion and consensus. Full details of the search terms were presented in Supplementary Table 1. The quality of the included studies was assessed according to the Newcastle–Ottawa Scale (Supplementary Table 2) (26). A study with a score of 5 and above was considered satisfactory.

### Data Extraction

The atrial cellular activities were extracted from selected studies. It included SCaEs [Ca^2+^ spark frequency (CaSpF), Ca^2+^ transient frequency (CaTF), Ca^2+^ spark amplitude (CaSpA), and Ca^2+^ transient amplitude (CaTA)], and Ca^2+^ load and leak. It also included atrial current densities, such as L-type calcium current (I_CaL_), sodium–calcium exchanger current (I_NCX_), late sodium current (I_Na−Late_), and potassium current (I_K_), and protein expressions, such as L-type alpha 1C subunit voltage-dependent calcium current (Ca_v_1.2), NCX1, RyR2, phosphorylated ryanodine receptor 2 (pRyR2), SERCA2a, PLN, phosphorylated phospholamban (pPLN), CAMKII, phosphorylated Ca^2+^/calmodulin-dependent protein kinase II (pCAMKII), and PKA. We further categorized the above results into two main groups: primary and secondary preventative therapies for AF. Primary prevention was divided into three subclasses: the diseased group (risk factors for AF), the application of proarrhythmogenic agents or antiarrhythmogenic agents. Secondary prevention was classified into either the paroxysmal or chronic AF group. The analysis was conducted using *R*.

### Data Synthesis and Statistical Analysis

Statistical analyses were performed using *R* (27). Dichotomous values were used to calculate 95% confidence interval (CI) of relative risk ratios, and continuous values to quantify standardized mean difference (SMD). Each study was given a weighting factor to determine its importance in the meta-analysis, which was represented by gray boxes in forest plots. When the boundaries of the CI were within the box, a white horizontal line was plotted; otherwise, it was illustrated by a black horizontal line. Studies with the CI not crossing zero were deemed to be statistically significant.

The overall SMD was interpreted using Cohen's guidelines (28), where a value of 0.2–0.49 was deemed to be small, 0.5–0.79 represented medium, and 0.8 and above was large. Statistical heterogeneity was calculated using *I*^2^ for all studies (29). In general, heterogeneity was classified into three main categories, low, medium, and high, when *I*^2^ values were ≤ 25%, between 25 and 50%, and ≥75%, respectively. Statistical significance was measured with *p*-values. We considered a result to be statistically significant when *p*-value was ≤ 0.05. We also employed influence analysis and graphic display of heterogeneity (GOSH) plots to detect influential studies and remove outliers (27).

## Results

### Study Characteristics

**Figure 1:**
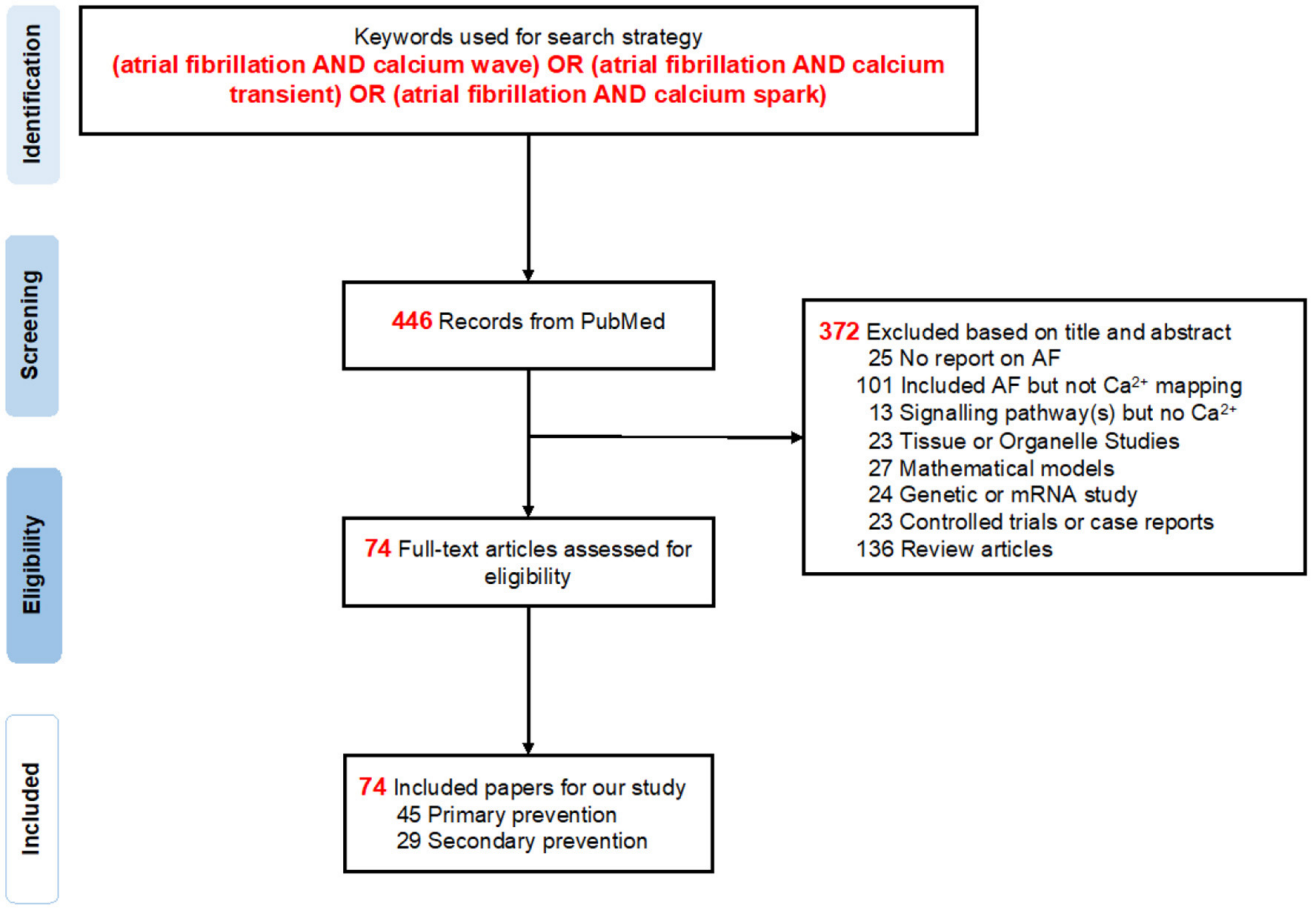
PRISMA flow diagram. A total of 446 records were found in PubMed, from which 74 studies were selected for further analysis in this systematic review.

Our literature search identified a total of 446 publications from inception (1982) to March 31, 2020 (Figure 1). When screening the titles and abstracts, a total of 372 papers were excluded (Supplementary Table 3): 25 articles focused on other diseases instead of AF, 101 included AF but did not conduct experiments on SCaEs, 13 papers mentioned signaling pathways but not Ca^2+^, 23 were tissue or organelle studies, another 24 studied mRNA and genes, 27 indicated mathematical models, 23 articles were controlled trials or case reports, and 136 were review articles. Eventually, a total of 74 studies (12, 24, 30–100), consisting of 45 primary and 29 secondary prevention studies for AF, were eligible and included for this systematic review.

**Figure 2:**
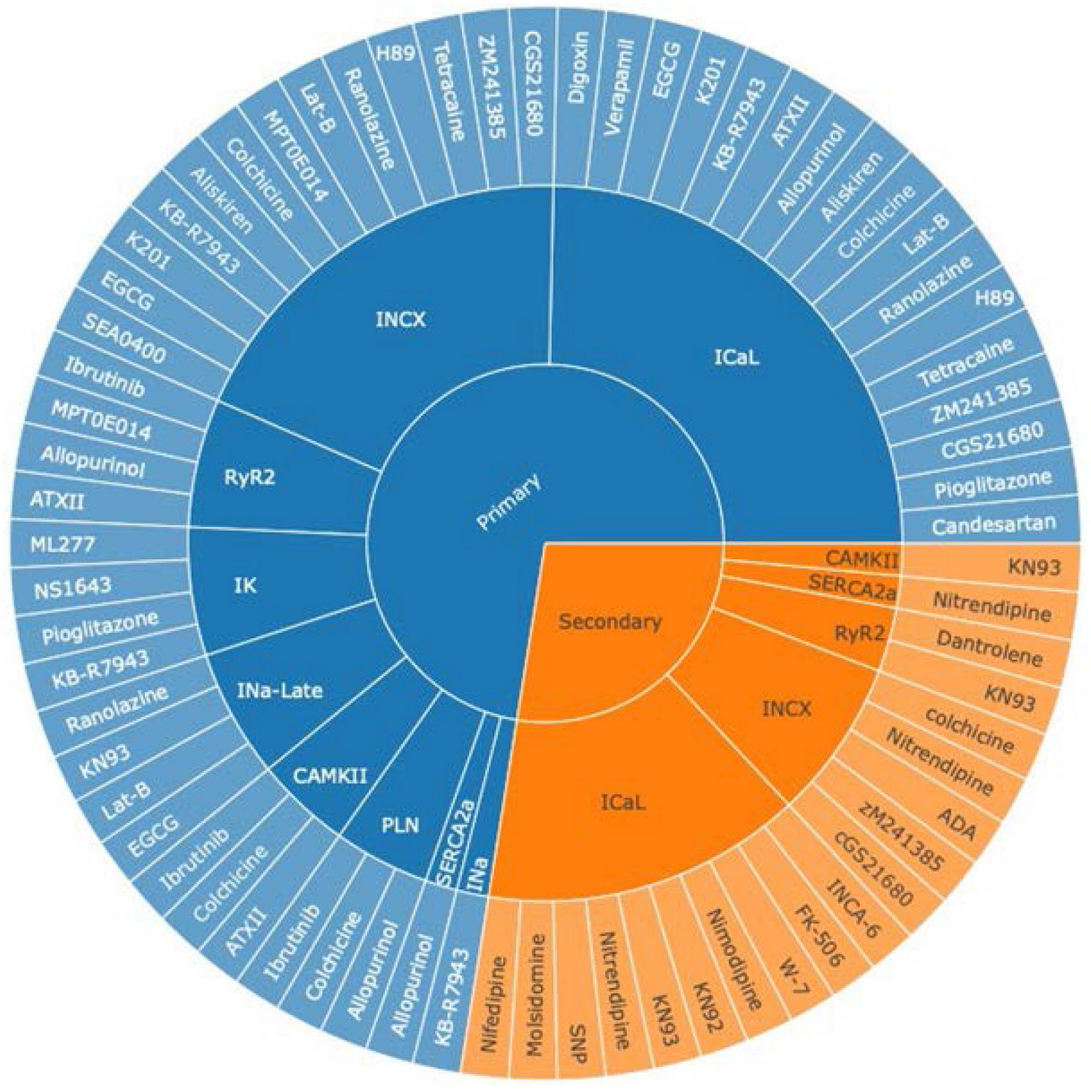
An overview of pharmacologically targeted ionic channels or proteins for AF treatment, grouped by primary (blue) and secondary (orange) prevention. The sunburst plot's core was divided into two groups, primary and secondary prevention for AF, where the middle section represented ion channels, and the outer segment symbolized various drug therapies. I_NCX_ stands for the sodium–calcium exchanger current; I_CaL_, the L-type calcium current; RyR2, the ryanodine receptor 2; I_K_, the potassium current; I_Na−Late_, the late sodium current; CAMKII, the Ca^2+^/calmodulin-dependent protein kinase II; PLN, phospholamban; SERCA2a, the sarco/endoplasmic reticulum Ca^2+^-ATPase 2a pump; I_Na_, the sodium current.

Based on the 74 selected studies, pharmacological targets were grouped by their mechanism of action on the ionic channel(s) or protein(s). We discovered that I_CaL_ was the most widely studied current in both primary and secondary pharmacological therapy for AF, followed by I_NCX_ and RyR2 channels (Figure 2). This coincides with the present targeted drug therapies available for AF, where LTCC antagonists are one of the most frequently prescribed drugs for the treatment of hypertension and AF. Figure 2 aids us in understanding and exploring other potential pathways for therapeutic drug discovery, such as the I_NCX_ and RyR2. It is noteworthy that the late sodium current was only commonly studied for primary prevention.

### Spontaneous Calcium Release Events

**Figure 3:**
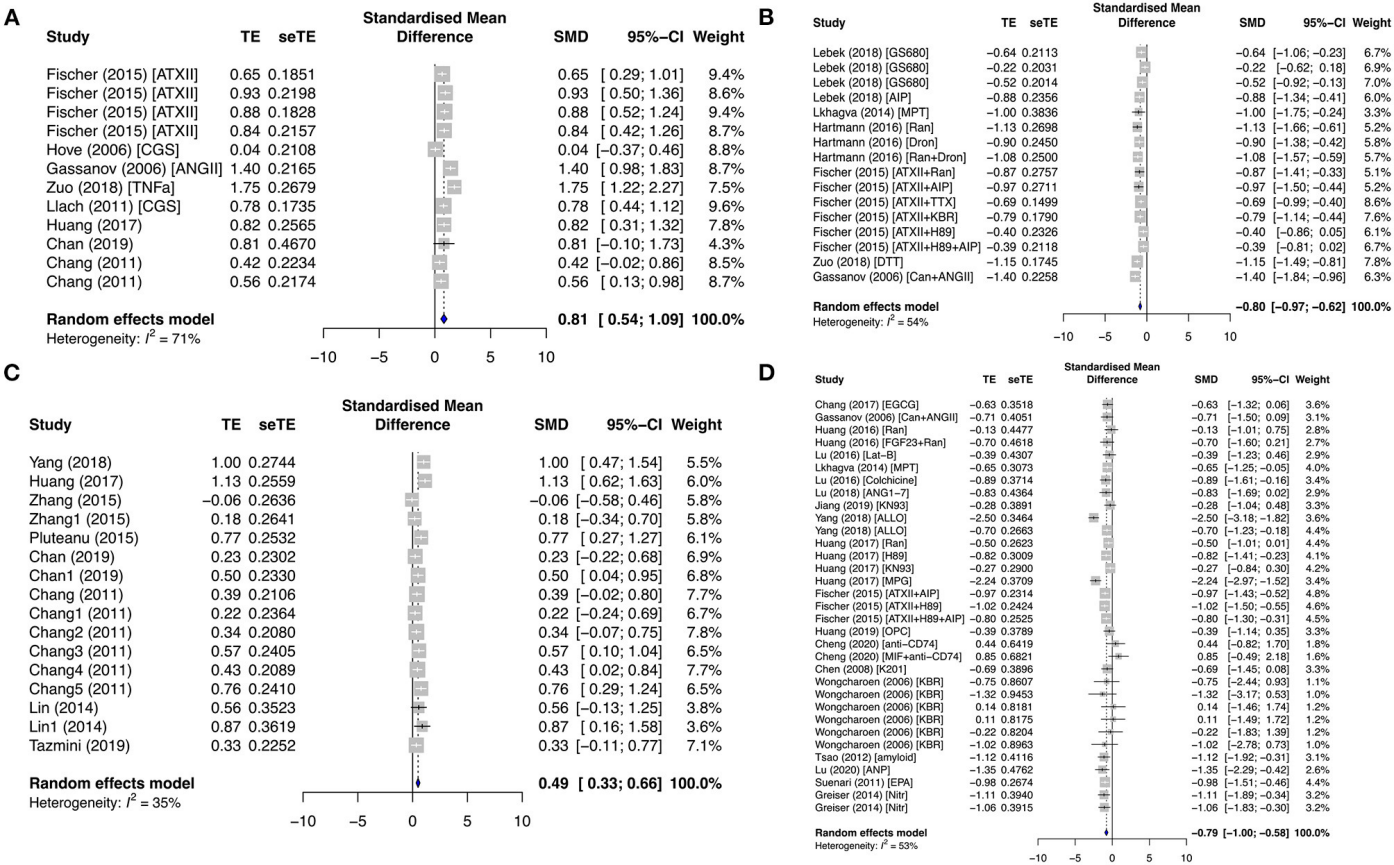
Calcium-release profiles in the primary prevention group. Calcium spark frequency (CaSpF) was represented in the **(A)** diseased and proarrhythmic subgroups and the **(B)** antiarrhythmic subgroup. Calcium transient amplitude (CaTA) was presented in the **(C)** diseased and **(D)** antiarrhythmic subgroups. TE, estimated treatment effect; seTE, standard error of treatment estimate; SMD, standard mean difference; 95% CI, 95% confidence interval; ATXII, anemonia viridis toxin 2; ANGII, angiotensin II; CGS21680, adenosine 2A agonist; TNFα, tumor necrosis factor-alpha; Ran, ranolazine; Dan, dantrolene; KN92, an inactive analog of KN93; KN93, calcium/calmodulin-dependent protein kinase II inhibitor; FGF23, fibroblast growth factor 23; Aldo, aldosterone; EGCG, epigallocatechin gallate; Can, candesartan; MPT0E014, histone deacetylase inhibitor; ANG1–7, angiotensin 1–7; ALLO, allopurinol; H89, protein kinase inhibitor; MPG, N-[2-mercaptopropionyl]glycine; OPC21286, arginine vasopressin antagonists; AIP, autocamide-2-related inhibitory peptide; MIF, macrophage inhibitory factor; K201, 1,4-benzothiazepine derivative; KB-R7943, reverse-mode sodium/calcium exchanger inhibitor; ANP, atrial natriuretic peptide; EPA, eicosapentaenoic acid; Nitr, nitrendipine.

The evolution of cardiac Ca^2+^ waves is influenced by local elevations of [Ca^2+^]_i_, seen as Ca^2+^ sparks. The properties (frequency and amplitude) of these Ca^2+^ release events are key determinants to the arrhythmogenicity of the cardiomyocytes. In our study, key calcium-handling remodeling including ionic currents, calcium release events, and protein expressions was summarized for the primary prevention and secondary AF groups in Figure 3. In the primary prevention group, CaSpF was significantly enhanced in the diseased subgroup (SMD = 0.6; *I*^2^ = 0%; CI = 0.30–0.89; *p* = 0.6601) and proarrhythmic drug subgroup (SMD = 0.89; *I*^2^ = 79%; CI = 0.48–1.30; *p* < 0.0001) (Supplementary Figures 1A,B). When these results were combined from both subgroups, they displayed a similar result (SMD = 0.81; *I*^2^ = 71%; CI = 0.54–1.09; *p* < 0.0001) (Figure 3A). The addition of antiarrhythmic drugs significantly decreased CaSpF (SMD = −0.80; *I*^2^ = 54%; CI = −0.97 to −0.62; *p* = 0.0054) (Figure 3B). A similar trend was observed for CaTA. CaTA was increased in the diseased subgroup (SMD = 0.49; *I*^2^ = 35%; CI = 0.33–0.66; *p* < 0.0001) (Figure 3C) and reduced by antiarrhythmic drugs (SMD = −0.79; *I*^2^ = 53%; CI = −1.00 to −0.58; *p* = 0.0002) (Figure 3D). In addition, CaSpA was enhanced in the diseased subgroup (Supplementary Figure 4).

**Figure 4:**
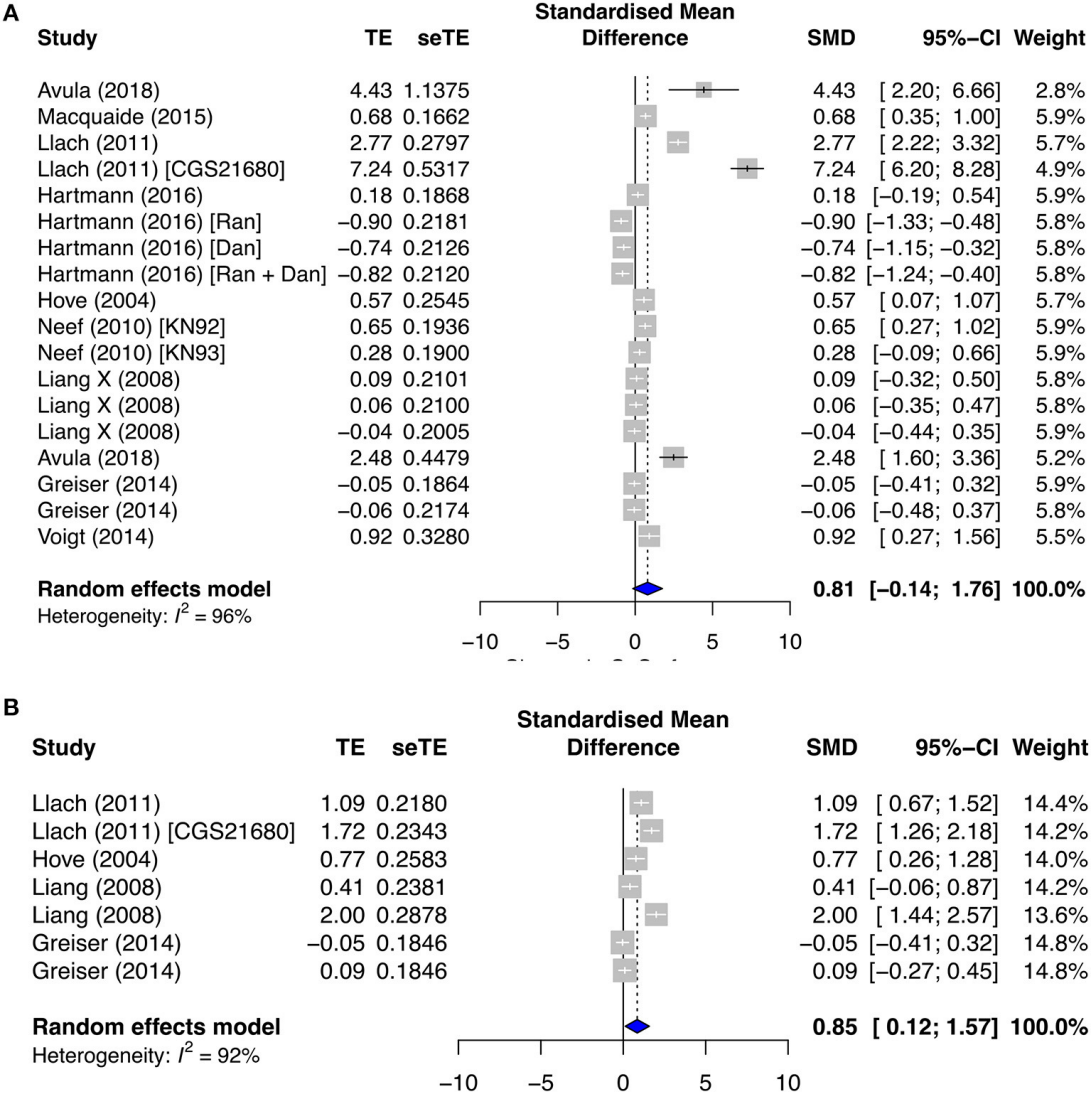
Calcium release profiles in the secondary prevention group. **(A)** Calcium spark frequency (CaSpF) and **(B)** calcium transient frequency (CaTF). TE, estimated treatment effect; seTE, standard error of treatment estimate; SMD, standard mean difference; 95% CI, 95% confidence interval; CGS21680, adenosine 2A agonist; Ran, ranolazine; Dan, dantrolene; KN92, an inactive analog of KN93; and KN93, calcium/calmodulin-dependent protein kinase II inhibitor.

In contrast, the CaSpF and calcium transient frequency (CaTF) were significantly elevated in both the secondary paroxysmal and chronic AF subgroups, with respective SMD = 0.81; *I*^2^ = 96%; CI = −0.14–1.76; *p* < 0.0001 (Figure 4A), and SMD = 0.85; *I*^2^ = 92%; CI = 0.12–1.57; *p* < 0.0001 (Figure 4B), and high heterogeneities. However, the change in CaSpA was almost negligible in both subgroups (SMD = 0.06; *I*^2^ = 55%; CI = 0.27–0.39; *p* < 0.0386) (Supplementary Figure 4G). Surprisingly, CaTA was unaltered in both paroxysmal (SMD = −0.07; *I*^2^ = 66%; CI = −0.34–0.20; *p* < 0.0001) and permanent AF (SMD = −0.06; *I*^2^ = 79%; CI = −0.49–0.38; *p* < 0.0001) (Supplementary Figures 2C,D).

### SR Ca^2+^ Leak–Load Relationship

**Figure 5:**
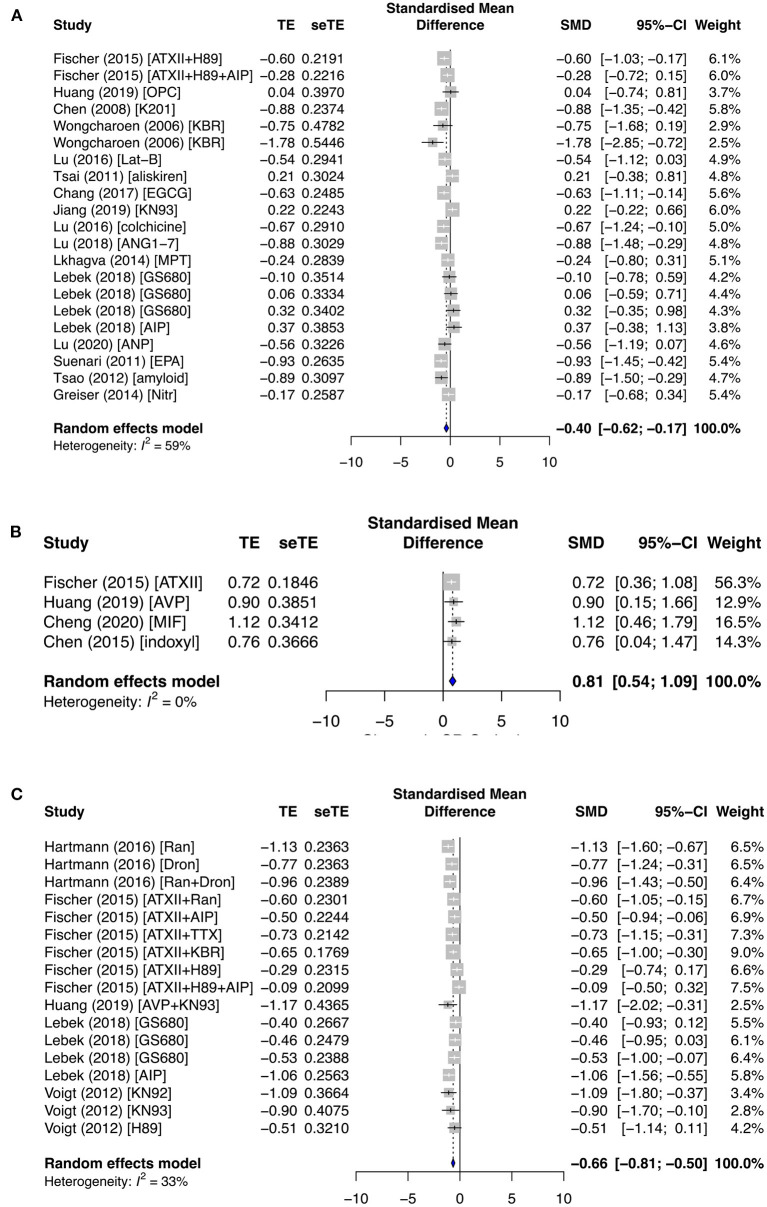
The effect of primary groups on SR calcium leak–load relationship. SR calcium load was affected by **(A)** antiarrhythmic drugs, and SR calcium leak was influenced by **(B)** proarrhythmic and **(C)** antiarrhythmic agents. TE, estimated treatment effect; seTE, standard error of treatment estimate; SMD, standard mean difference; 95% CI, 95% confidence interval; ATXII, anemonia viridis toxin 2; H89, protein kinase inhibitor; AIP, autocamide-2-related inhibitory peptide; OPC21286, arginine vasopressin antagonists; MIF, macrophage inhibitory factor; K201, 1,4-benzothiazepine derivative; KB-R7943, reverse-mode sodium/calcium exchanger inhibitor; Lat-B, latrunculin-B; EGCG, epigallocatechin gallate; ANG1–7, angiotensin 1–7; MPT0E014, histone deacetylase inhibitor; GS680, calcium/calmodulin-dependent protein kinase II inhibitor; EPA, eicosapentaenoic acid; Nitr, nitrendipine; AVP, arginine vasopressin; Ran, ranolazine; Dan, dantrolene; and TTX implies tetrodotoxin.

SR Ca^2+^ release is affected by the opening of RyR channels from its stores. In particular, SR Ca^2+^ leak is a major contributor to cardiac arrhythmia. Ca^2+^ load remained relatively unchanged in both the primary and secondary subgroups, except when antiarrhythmic drugs were applied in the primary group (SMD = −0.40; *I*^2^ = 59%; CI = −0.62 to −0.17; *p* < 0.0001) (Figure 5A). No change in Ca^2+^ leak was observed in the secondary prevention group (Supplementary Figure 5), but it was significantly affected by pro- and antiarrhythmic drugs in the primary subgroups. Ca^2+^ leak was raised by proarrhythmic agents (SMD = 0.81; *I*^2^ = 0%; CI = 0.54–1.09; *p* = 0.7583) (Figure 5B) and antagonized by antiarrhythmic agents (SMD = −0.66; *I*^2^ = 33%; CI = −0.81 to −0.50; *p* = 0.0932) (Figure 5C).

### Ionic Mechanisms of Atrial Remodeling

**Figure 6:**
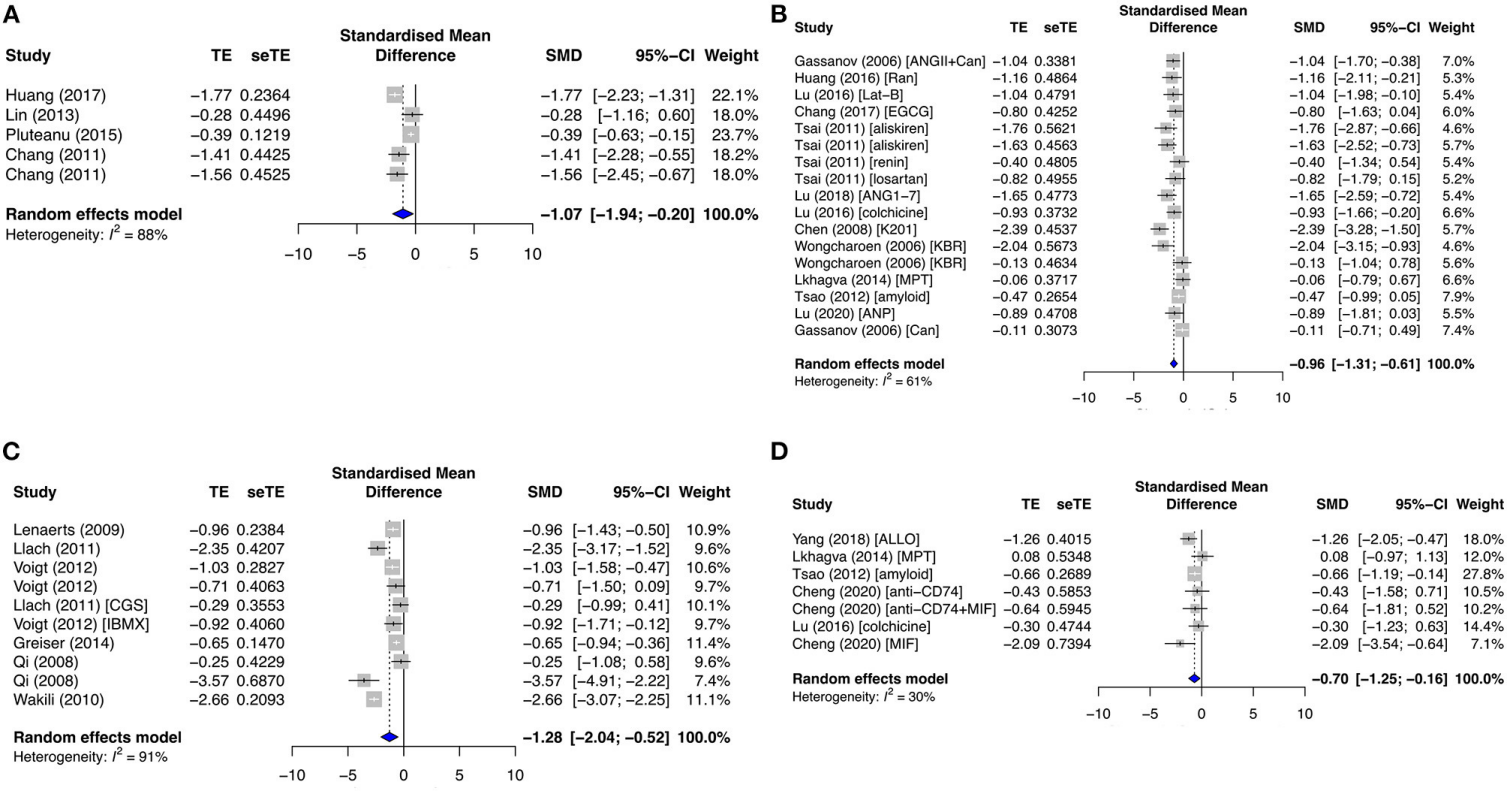
Measured L-type calcium current density (I_CaL_) and its protein expression (Ca_v_1.2) in the primary prevention group. **(A)** Diseased, **(B)** antiarrhythmic drug, and **(C)** secondary prevention group with AF studies, and **(D)** Ca_v_1.2 expression in the primary antiarrhythmic subgroup. TE, estimated treatment effect; seTE, standard error of treatment estimate; SMD, standard mean difference; 95% CI, 95% confidence interval; ANGII, angiotensin II; Can, candesartan; Ran, ranolazine; Lat-B, latrunculin-B; EGCG, epigallocatechin gallate; ANG1–7, angiotensin 1–7; K201, 1,4-benzothiazepine derivative; KB-R7943, reverse-mode sodium/calcium exchanger inhibitor; MPT0E014, histone deacetylase inhibitor; ANP, atrial natriuretic peptide; CGS21680, adenosine 2A agonist; IBMX, 3-isobutyl-1-methylxanthine; ALLO, allopurinol; MIF, macrophage inhibitory factor.

One of the most important currents for atrial cardiac action potential generation is I_CaL._ I_CaL_ was significantly downregulated in the primary diseased subgroup (SMD = −1.07; *I*^2^ = 88%; CI = −1.94 to −0.20; *p* < 0.0001) (Figure 6A), antiarrhythmic drug subgroup (SMD = −0.96; *I*^2^ = 61%; CI = −1.31 to −0.61; *p* < 0.0001) (Figure 6B), and secondary AF subgroups (SMD = −1.28; *I*^2^ = 91%; CI = −2.04 to −0.52; *p* < 0.0001) (Figure 6C). These results were consistent with Ca_v_1.2 protein expression in the primary antiarrhythmic subgroup (SMD = −0.70; *I*^2^ = 30%; CI = −1.25 to −0.16; *p* = 0.2027) (Figure 6D) and secondary permanent AF group (SMD = −1.69; *I*^2^ = 0%; CI = −7.05–3.67; *p* < 0.0001) (Supplementary Figure 6F).

**Figure 7:**
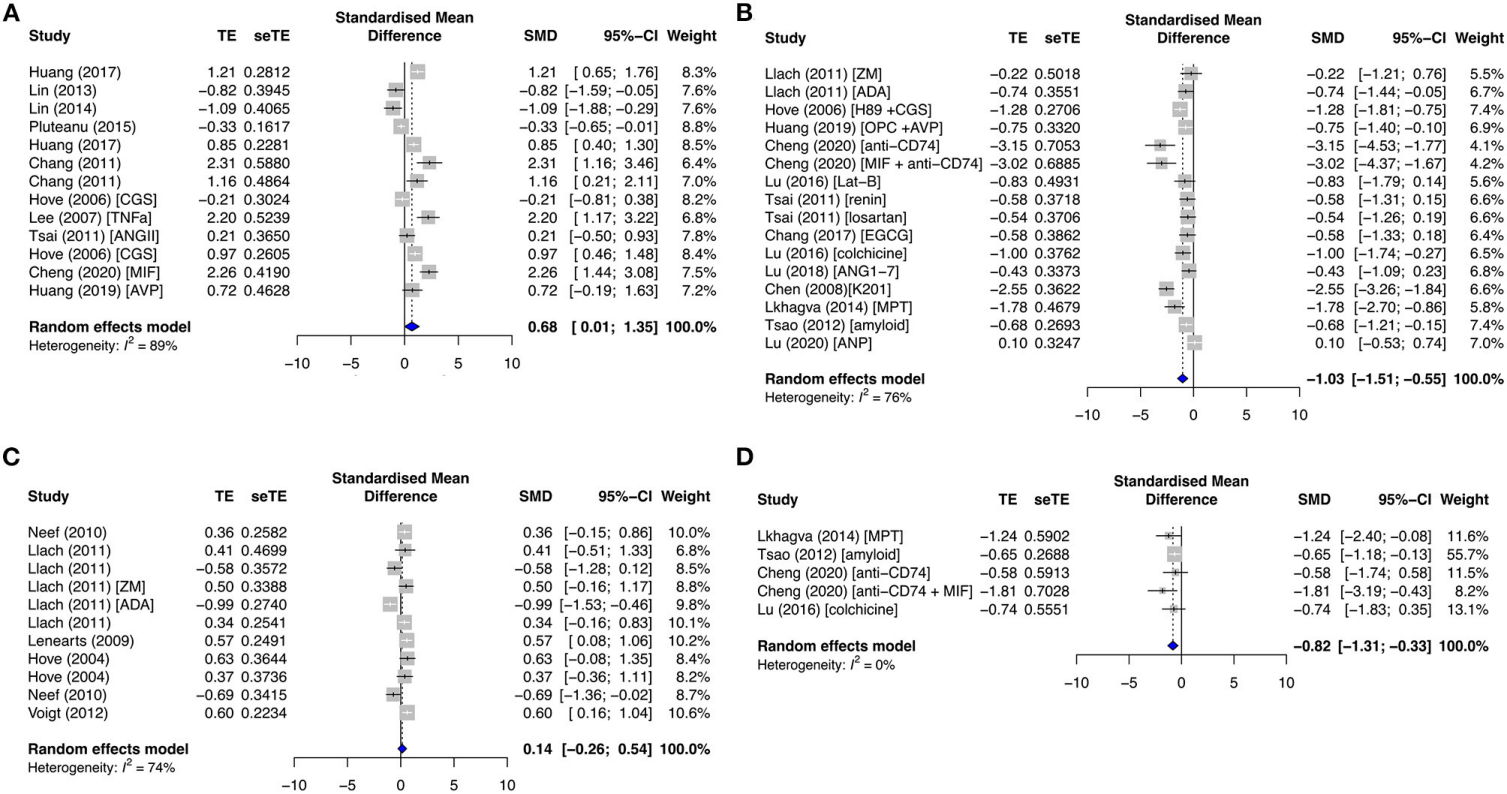
Sodium–calcium current density (I_NCX_) and its protein expression (NCX1), I_NCX_, in the **(A)** primary diseased and proarrhythmic groups, **(B)** antiarrhythmic group, and **(C)** secondary group, and **(D)** NCX1 expression in the primary antiarrhythmic group. TE, estimated treatment effect; seTE, standard error of treatment estimate; SMD, standard mean difference; 95% CI, 95% confidence interval; CGS21680, adenosine 2A agonist; TNFα, tumor necrosis factor-alpha; ANGII, angiotensin II; MIF, macrophage inhibitory factor; AVP, arginine vasopressin; ZM241385, adenosine A2A receptor antagonist; ADA, adenosine deaminase; MPT0E014, histone deacetylase inhibitor.

The extrusion of [Ca^2+^]_i_ for Ca^2+^ recycling is *via* the cardiac NCX. I_NCX_ was significantly enhanced in both the primary diseased and proarrhythmic subgroups (SMD = 0.68; *I*^2^ = 89%; CI = 0.01–1.35; *p* < 0.0001) (Figure 7A) and reduced in the primary antiarrhythmic drug group (SMD = −1.03; *I*^2^ = 76%; CI = −1.51 to −0.55; *p* < 0.0001) (Figure 7B). Likewise, NCX1 protein expression was upregulated in the primary diseased and proarrhythmic subgroup (SMD = 0.43; *I*^2^ = 50%; CI = −0.25–1.10; *p* = 0.0638) (Supplementary Figure 7E) and significantly inhibited in the primary antiarrhythmic subgroup (SMD = −0.82; *I*^2^ = 0%; CI = −1.31 to −0.33; *p* < 0.0001) (Figure 7D). As opposed to the primary group, AF studies demonstrated that I_NCX_ and NCX1 protein expression had mixed results [SMD = 0.14; *I*^2^ = 74%; CI = −0.26–0.54; *p* < 0.0001 (Figure 7C), and SMD = 0.62; *I*^2^ = 61%; CI = −0.29–1.54; *p* = 0.0638 (Supplementary Figure 7H), respectively].

**Figure 8:**
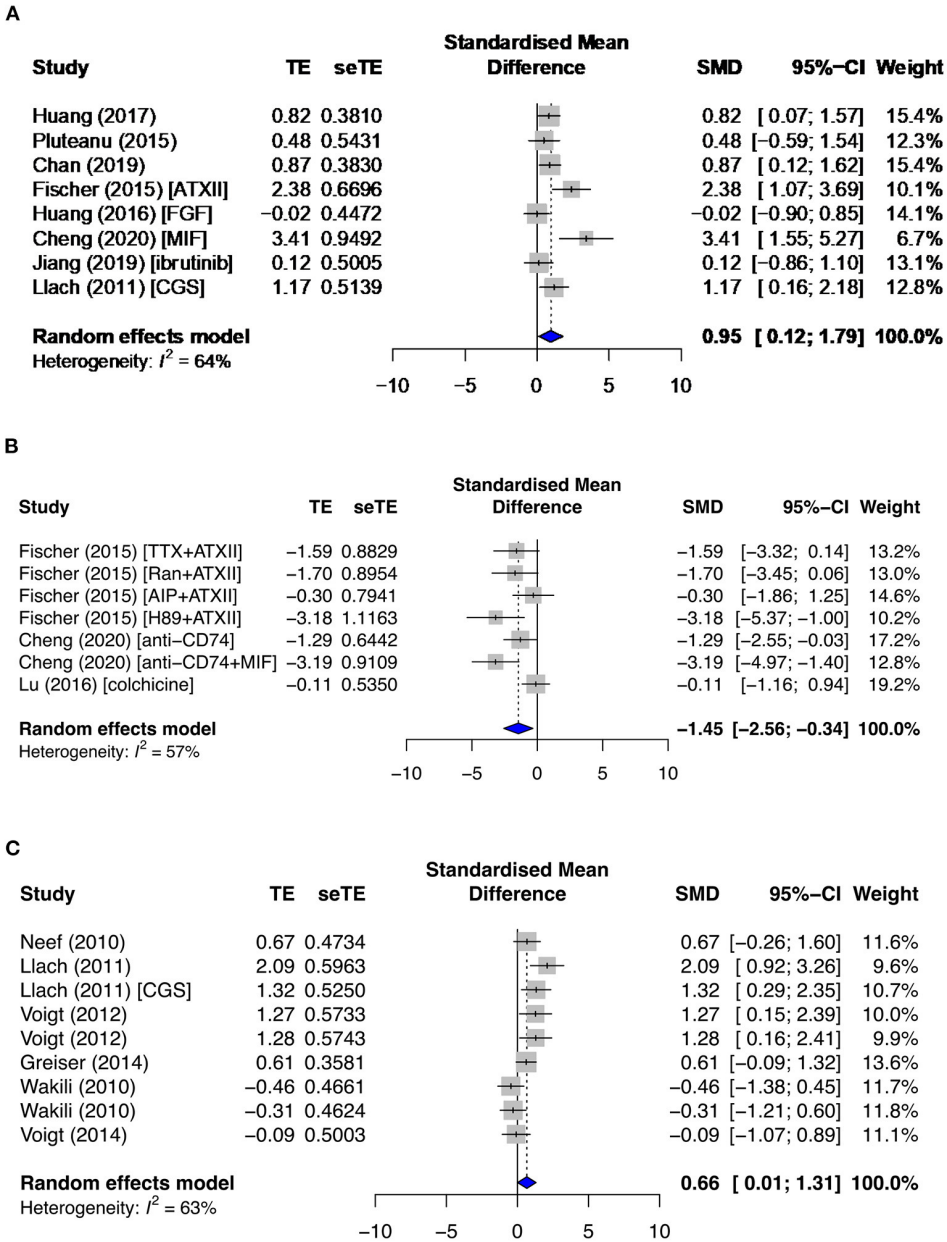
Phosphorylated ryanodine receptor at serine 2808 (pRyR-S2808) protein expression in the **(A)** diseased and proarrhythmic subgroups, **(B)** antiarrhythmic subgroups, and **(C)** secondary AF group. TE, estimated treatment effect; seTE, standard error of treatment estimate; SMD, standard mean difference; 95% CI, 95% confidence interval; ATXII, anemonia viridis toxin 2; FGF23, fibroblast growth factor 23; MIF, macrophage inhibitory factor; CGS21680, adenosine 2A agonist; TTX, tetrodotoxin; Ran, ranolazine; AIP, autocamide-2-related inhibitory peptide; H89, protein kinase inhibitor.

RyR is a major cardiac channel and mediator of the myocardial excitation–contraction coupling. It contains two key phosphorylation sites, serine S2808 (pRyR-S2808) and S2814 (pRyR-S2814). S2808 is phosphorylated by PKA, while S2814 is modulated by CAMKII. Only pRyR-S2808 was significantly affected in both primary and secondary groups, while total RyR (tRyR) and pRyR-S2814 showed no significant changes. pRyR-S2808 expression was increased in the primary diseased and proarrhythmic subgroups (SMD = 0.95; *I*^2^ = 64%; CI = 0.12–1.79; *p* = 0.074) (Figure 8A) and the secondary AF group (SMD = 0.66; *I*^2^ = 63%; CI = 0.01–1.31; *p* < 0.0001) (Figure 8C) but was inhibited by antiarrhythmic drugs (SMD = −1.45; *I*^2^ = 57%; CI = −2.56 to −0.34; *p* = 0.0315) (Figure 8B).

Another pathway for Ca^2+^ recycling is *via* the SERCA2a pump, in which activity is directly controlled by PLN. Few studies reported on SERCA and PLN expression, resulting in inconsistent and non-significant results. SERCA expression was elevated in the primary diseased and proarrhythmic subgroups but substantially reduced in the secondary paroxysmal AF subgroup. Total PLN (tPLN) remained relatively constant in all primary subgroups but was similarly decreased in paroxysmal AF. The results for phosphorylated PLN at sites serine 16 (pPLN-S16) and threonine 17 (pPLN-T17) in both groups were markedly diverse.

**Figure 9:**
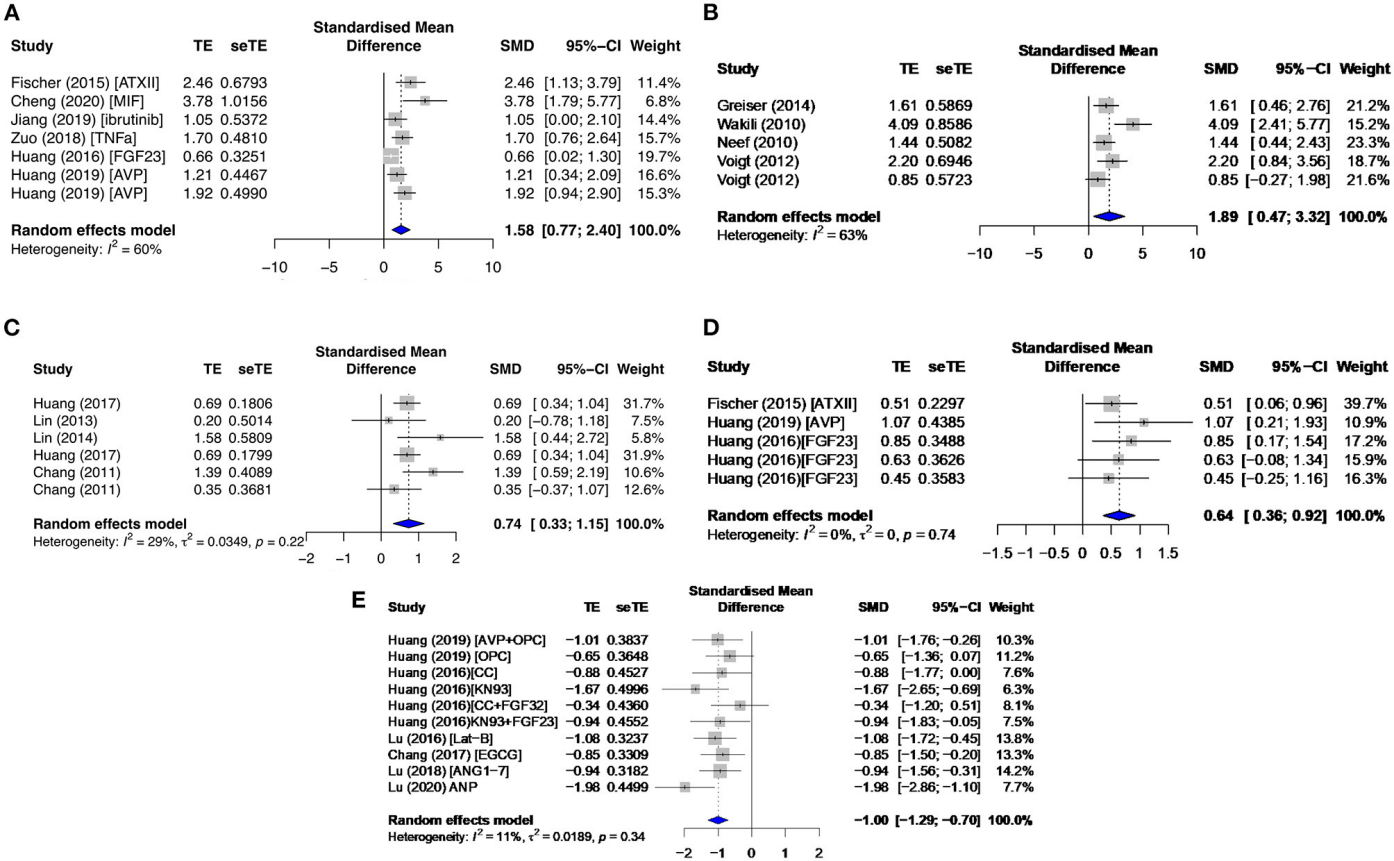
Phosphorylated calcium/calmodulin-dependent protein kinase II at threonine 286 (pCAMKII) and total CAMKII (tCAMKII) protein expressions and late sodium current density (I_Na−Late_) in the primary group. This includes pCAMKII in the **(A)** proarrhythmic subgroup, tCAMKII in the **(B)** AF group, and I_Na−Late_ in the **(C)** diseased, **(D)** proarrhythmic drugs, and **(E)** antiarrhythmic agents. TE, estimated treatment effect; seTE, standard error of treatment estimate; SMD, standard mean difference; 95% CI, 95% confidence interval; ATXII, anemonia viridis toxin 2; FGF23, fibroblast growth factor 23; MIF, macrophage inhibitory factor; AVP, arginine vasopressin; OPC21286, arginine vasopressin antagonists; CC, chelerythrine chloride; Lat-B, latrunculin-B; EGCG, epigallocatechin gallate; ANG1–7, angiotensin 1–7; ANP, atrial natriuretic peptide.

The signaling proteins, CAMKII and PKA, pCAMKII in particular, were affected by proarrhythmic drugs (SMD = 1.58; *I*^2^ = 60%; CI = 0.77–2.40; *p* = 0.0192) (Figure 9A) and antiarrhythmic therapies (SMD = −0.88; *I*^2^ = 66%; CI = −2.04–0.28; *p* = 0.0015), and total CAMKII (t CAMKII) by AF groups (SMD = 1.89; *I*^2^ = 63%; CI = 0.47–3.32; *p* = 0.023) (Figure 9B). Phosphorylated CAMKII had a greater activity than tCAMKII in most subgroups. Only primary studies reported on the sodium current (I_Na_), I_Na−Late_, and I_K_ current densities. However, all data extracted were non-significant and/or inconsistent except for I_Na−Late_. I_Na−Late_ was moderately enhanced in diseased (SMD = 0.74; *I*^2^ = 29%; CI = 0.33–1.15; *p* = 0.2206) (Figure 9C) and proarrhythmic drugs (SMD = 0.64; *I*^2^ = 0%; CI = 0.36–0.92; *p* = 0.7447) (Figure 9D) and significantly antagonized by antiarrhythmic agents (SMD = −1.00; *I*^2^ = 11%; CI = −1.29 to −0.70; *p* = 0.3408) (Figure 9E), with low heterogeneities across all three primary subgroups.

The action of various drugs or reagents in the different experimental models were summarized in Supplementary Tables 4, 5. Additionally, the renin–angiotensin system, targeted by reagents such as angiotensin (ANG) and renin, was most commonly reported. The renin–angiotensin system, which is currently a drug target for hypertension, could also be a potential pharmacological discovery for the treatment and prevention of AF.

## Discussion

### Main Findings

This preclinical systematic review study analyzed 74 articles identified from 446 searched primary and secondary AF prevention articles. Forty-five publications were classified as primary AF prevention studies and 29 others as secondary prevention. To our knowledge, this is the largest study of this kind to explore the association between modulated calcium homeostasis and release events for primary and secondary prevention of AF. Our principal findings are summarized as follows (Table 1).

**Table 1:** The key calcium handling remodeling in the primary prevention group and the secondary diseased group.

	**Ionic currents and calcium proteins/released events**	**Primary diseased group**	**Secondary AF group**
Spontaneous calcium-release events	Calcium transient amplitude (CaTA)	**↑**	**↔**
	Calcium spark amplitude (CaSpA)	**↑**	**↔**
	Calcium transient frequency (CaTF)	n/a	↑
	Calcium spark frequency (CaSpF)	↑	↑
	Sarcoplasmic reticulum (SR) calcium load	↔	↔
	SR calcium leak	n/a	↔
Ionic current densities	L-type calcium current, I_CaL_	↓	↓
	Sodium–calcium exchanger current, I_INCX_	↑	↔
	Sodium current, I_Na_	↔	n/a
	Late sodium current, I_Na−Late_	↑	n/a
	Inward rectifier potassium current, I_K1_	↔	n/a
	Funny current, I_f_	↓	n/a
	Ultrarapid delayed outward rectifier current, I_Kur_	↔	n/a
	Transient outward potassium current, I_to_	↔	n/a
	Transient inward potassium current, I_ti_	↑	n/a
Calcium-handling protein expressions	L-type calcium channel subunit, Ca_v_1.2	↓	↓
	Total ryanodine receptor, tRyR	↔	↔
	Sodium–calcium exchanger 1, NCX1	↑	↑
	tSERCA	↑	↓
	Total phospholamban, tPLN	↔	↓
	tCAMKII	↔	↑
	pRyR-S2808	↑	↑
	pRyR-S2814	↔	↔
	pPLN-S16	↔	↔
	pPLN-T17	↔	↑
	pCAMKII	n/a	↑
	Total protein kinase A, tPKA	↑	n/a

*↑ means increased, ↓ means decreased, ↔ means no significant changes, n/a means no data available*.

*The red color key means that both the primary and secondary diseased groups have a similar trend in the calcium event, protein, or ionic remodeling*.

*tSERCA, total sarco/endoplasmic reticulum calcium-ATPase; tCAMKII, total CAMKII, calcium/calmodulin-dependent protein kinase II; pRyR-S2814, ryanodine receptor phosphorylation at serine 2814; pRyR-S2808, ryanodine receptor phosphorylation at serine 2808; pPLN-S16, phospholamban phosphorylation at serine 16; pPLN-T17, phospholamban phosphorylation at threonine 17; pCAMKII, calcium/calmodulin-dependent protein kinase II phosphorylation at threonine 286*.

With regard to the key Ca^2+^ channels/proteins/mediators, our study found that I_CaL_ was the most widely studied current in both primary and secondary AF prevention, followed by I_NCX_ and RyR2 channels.

- We showed that I_CaL_ was significantly downregulated in primary and secondary diseased groups, which were largely consistent with our results for Ca_v_1.2 protein expression. Antiarrhythmic drugs in the primary group further reduced I_CaL_ significantly.
- Furthermore, the NCX1 protein expression was significantly enhanced in both the primary and secondary diseased groups, but I_NCX_ was only elevated in the primary diseased group.
- In addition, our study demonstrated that the key phosphorylation expression for RyR was enhanced at serine 2808 in both the primary diseased and secondary AF groups, and inhibited in the primary antiarrhythmic drug subgroup. On the other hand, the other key RyR phosphorylation expression at serine 2814 showed no significant changes in both the primary and secondary diseased groups.
- SERCA expression was elevated in the primary diseased and proarrhythmic drug subgroups but substantially reduced in the secondary paroxysmal AF subgroup. tPLN remained relatively constant in all primary subgroups but was decreased in paroxysmal AF.
- Finally, the Ca^2+^ signaling mediator CAMKII was increased in the secondary AF group. With its phosphorylation activity at threonine 286, pCAMII-T286 was significantly raised by proarrhythmic drugs and significantly reduced by antiarrhythmic therapies.

It is noteworthy that there is a growing surge of interest for the late sodium current I_Na−Late_ and its direct effect on arrhythmia. Our study identified many primary preventative publications that showed that I_Na−Late_ was moderately enhanced in the primary diseased and proarrhythmic drug subgroups but significantly antagonized by antiarrhythmic agents, with low heterogeneities across all three subgroups.

As a result of atrial remodeling in the ionic channels and protein/signaling expressions in diseased and AF conditions, we observed changed Ca^2+^ functional activities, i.e., Ca^2+^ spark, Ca^2+^ transient, and Ca^2+^ load/leak. In the primary prevention group, CaSpF, CaTA, and CaSpA were significantly enhanced in the diseased subgroup and decreased by antiarrhythmic drug agents. On the other hand, CaTF and CaSpF were significantly elevated in both the secondary paroxysmal and chronic AF subgroups. Interestingly, we discovered that SR Ca^2+^ load and Ca^2+^ leak remained relatively constant in the primary and secondary subgroups, except when SR Ca^2+^ load was reduced when antiarrhythmic drugs were applied in the primary group. Furthermore, we found that Ca^2+^ leak was raised by proarrhythmic agents and antagonized by antiarrhythmic agents in the primary group.

### Potential Mechanisms for Primary AF Diseases

The pathophysiological mechanism that causes spontaneous sarcoplasmic calcium release in the primary group involves the downregulation of I_CaL_ and dysfunction of the Ca^2+^-handling proteins, in particular pRyR at S2808. The enhanced pRyR-S2808 activity may increase the frequency of the Ca^2+^ spark due to calcium-induced calcium releases. This would eventually lead to a high Ca^2+^ level in the cytosol and enhanced trigger activities *via* the forward mode of I_NCX_, which was demonstrated in this review as the elevation of I_NCX_ activity. Furthermore, the enhancement of I_Na−Late_ was shown to potentially play a more significant role in the generation of arrhythmia in our review and recent studies (95–97). The reduction in I_CaL_ was predicted to result in reduced SR load and diminished spontaneous activity. On the contrary, the downregulation of Ca_v_1.2 current and protein expression has resulted in SCaEs, which could possibly be due to cell compensation (98, 99). Overall, this interplay led to an overload of Ca^2+^ in the cell, potentially causing AF.

### Abnormal Ca^2+^ Activity in AF

The electrophysiological remodeling induced in the fibrillating atria and its molecular basis were extensively reviewed. Recent and past AF studies (95–97) have suggested that I_CaL_ density was downregulated, together with the reduction of its protein expression, Ca_v_1.2. Strangely, reduced I_CaL_ density did not diminish the SR load; it remained unchanged (96). In contrast to the primary AF disease, consistently reduced SERCA levels were identified, reducing the releasable SR Ca^2+^ in the cytosol (98, 99).

On the other hand, some studies observed that the SR Ca^2+^ leak and activity of RyR were consistently upregulated (97, 100). Their observation justified the increase in SCaEs. The NCX expression was also increased, in contrast to what we saw earlier in the primary mechanism (100). The increased NCX expression could also account for the increase in frequencies of both the Ca^2+^ sparks and Ca^2+^ transients. The overextrusion of Ca^2+^ explains the unchanged CaSpA and CaTA.

We have shown that I_CaL_ and NCX1 protein are the primary remodeling targets identified, and this leads to spontaneous calcium activity due to its interrelationship with the SR proteins. The conclusion of the secondary group meta-analysis aligns perfectly with the AF mechanism provided by Madsen et al. (34). On the other hand, the proposed primary mechanism is the best agglomeration of the mechanisms acquired from individual modifiable/non-modifiable risk factors associated with AF.

### Limitations

This review aims to provide a better understanding of the mechanisms involved in the [Ca^2+^]_i_ homeostasis within atrial cardiomyocytes and compare their activities among the primary and secondary AF subgroups. Although this review has comprehensively compiled the Ca^2+^ activity from inception to date, it still presents several limitations. The activity of SERCA and PLN appears to be unclear. It is certain that I_CaL_ was reduced in the primary diseased and secondary AF groups. Surprisingly, the SR Ca^2+^ load–leak relationship was unaltered in the primary diseased and secondary AF groups with high heterogeneities. This could be influenced by a variety of non-controllable factors, such as the variability in the animal and human studies at various stages of AF or diseased states, the type and strength of pharmacological agents applied, and the different experimental settings and methodologies (101, 102).

## Conclusion

Our study identified that I_CaL_ is reduced in both primary and secondary diseased groups. Furthermore, pRyR-S2808 and NCX1 protein expression are enhanced. The remodeling leads to elevated Ca^2+^ functional activities, such as the frequencies or amplitude of Ca^2+^ spark and Ca^2+^ transient. The main difference identified between primary and secondary diseased groups is the SERCA expression, which is elevated in the primary diseased group and substantially reduced in the secondary paroxysmal AF subgroup. We believe our study will add new evidence to AF mechanisms and treatment targets.

## Data Availability Statement

The datasets presented in this study can be found in online repositories. The names of the repository/repositories and accession number(s) can be found in the article/Supplementary Material.

## Author Contributions

SF and SA undertook data extraction, post-processing, and analysis, as well as drafting the manuscript. MG assisted in assessing the quality of the included studies. JZ guided the project and revised the manuscript. All authors contributed to the article and approved the submitted version.

## Conflict of Interest

The authors declare that the research was conducted in the absence of any commercial or financial relationships that could be construed as a potential conflict of interest.
