# Supplementary material for: Modulated Calcium Homeostasis and Release Events Under Atrial Fibrillation and Its Risk Factors: A Meta-Analysis
Source: Front Cardiovasc Med. 2021 Jul 20;8:662914. doi: 10.3389/fcvm.2021.662914 (PMC8329373; doi:10.3389/fcvm.2021.662914)
Supplement: Supplementary file 1 [file Data_Sheet_1.docx]

**Supplementary Tables**

**Supplementary Table S1.** PRISMA 2009 Checklist

| **Section/topic** | **#** | **Checklist item** | **Reported on page #** |
| --- | --- | --- | --- |
| **TITLE** | | |  |
| Title | 1 | Identify the report as a systematic review, meta-analysis, or both. | 1 |
| **ABSTRACT** | | |  |
| Structured summary | 2 | Provide a structured summary including, as applicable: background; objectives; data sources; study eligibility criteria, participants, and interventions; study appraisal and synthesis methods; results; limitations; conclusions and implications of key findings; systematic review registration number. | 1 |
| **INTRODUCTION** | | |  |
| Rationale | 3 | Describe the rationale for the review in the context of what is already known. | 2 |
| Objectives | 4 | Provide an explicit statement of questions being addressed with reference to participants, interventions, comparisons, outcomes, and study design (PICOS). | 2 |
| **METHODS** | | |  |
| Protocol and registration | 5 | Indicate if a review protocol exists, if and where it can be accessed (e.g., Web address), and, if available, provide registration information including registration number. | 3 |
| Eligibility criteria | 6 | Specify study characteristics (e.g., PICOS, length of follow-up) and report characteristics (e.g., years considered, language, publication status) used as criteria for eligibility, giving rationale. | 3 |
| Information sources | 7 | Describe all information sources (e.g., databases with dates of coverage, contact with study authors to identify additional studies) in the search and date last searched. | 3 |
| Search | 8 | Present full electronic search strategy for at least one database, including any limits used, such that it could be repeated. | 3 |
| Study selection | 9 | State the process for selecting studies (i.e., screening, eligibility, included in systematic review, and, if applicable, included in the meta-analysis). | 3 |
| Data collection process | 10 | Describe method of data extraction from reports (e.g., piloted forms, independently, in duplicate) and any processes for obtaining and confirming data from investigators. | 4 |
| Data items | 11 | List and define all variables for which data were sought (e.g., PICOS, funding sources) and any assumptions and simplifications made. | 4 |
| Risk of bias in individual studies | 12 | Describe methods used for assessing risk of bias of individual studies (including specification of whether this was done at the study or outcome level), and how this information is to be used in any data synthesis. | 4 |
| Summary measures | 13 | State the principal summary measures (e.g., risk ratio, difference in means). | 4-5 |
| Synthesis of results | 14 | Describe the methods of handling data and combining results of studies, if done, including measures of consistency (e.g., I^2^) for each meta-analysis. | 4-5 |

Adapted from: Moher D, Liberati A, Tetzlaff J, Altman DG, The PRISMA Group (2009) Preferred Reporting Items for Systematic Reviews and Meta-Analyses: The PRISMA Statement. PLoS Med 6(7): e1000097. https://doi.org/10.1371/journal.pmed.1000097

**Supplementary Table S2.** Risk of Bias Assessment for Selected Studies

| **Study** | **A** | **B** | **C** | **D** | **E** | **F** | **G** | **H** | **I** | **J** | **Total** |
| --- | --- | --- | --- | --- | --- | --- | --- | --- | --- | --- | --- |
| Macquaide N, 2015 | 1 |  |  |  |  | 1 | 1 |  | 1 | 1 | 5 |
| Opel A, 2015 | 1 | 1 |  |  |  | 1 | 1 |  | 1 | 1 | 6 |
| Llach A, 2011 | 1 | 1 |  |  |  | 1 | 1 |  | 1 | 1 | 6 |
| Zhang H, 2015 | 1 | 1 |  |  |  | 1 | 1 |  | 1 | 1 | 6 |
| Gassanov N, 2006 | 1 |  |  |  |  |  | 1 |  | 1 | 1 | 4 |
| Hartmann N, 2017 | 1 | 1 |  |  |  |  | 1 |  | 1 | 1 | 5 |
| Yan J, 2018 | 1 |  |  |  |  | 1 | 1 |  | 1 | 1 | 5 |
| Zuo S, 2018 | 1 | 1 |  |  |  |  |  |  | 1 | 1 | 4 |
| Yang Y, 2018 | 1 | 1 | 1 | 1 |  | 1 | 1 |  | 1 | 1 | 8 |
| Hartmann N, 2016 | 1 | 1 |  |  |  | 1 | 1 |  | 1 | 1 | 6 |
| Pasqualin C, 2018 | 1 | 1 | 1 |  |  |  | 1 |  | 1 | 1 | 6 |
| Hove-Madsne L, 2006 | 1 | 1 |  |  |  |  | 1 |  | 1 | 1 | 5 |
| Lenaerts I, 2009 | 1 |  |  |  |  | 1 | 1 |  | 1 | 1 | 5 |
| Hove-Madsne L, 2004 | 1 | 1 |  |  | 1 | 1 | 1 |  | 1 | 1 | 7 |
| Neef S, 2010 | 1 | 1 |  |  |  |  | 1 |  | 1 | 1 | 5 |
| Huang JH, 2019 | 1 | 1 |  |  |  | 1 | 1 |  | 1 | 1 | 6 |
| Kanaporis G, 2017 | 1 | 1 |  |  |  | 1 | 1 |  | 1 | 1 | 6 |
| Kanaporis G, 2019 | 1 | 1 |  |  |  | 1 | 1 |  | 1 | 1 | 6 |
| Huang SY, 2016 | 1 | 1 |  |  |  | 1 | 1 |  | 1 | 1 | 6 |
| Lu YY, 2016 | 1 | 1 |  |  |  | 1 | 1 |  | 1 | 1 | 6 |
| Greiser M, 2014 | 1 | 1 |  |  |  |  | 1 |  | 1 | 1 | 5 |
| Liang X, 2008 | 1 | 1 |  |  |  |  | 1 |  | 1 | 1 | 5 |
| Tazmini K, 2020 | 1 | 1 |  |  |  | 1 | 1 |  | 1 | 1 | 6 |
| Cheng WL, 2020 | 1 | 1 |  |  |  |  | 1 |  | 1 | 1 | 5 |
| Lee SH, 2007 | 1 | 1 |  |  |  | 1 | 1 |  | 1 | 1 | 6 |
| Henry AD, 2018 | 1 | 1 |  |  |  |  | 1 |  | 1 | 1 | 5 |
| Chiang DY, 2014 | 1 | 1 |  |  |  | 1 | 1 |  | 1 | 1 | 6 |
| Musa H, 2013 | 1 | 1 |  |  |  | 1 | 1 |  | 1 | 1 | 6 |
| Li N, 2012 | 1 | 1 |  |  |  |  | 1 |  | 1 | 1 | 5 |
| Mancarella S, 2008 | 1 | 1 |  |  |  | 1 | 1 |  | 1 | 1 | 6 |
| Zhang YH, 2013 | 1 |  |  |  |  |  | 1 |  | 1 | 1 | 4 |
| Jones SA, 2008 | 1 | 1 |  |  |  |  | 1 |  | 1 | 1 | 5 |
| Tsai CT, 2011 | 1 | 1 |  |  |  | 1 | 1 |  | 1 | 1 | 6 |
| Logantha SJ, 2010 | 1 | 1 |  |  |  | 1 | 1 |  | 1 | 1 | 6 |
| Zhang Y, 2010 | 1 | 1 |  |  |  | 1 | 1 |  | 1 | 1 | 6 |
| Wang T, 2019 | 1 |  |  |  |  |  | 1 |  | 1 | 1 | 4 |
| Jiang LL, 2019 | 1 |  |  |  |  | 1 | 1 |  | 1 | 1 | 5 |
| Tsai WC, 2013 | 1 | 1 |  |  |  | 1 | 1 |  | 1 | 1 | 6 |
| Chang JH, 2017 | 1 | 1 |  |  |  | 1 | 1 |  | 1 | 1 | 6 |
| Shafaattalab S, 2019 | 1 | 1 |  |  |  |  | 1 |  | 1 | 1 | 5 |
| Lu YY, 2016 | 1 | 1 |  |  |  |  | 1 |  | 1 | 1 | 5 |
| Lin YK, 2014 | 1 | 1 |  |  |  |  | 1 |  | 1 | 1 | 5 |
| Lu YY, 2018 | 1 | 1 |  |  |  | 1 | 1 |  | 1 | 1 | 6 |
| Zhou WP, 2018 | 1 | 1 |  |  |  | 1 | 1 |  | 1 | 1 | 6 |
| Suenari K, 2011 | 1 | 1 |  |  |  | 1 | 1 |  | 1 | 1 | 6 |
| Fischer TH, 2015 | 1 | 1 |  |  |  |  | 1 |  | 1 | 1 | 5 |
| Pluteanu F, 2015 | 1 | 1 |  |  |  | 1 | 1 |  | 1 | 1 | 6 |
| Lin YK, 2013 | 1 | 1 |  |  |  | 1 | 1 |  | 1 | 1 | 6 |
| Chen YJ, 2008 | 1 | 1 |  |  |  | 1 | 1 |  | 1 | 1 | 6 |
| Voigt N, 2013 | 1 | 1 |  |  |  | 1 | 1 |  | 1 | 1 | 6 |
| Coutu P, 2006 | 1 | 1 |  |  |  | 1 | 1 |  | 1 | 1 | 6 |
| Rios PEB, 2016 | 1 | 1 |  |  |  | 1 | 1 |  | 1 | 1 | 6 |
| Uma H, 2019 | 1 | 1 |  |  |  | 1 | 1 |  | 1 | 1 | 6 |
| Tsai CT, 2011 | 1 | 1 |  |  |  |  | 1 |  | 1 | 1 | 5 |
| Wongcharoen W, 2006 | 1 | 1 |  |  |  | 1 | 1 |  | 1 | 1 | 6 |
| Wakili R, 2010 | 1 | 1 |  |  |  | 1 | 1 |  | 1 | 1 | 6 |
| Qi X, 2008 | 1 | 1 |  |  |  |  | 1 |  | 1 | 1 | 5 |
| Lkhagva B, 2014 | 1 | 1 |  |  |  | 1 | 1 |  | 1 | 1 | 6 |
| Lebek S, 2018 | 1 | 1 |  |  |  | 1 | 1 |  | 1 | 1 | 6 |
| Voigt N, 2012 | 1 | 1 |  |  |  | 1 | 1 |  | 1 | 1 | 6 |
| Chan YH, 2019 | 1 | 1 | 1 |  |  | 1 | 1 |  | 1 | 1 | 7 |
| Tsao HM, 2012 | 1 | 1 |  |  |  | 1 | 1 |  | 1 | 1 | 6 |
| Lin YK, 2012 | 1 | 1 |  |  |  |  | 1 |  | 1 | 1 | 5 |
| Workman AJ, 2001 | 1 | 1 |  |  |  | 1 | 1 |  | 1 | 1 | 6 |
| Lenaerts I, 2011 | 1 | 1 |  |  |  | 1 | 1 |  | 1 | 1 | 6 |
| Voigt N, 2014 | 1 | 1 |  |  |  | 1 | 1 |  | 1 | 1 | 6 |
| Greer SA, 2020 | 1 | 1 |  |  |  |  | 1 |  | 1 | 1 | 5 |
| Chen WT, 2015 | 1 | 1 |  |  |  | 1 | 1 |  | 1 | 1 | 6 |
| Huang SY, 2017 | 1 | 1 |  |  |  | 1 | 1 |  | 1 | 1 | 6 |
| Herraiz MA, 2015 | 1 | 1 |  |  |  |  | 1 |  | 1 | 1 | 5 |
| Chang SL, 2011 | 1 | 1 |  |  |  | 1 | 1 |  | 1 | 1 | 6 |
| Lu YY, 2020 | 1 | 1 |  |  |  | 1 | 1 |  | 1 | 1 | 6 |
| Suenari K, 2011 | 1 | 1 |  |  |  | 1 | 1 |  | 1 | 1 | 6 |
| Gu J, 2013 | 1 | 1 |  |  |  | 1 | 1 |  | 1 | 1 | 6 |

Studies fulfilling the criteria of: A, peer reviewed publication; B, control of temperature; C, random allocation to treatment or control; D, blinded induction of model (group randomly after the induction of model); E, blinded assessment of outcome; F, use of anesthetic without significant intrinsic Ca^2+^ activity; G, appropriate animal model; H, sample size calculation; I, compliance with animal welfare regulations [preoperative anesthesia, postoperative analgesia, nutrition, environment (temperature, humidity, circadian rhythm), and euthanasia]; J, statement of potential conflict of interests.

**Supplementary Table S3.** List of 372 full text excluded articles and the reasons for their exclusion.

| **25 did not report on atrial fibrillation:**   1. Aistrup GL, Arora R, Grubb S, et al. Triggered intracellular calcium waves in dog and human left atrial myocytes from normal and failing hearts. Cardiovasc Res. 2017;113(13):1688-1699. doi:10.1093/cvr/cvx167. 2. Kim JC, Son MJ, Wang J, Woo SH. Regulation of cardiac Ca2+ and ion channels by shear mechanotransduction. Arch Pharm Res. 2017;40(7):783-795. doi:10.1007/s12272-017-0929-7. 3. Fujii A, Inoue K, Nagai T, et al. Clinical Utility of Atrial Electromechanical Conduction Time Measured with Speckle Tracking Echocardiography after Catheter Ablation in Patients with Atrial Fibrillation: A Validation Study with Electroanatomical Mapping. Echocardiography. 2016;33(9):1317-1325. doi:10.1111/echo.13259. 4. Efe TH, Cimen T, Ertem AG, et al. Atrial Electromechanical Properties in Inflammatory Bowel Disease. Echocardiography. 2016;33(9):1309-1316. doi:10.1111/echo.13261. 5. Shiferaw Y, Aistrup GL, Louch WE, Wasserstrom JA. Remodeling Promotes Proarrhythmic Disruption of Calcium Homeostasis in Failing Atrial Myocytes. Biophys J. 2020;118(2):476-491. doi:10.1016/j.bpj.2019.12.012. 6. Hannon JD, Housmans PR. Ca2+: is there something new for the cardiovascular anesthesiologist?. Curr Opin Anaesthesiol. 2009;22(1):114-120. doi:10.1097/ACO.0b013e32831a43c7. 7. Mao T, Zhang J, Qiao Y, Liu B, Zhang S. Uncovering Synergistic Mechanism of Chinese Herbal Medicine in the Treatment of Atrial Fibrillation with Obstructive Sleep Apnea Hypopnea Syndrome by Network Pharmacology. Evid Based Complement Alternat Med. 2019;2019:8691608. Published 2019 Dec 23. doi:10.1155/2019/8691608. 8. Zimering MB, Pan Z. Increased Neuronal Depolarization Evoked by Autoantibodies in Diabetic Obstructive Sleep Apnea: Role for Inflammatory Protease(s) in Generation of Neurotoxic Immunoglobulin Fragment. J Endocrinol Diabetes. 2017;4(1):10.15226/2374-6890/4/1/00168. doi:10.15226/2374-6890/4/1/00168. 9. Murata M, Cingolani E, McDonald AD, Donahue JK, Marbán E. Creation of a genetic calcium channel blocker by targeted gem gene transfer in the heart. Circ Res. 2004;95(4):398-405. doi:10.1161/01.RES.0000138449.85324.c5. 10. Francia P, Adduci C, Ricotta A, et al. Common genetic variants in selected Ca²⁺ signaling genes and the risk of appropriate ICD interventions in patients with heart failure. J Interv Card Electrophysiol. 2013;38(3):169-177. doi:10.1007/s10840-013-9827-1. 11. Dixen U, Wallevik L, Hansen MS, et al. Prolonged signal-averaged P wave duration as a prognostic marker for morbidity and mortality in patients with congestive heart failure. Scand Cardiovasc J. 2003;37(4):193-198. doi:10.1080/14017430310002202. 12. Cha TJ, Ehrlich JR, Zhang L, et al. Dissociation between ionic remodeling and ability to sustain atrial fibrillation during recovery from experimental congestive heart failure. Circulation. 2004;109(3):412-418. doi:10.1161/01.CIR.0000109501.47603.0C. 13. Li D, Melnyk P, Feng J, et al. Effects of experimental heart failure on atrial cellular and ionic electrophysiology. Circulation. 2000;101(22):2631-2638. doi:10.1161/01.cir.101.22.2631. 14. Jones RJ, Brace SR, Vander Tuin EL. Probable propafenone-induced transient global amnesia. Ann Pharmacother. 1995;29(6):586-590. doi:10.1177/106002809502900606. 15. Shepard RK, Natale A, Stambler BS, Wood MA, Gilligan DM, Ellenbogen KA. Physiology of the escape rhythm after radiofrequency atrioventricular junctional ablation. Pacing Clin Electrophysiol. 1998;21(5):1085-1092. doi:10.1111/j.1540-8159.1998.tb00154.x. 16. Leow MK, Loh KC, Kon WY, Wong DE, Tan BS, Soon PC. Clinical utility of selective intra-arterial calcium-stimulated hepatic venous sampling in regionalisation of insulinomas--the Singapore experience. Ann Acad Med Singapore. 2003;32(1):86-91. 17. Nof E, Belhassen B, Arad M, et al. Postpacing abnormal repolarization in catecholaminergic polymorphic ventricular tachycardia associated with a mutation in the cardiac ryanodine receptor gene. Heart Rhythm. 2011;8(10):1546-1552. doi:10.1016/j.hrthm.2011.05.016. 18. Ling LH, Khammy O, Byrne M, et al. Irregular rhythm adversely influences calcium handling in ventricular myocardium: implications for the interaction between heart failure and atrial fibrillation. Circ Heart Fail. 2012;5(6):786-793. doi:10.1161/CIRCHEARTFAILURE.112.968321. 19. Yasushige Shingu, Suguru Kubota, Satoru Wakasa, Noriyoshi Ebuoka, Daisuke Mori, Tomonori Ooka, Tsuyoshi Tachibana, Yoshiro Matsui, Left-ventricular electromechanical delay is prolonged in patients with postoperative atrial fibrillation, European Journal of Cardio-Thoracic Surgery, Volume 39, Issue 5, May 2011, Pages 684–688, doi: 10.1016/j.ejcts.2010.08.015. 20. Schwarzwald CC, Bonagura JD, Luis-Fuentes V. Effects of diltiazem on hemodynamic variables and ventricular function in healthy horses. J Vet Intern Med. 2005;19(5):703-711. doi:10.1892/0891-6640(2005)19[703:eodohv]2.0.co;2. 21. Medvedowsky JL, Barnay C, Arnaud C, Bonet P, Quittet F, Lam LF. Le bépridil dans le traitement des tachycardies paroxystiques supraventriculaires [Bepridil in the treatment of supraventricular paroxysmal tachycardias]. Arch Mal Coeur Vaiss. 1985;78 Spec No:67-74. 22. Han L, Li J. Canonical transient receptor potential 3 channels in atrial fibrillation. Eur J Pharmacol. 2018;837:1-7. doi:10.1016/j.ejphar.2018.08.030. 23. Nikolova-Krstevski V, Wagner S, Yu ZY, et al. Endocardial TRPC-6 Channels Act as Atrial Mechanosensors and Load-Dependent Modulators of Endocardial/Myocardial Cross-Talk. JACC Basic Transl Sci. 2017;2(5):575-590. Published 2017 Oct 30. doi:10.1016/j.jacbts.2017.05.006. 24. Tadevosyan A, Xiao J, Surinkaew S, et al. Intracellular Angiotensin-II Interacts With Nuclear Angiotensin Receptors in Cardiac Fibroblasts and Regulates RNA Synthesis, Cell Proliferation, and Collagen Secretion. J Am Heart Assoc. 2017;6(4):e004965. Published 2017 Apr 5. doi:10.1161/JAHA.116.004965. 25. Yeh YH, Wakili R, Qi XY, et al. Calcium-handling abnormalities underlying atrial arrhythmogenesis and contractile dysfunction in dogs with congestive heart failure. Circ Arrhythm Electrophysiol. 2008;1(2):93-102. doi:10.1161/CIRCEP.107.754788. | |
| --- | --- |
| **24 articles reported on genes or miRNA:**   1. Herraiz-Martínez A, Llach A, Tarifa C, Gandía J, Jiménez-Sabado V, Lozano-Velasco E, Serra SA, Vallmitjana A, Vázquez Ruiz de Castroviejo E, Benítez R, Aranega A, Muñoz-Guijosa C, Franco D, Cinca J, Hove-Madsen L. The 4q25 variant rs13143308T links risk of atrial fibrillation to defective calcium homoeostasis. Cardiovasc Res. 2019 Mar 1;115(3):578-589. doi: 10.1093/cvr/cvy215. 2. Cañón S, Caballero R, Herraiz-Martínez A, Pérez-Hernández M, López B, Atienza F, Jalife J, Hove-Madsen L, Delpón E, Bernad A. miR-208b upregulation interferes with calcium handling in HL-1 atrial myocytes: Implications in human chronic atrial fibrillation. J Mol Cell Cardiol. 2016 Oct;99:162-173. doi: 10.1016/j.yjmcc.2016.08.012. 3. Tsai FC, Lin YC, Chang SH, et al. Differential left-to-right atria gene expression ratio in human sinus rhythm and atrial fibrillation: Implications for arrhythmogenesis and thrombogenesis. Int J Cardiol. 2016;222:104-112. doi:10.1016/j.ijcard.2016.07.103. 4. Ni L, Scott L Jr, Campbell HM, et al. Atrial-Specific Gene Delivery Using an Adeno-Associated Viral Vector. Circ Res. 2019;124(2):256-262. doi:10.1161/CIRCRESAHA.118.313811. 5. Chiang DY, Kongchan N, Beavers DL, et al. Loss of microRNA-106b-25 cluster promotes atrial fibrillation by enhancing ryanodine receptor type-2 expression and calcium release. Circ Arrhythm Electrophysiol. 2014;7(6):1214-1222. doi:10.1161/CIRCEP.114.001973. 6. Zhu Y, Feng Z, Cheng W, Xiao Y. MicroRNA‑34a mediates atrial fibrillation through regulation of Ankyrin‑B expression. Mol Med Rep. 2018;17(6):8457-8465. doi:10.3892/mmr.2018.8873. 7. Nadadur RD, Broman MT, Boukens B, et al. Pitx2 modulates a Tbx5-dependent gene regulatory network to maintain atrial rhythm. Sci Transl Med. 2016;8(354):354ra115. doi:10.1126/scitranslmed.aaf4891. 8. Chen J, Xu S, Li W, et al. Nkx2.5 insufficiency leads to atrial electrical remodeling through Wnt signaling in HL-1 cells. Exp Ther Med. 2019;18(6):4631-4636. doi:10.3892/etm.2019.8134. 9. Takano K, Liu D, Tarpey P, et al. An X-linked channelopathy with cardiomegaly due to a CLIC2 mutation enhancing ryanodine receptor channel activity. Hum Mol Genet. 2012;21(20):4497-4507. doi:10.1093/hmg/dds292. 10. Kirchhof P, Kahr PC, Kaese S, et al. PITX2c is expressed in the adult left atrium, and reducing Pitx2c expression promotes atrial fibrillation inducibility and complex changes in gene expression. Circ Cardiovasc Genet. 2011;4(2):123-133. doi:10.1161/CIRCGENETICS.110.958058. 11. Ralph F Bosch, Constanze R Scherer, Norman Rüb, et.al. Molecular mechanisms of early electrical remodeling: transcriptional downregulation of ion channel subunits reduces ICa,L and Ito in rapid atrial pacing in rabbits. Journal of the American College of Cardiology. 2003; 41(5): 858-869. doi: 10.1016/S0735-1097(02)02922-4. 12. Gaborit N, Steenman M, Lamirault G, et al. Human atrial ion channel and transporter subunit gene-expression remodeling associated with valvular heart disease and atrial fibrillation. Circulation. 2005;112(4):471-481. doi:10.1161/CIRCULATIONAHA.104.506857. 13. Zhang JC, Wu HL, Chen Q, et al. Calcium-Mediated Oscillation in Membrane Potentials and Atrial-Triggered Activity in Atrial Cells of Casq2R33Q/R33Q Mutation Mice. Front Physiol. 2018;9:1447. Published 2018 Nov 2. doi:10.3389/fphys.2018.01447. 14. Zhang D, Hu X, Li J, et al. Converse role of class I and class IIa HDACs in the progression of atrial fibrillation. J Mol Cell Cardiol. 2018;125:39-49. doi:10.1016/j.yjmcc.2018.09.010. 15. Glukhov AV, Kalyanasundaram A, Lou Q, et al. Calsequestrin 2 deletion causes sinoatrial node dysfunction and atrial arrhythmias associated with altered sarcoplasmic reticulum calcium cycling and degenerative fibrosis within the mouse atrial pacemaker complex1. Eur Heart J. 2015;36(11):686-697. doi:10.1093/eurheartj/eht452. 16. Sood S, Chelu MG, van Oort RJ, et al. Intracellular calcium leak due to FKBP12.6 deficiency in mice facilitates the inducibility of atrial fibrillation. Heart Rhythm. 2008;5(7):1047-1054. doi:10.1016/j.hrthm.2008.03.030. 17. Lozano-Velasco E, Hernández-Torres F, Daimi H, et al. Pitx2 impairs calcium handling in a dose-dependent manner by modulating Wnt signalling. Cardiovasc Res. 2016;109(1):55-66. doi:10.1093/cvr/cvv207. 18. Lou Q, Belevych AE, Radwański PB, et al. Alternating membrane potential/calcium interplay underlies repetitive focal activity in a genetic model of calcium-dependent atrial arrhythmias. J Physiol. 2015;593(6):1443-1458. doi:10.1113/jphysiol.2014.280784. 19. Bögeholz N, Pauls P, Kaese S, et al. Triggered activity in atrial myocytes is influenced by Na+/Ca2+ exchanger activity in genetically altered mice. J Mol Cell Cardiol. 2016;101:106-115. doi:10.1016/j.yjmcc.2016.11.004. 20. Hong, Chang-Soo & Kwon, Soon-Jae & Cho, Myeong-Chan & Kwak, et. al. Overexpression of junctate induces cardiac hypertrophy and arrhythmia via altered calcium handling. Journal of molecular and cellular cardiology. 2008; 44: 672-82. 10.1016/j.yjmcc.2008.01.012. 21. Tao Y, Zhang M, Li L, et al. Pitx2, an atrial fibrillation predisposition gene, directly regulates ion transport and intercalated disc genes. Circ Cardiovasc Genet. 2014;7(1):23-32. doi:10.1161/CIRCGENETICS.113.000259. 22. Kao YH, Hsu JC, Chen YC, et al. ZFHX3 knockdown increases arrhythmogenesis and dysregulates calcium homeostasis in HL-1 atrial myocytes. Int J Cardiol. 2016;210:85-92. doi:10.1016/j.ijcard.2016.02.091. 23. Deng C, Rao F, Wu S, et al. Pharmacological effects of carvedilol on T-type calcium current in murine HL-1 cells. Eur J Pharmacol. 2009;621(1-3):19-25. doi:10.1016/j.ejphar.2009.08.032. 24. van Ouwerkerk, A.F., Bosada, F.M., van Duijvenboden, K. et al. Identification of atrial fibrillation associated genes and functional non-coding variants. Nat Commun 10, 4755 (2019). | |
| **23 articles did not study on atrial cells, instead experimented on a tissue or organelle level:**   1. Yin D, Chen M, Yang N, et al. Role of apamin-sensitive small conductance calcium-activated potassium currents in long-term cardiac memory in rabbits. Heart Rhythm. 2018;15(5):761-769. doi:10.1016/j.hrthm.2018.01.016. 2. Hsueh CH, Chang PC, Hsieh YC, Reher T, Chen PS, Lin SF. Proarrhythmic effect of blocking the small conductance calcium activated potassium channel in isolated canine left atrium. Heart Rhythm. 2013;10(6):891-898. doi:10.1016/j.hrthm.2013.01.033. 3. Ono N, Hayashi H, Kawase A, et al. Spontaneous atrial fibrillation initiated by triggered activity near the pulmonary veins in aged rats subjected to glycolytic inhibition. Am J Physiol Heart Circ Physiol. 2007;292(1):H639-H648. doi:10.1152/ajpheart.00445.2006. 4. Jannie Ausma, Gerrit D Dispersyn, Hans Duimel, et. al. Changes in Ultrastructural Calcium Distribution in Goat Atria During Atrial Fibrillation. Journal of Molecular and Cellular Cardiology. 2000; 32(3): 355-364,doi: 10.1006/jmcc.1999.1090. 5. Schreieck J, Wang Y, Overbeck M, Schömig A, Schmitt C. Altered transient outward current in human atrial myocytes of patients with reduced left ventricular function. J Cardiovasc Electrophysiol. 2000;11(2):180-192. doi:10.1111/j.1540-8167.2000.tb00318.x. 6. Kebbati A. Hafid, Huang Cong Xin, Wang Xi, Zhao Qing Yan, Yang Bo. Difference between electrical remodelling after pulmonary veins and right atrium appendage pacing. EP Europace. 2007; 9(8): 608–612. 7. Lerman BB, Ellenbogen KA, Kadish A, et al. Electrophysiologic effects of a novel selective adenosine A1 agonist (CVT-510) on atrioventricular nodal conduction in humans. J Cardiovasc Pharmacol Ther. 2001;6(3):237-245. doi:10.1177/107424840100600304. 8. Workman AJ, Pau D, Redpath CJ, et al. Atrial cellular electrophysiological changes in patients with ventricular dysfunction may predispose to AF. Heart Rhythm. 2009;6(4):445-451. doi:10.1016/j.hrthm.2008.12.028. 9. Hirose M, Laurita KR. Calcium-mediated triggered activity is an underlying cellular mechanism of ectopy originating from the pulmonary vein in dogs. Am J Physiol Heart Circ Physiol. 2007;292(4):H1861-H1867. doi:10.1152/ajpheart.00826.2006. 10. Avula UMR, Hernandez JJ, Yamazaki M, Valdivia CR, Chu A, Rojas-Pena A, Kaur K, Ramos-Mondragón R, Anumonwo JM, Nattel S, Valdivia HH, Kalifa J. Atrial Infarction-Induced Spontaneous Focal Discharges and Atrial Fibrillation in Sheep: Role of Dantrolene-Sensitive Aberrant Ryanodine Receptor Calcium Release. Circ Arrhythm Electrophysiol. 2018 Mar;11(3):e005659. doi: 10.1161/CIRCEP.117.005659. 11. Yan J, Zhao W, Thomson JK, et al. Stress Signaling JNK2 Crosstalk With CaMKII Underlies Enhanced Atrial Arrhythmogenesis. Circ Res. 2018;122(6):821-835. doi:10.1161/CIRCRESAHA.117.312536. 12. Lin Y, Yang B, Garcia FC, et al. Comparison of left atrial electrophysiologic abnormalities during sinus rhythm in patients with different type of atrial fibrillation. J Interv Card Electrophysiol. 2014;39(1):57-67. doi:10.1007/s10840-013-9838-y. 13. O'Connell RP, Musa H, Gomez MS, et al. Free Fatty Acid Effects on the Atrial Myocardium: Membrane Ionic Currents Are Remodeled by the Disruption of T-Tubular Architecture. PLoS One. 2015;10(8):e0133052. Published 2015 Aug 14. doi:10.1371/journal.pone.0133052. 14. Honjo H, Boyett MR, Niwa R, et al. Pacing-induced spontaneous activity in myocardial sleeves of pulmonary veins after treatment with ryanodine. Circulation. 2003;107(14):1937-1943. doi:10.1161/01.CIR.0000062645.38670.BD. 15. Rietdorf K, Bootman MD, Sanderson MJ. Spontaneous, pro-arrhythmic calcium signals disrupt electrical pacing in mouse pulmonary vein sleeve cells. PLoS One. 2014;9(2):e88649. Published 2014 Feb 20. doi:10.1371/journal.pone.0088649. 16. Nakagawa H, Scherlag BJ, Patterson E, Ikeda A, Lockwood D, Jackman WM. Pathophysiologic basis of autonomic ganglionated plexus ablation in patients with atrial fibrillation. Heart Rhythm. 2009;6(12 Suppl):S26-S34. doi:10.1016/j.hrthm.2009.07.029. 17. Bukowska A, Schild L, Keilhoff G, et al. Mitochondrial dysfunction and redox signaling in atrial tachyarrhythmia. Exp Biol Med (Maywood). 2008;233(5):558-574. doi:10.3181/0706-RM-155. 18. Numata A, Miyauchi Y, Ono N, et al. Spontaneous atrial fibrillation initiated by tyramine in canine atria with increased sympathetic nerve sprouting. J Cardiovasc Electrophysiol. 2012;23(4):415-422. doi:10.1111/j.1540-8167.2011.02197.x. 19. Choi EK, Chang PC, Lee YS, et al. Triggered firing and atrial fibrillation in transgenic mice with selective atrial fibrosis induced by overexpression of TGF-β1. Circ J. 2012;76(6):1354-1362. doi:10.1253/circj.cj-11-1301. 20. Kuijpers NH, Potse M, van Dam PM, et al. Mechanoelectrical coupling enhances initiation and affects perpetuation of atrial fibrillation during acute atrial dilation. Heart Rhythm. 2011;8(3):429-436. doi:10.1016/j.hrthm.2010.11.020. 21. Suita K, Fujita T, Hasegawa N, et al. Norepinephrine-Induced Adrenergic Activation Strikingly Increased the Atrial Fibrillation Duration through β1- and α1-Adrenergic Receptor-Mediated Signaling in Mice. PLoS One. 2015;10(7):e0133664. Published 2015 Jul 23. doi:10.1371/journal.pone.0133664. 22. Prajapati R, Fujita T, Suita K, et al. Usefulness of Exchanged Protein Directly Activated by cAMP (Epac)1-Inhibiting Therapy for Prevention of Atrial and Ventricular Arrhythmias in Mice. Circ J. 2019;83(2):295-303. doi:10.1253/circj.CJ-18-0743. 23. Zhao ZH, Zhang HC, Xu Y, et al. Inositol-1,4,5-trisphosphate and ryanodine-dependent Ca2+ signaling in a chronic dog model of atrial fibrillation. Cardiology. 2007;107(4):269-276. doi:10.1159/000095517. | |
| **101 articles included atrial fibrillation but did not mention about experimental mapping of calcium waves, transients and/or sparks:**   1. Canpolat U, Aytemir K, Özer N, Oto A. The impact of cryoballoon-based catheter ablation on left atrial structural and potential electrical remodeling in patients with paroxysmal atrial fibrillation. J Interv Card Electrophysiol. 2015;44(2):131-139. doi:10.1007/s10840-015-0041-1. 2. Hori Y, Nakahara S, Anjo N, et al. Investigation of the atrial conduction time measured by tissue Doppler imaging at the left atrial appendage and the actual electrical conduction time: consideration of left atrial remodeling in atrial fibrillation patients. J Interv Card Electrophysiol. 2017;48(1):89-97. doi:10.1007/s10840-016-0185-7. 3. Chung CC, Lin YK, Chen YC, Kao YH, Yeh YH, Chen YJ. Factor Xa inhibition by rivaroxaban regulates fibrogenesis in human atrial fibroblasts with modulation of nitric oxide synthesis and calcium homeostasis. J Mol Cell Cardiol. 2018;123:128-138. doi:10.1016/j.yjmcc.2018.09.003. 4. Tingting H, Guangzhong L, Yanxiang Z, DongDong Y, Li S, Li W. Qiliqiangxin attenuates atrial structural remodeling in prolonged pacing-induced atrial fibrillation in rabbits. Naunyn Schmiedebergs Arch Pharmacol. 2019;392(5):585-592. doi:10.1007/s00210-018-01611-0. 5. Niwano S, Fukaya H, Sasaki T, Hatakeyama Y, Fujiki A, Izumi T. Effect of oral L-type calcium channel blocker on repetitive paroxysmal atrial fibrillation: spectral analysis of fibrillation waves in the Holter monitoring. Europace. 2007;9(12):1209-1215. doi:10.1093/europace/eum199. 6. Aksan G, Nar G, Soylu K, et al. Assessment of atrial electromechanical delay and left atrial mechanical functions in patients with psoriasis vulgaris. Echocardiography. 2015;32(4):615-622. doi:10.1111/echo.12706. 7. Páll A, Czifra Á, Sebestyén V, et al. Hemodiafiltration and hemodialysis differently affect P wave duration and dispersion on the surface electrocardiogram. Int Urol Nephrol. 2016;48(2):271-277. doi:10.1007/s11255-015-1144-4. 8. Tezcan UK, Amasyali B, Can I, et al. Increased P wave dispersion and maximum P wave duration after hemodialysis. Ann Noninvasive Electrocardiol. 2004;9(1):34-38. doi:10.1111/j.1542-474x.2004.91529.x. 9. Rago A, Russo V, Papa AA, et al. The role of the atrial electromechanical delay in predicting atrial fibrillation in beta-thalassemia major patients. J Interv Card Electrophysiol. 2017;48(2):147-157. doi:10.1007/s10840-016-0201-y. 10. Xi Q, Sahakian AV, Frohlich TG, Ng J, Swiryn S. Relationship between pattern of occurrence of atrial fibrillation and surface electrocardiographic fibrillatory wave characteristics. Heart Rhythm. 2004;1(6):656-663. doi:10.1016/j.hrthm.2004.09.010. 11. Phung TN, Moyer CB, Norton PT, Ferguson JD, Holmes JW. Effect of ablation pattern on mechanical function in the atrium. Pacing Clin Electrophysiol. 2017;40(6):648-654. doi:10.1111/pace.13086. 12. Barth AS, Merk S, Arnoldi E, et al. Reprogramming of the human atrial transcriptome in permanent atrial fibrillation: expression of a ventricular-like genomic signature. Circ Res. 2005;96(9):1022-1029. doi:10.1161/01.RES.0000165480.82737.33. 13. Nar G, Ergul B, Aksan G, Inci S. Assessment of Atrial Electromechanical Delay and Left Atrial Mechanical Functions in Patients with Ulcerative Colitis. Echocardiography. 2016;33(7):970-976. doi:10.1111/echo.13213. 14. Ozportakal H, Ozkok A, Alkan O, et al. Hemodialysis-induced repolarization abnormalities on ECG are influenced by serum calcium levels and ultrafiltration volumes. Int Urol Nephrol. 2017;49(3):509-515. doi: 10.1007/s11255-016-1472-z. 15. Akyel A, Oksüz F, Karadeniz M, et al. Atrial electromechanical delay in type 2 diabetes mellitus. Wien Klin Wochenschr. 2014;126(3-4):101-105. doi: 10.1007/s00508-013-0477-1. 16. You MJ, Langfield P, Campanari L, Dobbs M, Shrier A, Glass L. Demonstration of cardiac rotor and source mapping techniques in embryonic chick monolayers. Chaos. 2017;27(9):093938. doi:10.1063/1.5001459. 17. Aoyama Y, Niwano S, Niwano H, et al. Repetitive evaluation of fibrillation cycle length predicts the efficacy of bepridil for interruption of long-lasting persistent atrial fibrillation. Int Heart J. 2011;52(6):353-358. doi:10.1536/ihj.52.353. 18. Nomura M, Kawano T, Nakayasu K, Nakaya Y. The effects of losartan on signal-averaged P wave in patients with atrial fibrillation. Int J Cardiol. 2008;126(1):21-27. doi:10.1016/j.ijcard.2007.03.106. 19. Huang F, Huang JP, Pan JY, Bai ZL, Tang L, Zhou SH. Intracellular heat shock protein 70: a possible therapeutic target for preventing postoperative atrial fibrillation. Pharmazie. 2012;67(9):747-755. 20. Pérez Riera AR, Paixão-Almeida A, Barbosa-Barros R, et al. Congenital short QT syndrome: landmarks of the newest arrhythmogenic cardiac channelopathy. Cardiol J. 2013;20(5):464-471. doi:10.5603/CJ.a2013.0052. 21. Chang MG, Sato D, de Lange E, et al. Bi-stable wave propagation and early afterdepolarization-mediated cardiac arrhythmias. Heart Rhythm. 2012;9(1):115-122. doi:10.1016/j.hrthm.2011.08.014. 22. Hannes W, Fasol R, Zajonc H, et al. Diltiazem provides anti-ischemic and anti-arrhythmic protection in patients undergoing coronary bypass grafting. Eur J Cardiothorac Surg. 1993;7(5):239-245. doi: 10.1016/1010-7940(93)90211-s. 23. Decher N, Uyguner O, Scherer CR, et al. hKChIP2 is a functional modifier of hKv4.3 potassium channels: cloning and expression of a short hKChIP2 splice variant. Cardiovasc Res. 2001;52(2):255-264. doi:10.1016/s0008-6363(01)00374-1. 24. Seitelberger R, Hannes W, Gleichauf M, et al. Effects of diltiazem on perioperative ischemia, arrhythmias, and myocardial function in patients undergoing elective coronary bypass grafting. The Journal of Thoracic and Cardiovascular Surgery. 1994 Mar;107(3):811-821. 25. Gonsorcík J, Palko S, Takác M, et al. Holterovo monitorovanie EKG u chorých s chronickým zlyhaním obliciek v dlhodobom dialyzacnom programe [ECG Holter monitoring in patients with chronic renal failure in a long-term dialysis program]. Vnitr Lek. 1992;38(3):258-264. 26. Li X, Xue YM, Guo HM, et al. High hydrostatic pressure induces atrial electrical remodeling through upregulation of inflammatory cytokines. Life Sciences. 2020 Feb;242:117209. DOI: 10.1016/j.lfs.2019.117209. 27. Ni H, Adeniran I, Zhang H. In-silico investigations of the functional impact of KCNA5 mutations on atrial mechanical dynamics. Journal of Molecular and Cellular Cardiology. 2017 Oct;111:86-95. DOI: 10.1016/j.yjmcc.2017.08.005. 28. Morota S, Manolopoulos T, Eyjolfsson A, et al. Functional and pharmacological characteristics of permeability transition in isolated human heart mitochondria. PLoS One. 2013;8(6):e67747. Published 2013 Jun 28. doi:10.1371/journal.pone.0067747. 29. Leboeuf J, Basiez M, Massingham R. Electrophysiological effects of Org 7797 in the closed-chest anaesthetized dog. Br J Pharmacol. 1993;110(1):23-28. doi:10.1111/j.1476-5381.1993.tb13766.x. 30. Lu YY, Chen YC, Kao YH, Chen SA, Chen YJ. Extracellular matrix of collagen modulates arrhythmogenic activity of pulmonary veins through p38 MAPK activation. J Mol Cell Cardiol. 2013;59:159-166. doi:10.1016/j.yjmcc.2013.03.008. 31. Ritterman JB, Hossack KF, Bruce RA. Acute and chronic effects of diltiazem on A-V conduction at rest and during exercise. J Electrocardiol. 1982;15(1):41-46. doi:10.1016/s0022-0736(82)80043-5. 32. Guray U, Guray Y, Mecit B, Yilmaz MB, Sasmaz H, Korkmaz S. Maximum p wave duration and p wave dispersion in adult patients with secundum atrial septal defect: the impact of surgical repair. Ann Noninvasive Electrocardiol. 2004;9(2):136-141. doi:10.1111/j.1542-474X.2004.92532.x. 33. Ingemansson MP, Carlson J, Platonov P, Olsson SB. Effects of MgSO4 and glucose, insulin and potassium (GIK) on atrial conduction during the first 12 hours after DC-conversion of chronic atrial fibrillation. Scandinavian Cardiovascular Journal: SCJ. 2001 Oct;35(5):340-346. DOI: 10.1080/140174301317116325. 34. Oikawa J, Niwano S, Niwano H, et al. Prophylactic statin administration may prevent shortening of the fibrillation cycle length in patients with new-onset atrial fibrillation. Int Heart J. 2013;54(6):371-376. doi:10.1536/ihj.54.371. 35. Jafari Dehkordi A, Nasser Mohebi A, Heidari Soreshjani M. Frequency of cardiac arrhythmias in high- and low- yielding dairy cows. Vet Res Forum. 2014;5(1):1-5. 36. Etemad T, Hosseinsabet A. Determinants of left intra-atrial electromechanical delay as evaluated by tissue Doppler imaging in candidates for coronary artery bypass graft surgery. Echocardiography (Mount Kisco, N.Y.). 2020 Feb;37(2):260-269. DOI: 10.1111/echo.14599. 37. Büttner P, Ueberham L, Shoemaker MB, et al. Identification of Central Regulators of Calcium Signaling and ECM-Receptor Interaction Genetically Associated With the Progression and Recurrence of Atrial Fibrillation. Front Genet. 2018;9:162. Published 2018 May 16. doi:10.3389/fgene.2018.00162. 38. Cerit L, Kemal H, Gulsen K, Ozcem B, Cerit Z, Duygu H. Relationship between Vitamin D and the development of atrial fibrillation after on-pump coronary artery bypass graft surgery. Cardiovasc J Afr. 2017;28(2):104-107. doi:10.5830/CVJA-2016-064. 39. Pedersen KB, Madsen C, Sandgaard NCF, et al. Predictive Markers of Atrial Fibrillation in Patients with Transient Ischemic Attack. J Stroke Cerebrovasc Dis. 2020;29(4):104643. doi:10.1016/j.jstrokecerebrovasdis.2020.104643. 40. Tsadok MA, Jackevicius CA, Essebag V, et al. Rhythm versus rate control therapy and subsequent stroke or transient ischemic attack in patients with atrial fibrillation. Circulation. 2012;126(23):2680-2687. doi:10.1161/CIRCULATIONAHA.112.092494. 41. Wettwer E, Christ T, Endig S, et al. The new antiarrhythmic drug vernakalant: ex vivo study of human atrial tissue from sinus rhythm and chronic atrial fibrillation. Cardiovasc Res. 2013;98(1):145-154. doi:10.1093/cvr/cvt006. 42. Yao C, Veleva T, Scott L Jr, et al. Enhanced Cardiomyocyte NLRP3 Inflammasome Signaling Promotes Atrial Fibrillation [published correction appears in Circulation. 2019 Apr 23;139(17):e889]. Circulation. 2018;138(20):2227-2242. doi:10.1161/CIRCULATIONAHA.118.035202. 43. Liu Z, Finet JE, Wolfram JA, Anderson ME, Ai X, Donahue JK. Calcium/calmodulin-dependent protein kinase II causes atrial structural remodeling associated with atrial fibrillation and heart failure. Heart Rhythm. 2019;16(7):1080-1088. doi:10.1016/j.hrthm.2019.01.013. 44. Zou D, Geng N, Chen Y, et al. Ranolazine improves oxidative stress and mitochondrial function in the atrium of acetylcholine-CaCl2 induced atrial fibrillation rats. Life Sci. 2016;156:7-14. doi:10.1016/j.lfs.2016.05.026. 45. Sossalla S, Kallmeyer B, Wagner S, et al. Altered Na(+) currents in atrial fibrillation effects of ranolazine on arrhythmias and contractility in human atrial myocardium. J Am Coll Cardiol. 2010;55(21):2330-2342. doi:10.1016/j.jacc.2009.12.055. 46. Feola I, Volkers L, Majumder R, et al. Localized Optogenetic Targeting of Rotors in Atrial Cardiomyocyte Monolayers. Circ Arrhythm Electrophysiol. 2017;10(11):e005591. doi:10.1161/CIRCEP.117.005591. 47. Arora R, Aistrup GL, Supple S, et al. Regional distribution of T-tubule density in left and right atria in dogs. Heart Rhythm. 2017;14(2):273-281. doi:10.1016/j.hrthm.2016.09.022. 48. Cunha SR, Hund TJ, Hashemi S, et al. Defects in ankyrin-based membrane protein targeting pathways underlie atrial fibrillation. Circulation. 2011;124(11):1212-1222. doi:10.1161/CIRCULATIONAHA.111.023986. 49. Sharma AK, Kishore K, Sharma D, et al. Cardioprotective activity of alcoholic extract of Tinospora cordifolia (Willd.) Miers in calcium chloride-induced cardiac arrhythmia in rats. J Biomed Res. 2011;25(4):280-286. doi:10.1016/S1674-8301(11)60038-9. 50. Purohit A, Rokita AG, Guan X, et al. Oxidized Ca(2+)/calmodulin-dependent protein kinase II triggers atrial fibrillation. Circulation. 2013;128(16):1748-1757. doi:10.1161/CIRCULATIONAHA.113.003313. 51. Makara MA, Curran J, Little SC, et al. Ankyrin-G coordinates intercalated disc signaling platform to regulate cardiac excitability in vivo. Circ Res. 2014;115(11):929-938. doi:10.1161/CIRCRESAHA.115.305154. 52. Castro L, Mialet-Perez J, Guillemeau A, et al. Differential functional effects of two 5-HT4 receptor isoforms in adult cardiomyocytes. J Mol Cell Cardiol. 2005;39(2):335-344. doi:10.1016/j.yjmcc.2005.04.009. 53. Patel BB, Raad M, Sebag IA, Chalifour LE. Sex-specific cardiovascular responses to control or high fat diet feeding in C57bl/6 mice chronically exposed to bisphenol A. Toxicol Rep. 2015;2:1310-1318. Published 2015 Oct 1. doi:10.1016/j.toxrep.2015.09.008. 54. Molina CE, Abu-Taha IH, Wang Q, et al. Profibrotic, Electrical, and Calcium-Handling Remodeling of the Atria in Heart Failure Patients With and Without Atrial Fibrillation. Front Physiol. 2018;9:1383. Published 2018 Oct 9. doi:10.3389/fphys.2018.01383. 55. Hu X, Li J, van Marion DMS, Zhang D, Brundel BJJM. Heat shock protein inducer GGA*-59 reverses contractile and structural remodeling via restoration of the microtubule network in experimental Atrial Fibrillation. J Mol Cell Cardiol. 2019;134:86-97. doi:10.1016/j.yjmcc.2019.07.006. 56. Tsai CT, Chiang FT, Tseng CD, et al. Mechanical stretch of atrial myocyte monolayer decreases sarcoplasmic reticulum calcium adenosine triphosphatase expression and increases susceptibility to repolarization alternans. Journal of the American College of Cardiology. 2011 Nov;58(20):2106-2115. DOI: 10.1016/j.jacc.2011.07.039. 57. Chen WJ, Yeh YH, Lin KH, Chang GJ, Kuo CT. Molecular characterization of thyroid hormone-inhibited atrial L-type calcium channel expression: implication for atrial fibrillation in hyperthyroidism. Basic Res Cardiol. 2011;106(2):163-174. doi:10.1007/s00395-010-0149-5. 58. van Marion DM, Hu X, Zhang D, et al. Screening of novel HSP-inducing compounds to conserve cardiomyocyte function in experimental atrial fibrillation. Drug Des Devel Ther. 2019;13:345-364. Published 2019 Jan 18. doi:10.2147/DDDT.S176924. 59. Akar JG, Everett TH, Ho R, et al. Intracellular chloride accumulation and subcellular elemental distribution during atrial fibrillation. Circulation. 2003;107(13):1810-1815. doi:10.1161/01.CIR.0000058462.23347.93. 60. Thijssen VL, Ausma J, Gorza L, et al. Troponin I isoform expression in human and experimental atrial fibrillation. Circulation. 2004;110(7):770-775. doi:10.1161/01.CIR.0000138849.03311.C6. 61. Gong D, Zhang Y, Cai B, et al. Characterization and comparison of Na+, K+ and Ca2+ currents between myocytes from human atrial right appendage and atrial septum. Cell Physiol Biochem. 2008;21(5-6):385-394. doi:10.1159/000129631. 62. Oh S, Kim KB, Ahn H, Cho HJ, Choi YS. Remodeling of ion channel expression in patients with chronic atrial fibrillation and mitral valvular heart disease. Korean J Intern Med. 2010;25(4):377-385. doi:10.3904/kjim.2010.25.4.377. 63. Zhang YH, Sun HY, Chen KH, et al. Evidence for functional expression of TRPM7 channels in human atrial myocytes. Basic Res Cardiol. 2012;107(5):282. doi:10.1007/s00395-012-0282-4. 64. Cha TJ, Ehrlich JR, Zhang L, Nattel S. Atrial ionic remodeling induced by atrial tachycardia in the presence of congestive heart failure. Circulation. 2004;110(12):1520-1526. doi:10.1161/01.CIR.0000142052.03565.87. 65. Lin, Y., Lu, Y., Chen, Y. et al. Nitroprusside modulates pulmonary vein arrhythmogenic activity. J Biomed Sci 17, 20 (2010). doi:10.1186/1423-0127-17-20. 66. Watanabe H, Ma M, Washizuka T, et al. Thyroid hormone regulates mRNA expression and currents of ion channels in rat atrium. Biochem Biophys Res Commun. 2003;308(3):439-444. doi:10.1016/s0006-291x(03)01420-7. 67. Yu J, Li W, Li Y, et al. Activation of β(3)-adrenoceptor promotes rapid pacing-induced atrial electrical remodeling in rabbits. Cell Physiol Biochem. 2011;28(1):87-96. doi:10.1159/000331717. 68. Yue L, Melnyk P, Gaspo R, Wang Z, Nattel S. Molecular mechanisms underlying ionic remodeling in a dog model of atrial fibrillation. Circ Res. 1999;84(7):776-784. doi:10.1161/01.res.84.7.776. 69. Dun W, Ozgen N, Hirose M, et al. Ionic mechanisms underlying region-specific remodeling of rabbit atrial action potentials caused by intermittent burst stimulation. Heart Rhythm. 2007;4(4):499-507. doi:10.1016/j.hrthm.2006.12.032. 70. Chen YJ, Chen YC, Yeh HI, Lin CI, Chen SA. Electrophysiology and arrhythmogenic activity of single cardiomyocytes from canine superior vena cava. Circulation. 2002;105(22):2679-2685. doi:10.1161/01.cir.0000016822.96362.26. 71. Ehrlich JR, Cha TJ, Zhang L, et al. Cellular electrophysiology of canine pulmonary vein cardiomyocytes: action potential and ionic current properties. J Physiol. 2003;551(Pt 3):801-813. doi:10.1113/jphysiol.2003.046417. 72. Chen, YC. Chen, SA, Chen YJ, Tai CT, Chan P and Lin CI. T‐Type Calcium Current in Electrical Activity of Cardiomyocytes Isolated from Rabbit Pulmonary Vein. Journal of Cardiovascular Electrophysiology, 2014; 15: 567-571. doi:10.1046/j.1540-8167.2004.03399.x. 73. Shi L, Yang XC, Liu XL, Zong M, Wu YL. [Effects of ShenSongYangXin on action potential and some current channels in isolated rabbit pulmonary vein cardiomyocytes]. Zhonghua yi xue za zhi. 2009 Aug;89(30):2142-2146. 74. Cheng CC, Huang CF, Chen YC, et al. Heat-stress responses modulate beta-adrenergic agonist and angiotensin II effects on the arrhythmogenesis of pulmonary vein cardiomyocytes. J Cardiovasc Electrophysiol. 2011;22(2):183-190. doi:10.1111/j.1540-8167.2010.01849.x. 75. Laszlo R, Eick C, Schwiebert M, et al. Transient outward potassium current in rabbit atrium is depressed after short-time rapid atrial pacing but recovers after a longer pacing period. Gen Physiol Biophys. 2008;27(3):174-178. 76. Laszlo R, Bentz K, Konior A, et al. Effects of selective mineralocorticoid receptor antagonism on atrial ion currents and early ionic tachycardia-induced electrical remodelling in rabbits. Naunyn Schmiedebergs Arch Pharmacol. 2010;382(4):347-356. doi:10.1007/s00210-010-0553-2. 77. Huang SY, Lu YY, Chen YC, et al. Hydrogen Peroxide Modulates Electrophysiological Characteristics of Left Atrial Myocytes. Acta Cardiol Sin. 2014;30(1):38-45. 78. Chang CJ, Chen YC, Lin YK, Huang JH, Chen SA, Chen YJ. Rivaroxaban modulates electrical and mechanical characteristics of left atrium. J Biomed Sci. 2013;20(1):17. Published 2013 Mar 15. doi:10.1186/1423-0127-20-17. 79. Workman AJ, Kane KA, Russell JA, Norrie J, Rankin AC. Chronic beta-adrenoceptor blockade and human atrial cell electrophysiology: evidence of pharmacological remodelling. Cardiovasc Res. 2003;58(3):518-525. doi:10.1016/s0008-6363(03)00263-3. 80. Chen YJ, Chen YC, Tai CT, Yeh HI, Lin CI, Chen SA. Angiotensin II and angiotensin II receptor blocker modulate the arrhythmogenic activity of pulmonary veins. Br J Pharmacol. 2006;147(1):12-22. doi:10.1038/sj.bjp.0706445. 81. Udyavar AR, Chen YC, Chen YJ, Cheng CC, Lin CI, Chen SA. Endothelin-1 modulates the arrhythmogenic activity of pulmonary veins. J Cardiovasc Electrophysiol. 2008;19(3):285-292. doi:10.1111/j.1540-8167.2007.01033.x. 82. Laszlo R, Eick C, Rueb N, et al. Inhibition of the renin-angiotensin system: effects on tachycardia-induced early electrical remodelling in rabbit atrium. J Renin Angiotensin Aldosterone Syst. 2008;9(3):125-132. doi:10.1177/1470320308095262. 83. Cheng W, Zhu Y, Wang H. The MAPK pathway is involved in the regulation of rapid pacing-induced ionic channel remodeling in rat atrial myocytes. Mol Med Rep. 2016;13(3):2677-2682. doi:10.3892/mmr.2016.4862. 84. Suita K, Fujita T, Cai W, et al. Vidarabine, an anti-herpesvirus agent, prevents catecholamine-induced arrhythmias without adverse effect on heart function in mice. Pflugers Arch. 2018;470(6):923-935. doi:10.1007/s00424-018-2121-4. 85. Tobelaim WS, Dvir M, Lebel G, et al. Competition of calcified calmodulin N lobe and PIP2 to an LQT mutation site in Kv7.1 channel. Proc Natl Acad Sci U S A. 2017;114(5):E869-E878. doi:10.1073/pnas.1612622114. 86. Aistrup GL, Villuendas R, Ng J, et al. Targeted G-protein inhibition as a novel approach to decrease vagal atrial fibrillation by selective parasympathetic attenuation. Cardiovasc Res. 2009;83(3):481-492. doi:10.1093/cvr/cvp148. 87. Xiao Z, Guo W, Sun B, et al. Enhanced Cytosolic Ca2+ Activation Underlies a Common Defect of Central Domain Cardiac Ryanodine Receptor Mutations Linked to Arrhythmias. J Biol Chem. 2016;291(47):24528-24537. doi:10.1074/jbc.M116.756528. 88. Qi XY, Yeh YH, Xiao L, et al. Cellular signaling underlying atrial tachycardia remodeling of L-type calcium current. Circ Res. 2008;103(8):845-854. doi:10.1161/CIRCRESAHA.108.175463. 89. Ke L, Meijering RA, Hoogstra-Berends F, et al. HSPB1, HSPB6, HSPB7 and HSPB8 protect against RhoA GTPase-induced remodeling in tachypaced atrial myocytes. PLoS One. 2011;6(6):e20395. doi:10.1371/journal.pone.0020395. 90. Huang Y, Lu CY, Yan W, Gao L, Chen Q, Zhang YJ. [Influence of cyclosporine A on atrial L-type calcium channel alpha1c subunit in a canine model of atrial fibrillation]. Zhonghua Xin Xue Guan Bing Za Zhi. 2009;37(2):112-4. Chinese. PMID: 19719984. 91. Baczko I, Liknes D, Yang W, et al. Characterization of a novel multifunctional resveratrol derivative for the treatment of atrial fibrillation. Br J Pharmacol. 2014;171(1):92-106. doi:10.1111/bph.12409. 92. Lin YK, Chen YC, Chen YA, Yeh YH, Chen SA, Chen YJ. B-Type Natriuretic Peptide Modulates Pulmonary Vein Arrhythmogenesis: A Novel Potential Contributor to the Genesis of Atrial Tachyarrhythmia in Heart Failure. J Cardiovasc Electrophysiol. 2016;27(12):1462-1471. doi:10.1111/jce.13093. 93. Farkasfalvi K, Stagg MA, Coppen SR, et al. Direct effects of apelin on cardiomyocyte contractility and electrophysiology. Biochem Biophys Res Commun. 2007;357(4):889-895. doi:10.1016/j.bbrc.2007.04.017. 94. Hou JW, Li W, Fei YD, et al. ICaL and Ito mediate rate-dependent repolarization in rabbit atrial myocytes. J Physiol Biochem. 2018;74(1):57-67. doi:10.1007/s13105-017-0603-z. 95. Salari S, Silverå Ejneby M, Brask J, Elinder F. Isopimaric acid - a multi-targeting ion channel modulator reducing excitability and arrhythmicity in a spontaneously beating mouse atrial cell line. Acta Physiol (Oxf). 2018;222(1):10.1111/apha.12895. doi:10.1111/apha.12895. 96. Molina CE, Llach A, Herraiz-Martínez A, et al. Prevention of adenosine A2A receptor activation diminishes beat-to-beat alternation in human atrial myocytes. Basic Res Cardiol. 2016;111(1):5. doi:10.1007/s00395-015-0525-2. 97. Laszlo R, Menzel KA, Bentz K, et al. Atorvastatin treatment affects atrial ion currents and their tachycardia-induced remodeling in rabbits. Life Sci. 2010;87(15-16):507-513. doi:10.1016/j.lfs.2010.09.010. 98. Wang T, Huang CX, Jiang H, Tang QZ, Yang B, Li GS. [Characteristics of electrophysiology and effects of ouabain on transient outward potassium current and L-type calcium current of left atrium posterior wall in rabbits]. Zhonghua Yi Xue Za Zhi. 2009;89(44). PMID: 20193278. 99. Yue L, Feng J, Gaspo R, Li GR, Wang Z, Nattel S. Ionic remodeling underlying action potential changes in a canine model of atrial fibrillation. Circ Res. 1997;81(4):512-525. doi:10.1161/01.res.81.4.512. 100. Chen PS, Chen LS, Fishbein MC, Lin SF, Nattel S. Role of the autonomic nervous system in atrial fibrillation: pathophysiology and therapy. Circ Res. 2014;114(9):1500-1515. doi:10.1161/CIRCRESAHA.114.303772. 101. Barana A, Matamoros M, Dolz-Gaitón P, et al. Chronic atrial fibrillation increases microRNA-21 in human atrial myocytes decreasing L-type calcium current. Circ Arrhythm Electrophysiol. 2014;7(5):861-868. doi:10.1161/CIRCEP.114.001709. | |
| **27 articles reported on computational/mathematical models (i.e. non-experimental):**   1. Zile MA, Trayanova NA. Increased thin filament activation enhances alternans in human chronic atrial fibrillation. Am J Physiol Heart Circ Physiol. 2018;315(5):H1453-H1462. doi:10.1152/ajpheart.00658.2017 2. Chang KC, Bayer JD, Trayanova NA. Disrupted calcium release as a mechanism for atrial alternans associated with human atrial fibrillation. PLoS Comput Biol. 2014;10(12):e1004011. Published 2014 Dec 11. doi:10.1371/journal.pcbi.1004011. 3. Sutanto H, van Sloun B, Schönleitner P, van Zandvoort MAMJ, Antoons G, Heijman J. The Subcellular Distribution of Ryanodine Receptors and L-Type Ca2+ Channels Modulates Ca2+-Transient Properties and Spontaneous Ca2+-Release Events in Atrial Cardiomyocytes. Front Physiol. 2018;9:1108. Published 2018 Aug 14. doi:10.3389/fphys.2018.01108. 4. Yoo S, Aistrup G, Shiferaw Y, et al. Oxidative stress creates a unique, CaMKII-mediated substrate for atrial fibrillation in heart failure. JCI Insight. 2018;3(21):e120728. Published 2018 Nov 2. doi:10.1172/jci.insight.120728. 5. Koivumäki JT, Seemann G, Maleckar MM, Tavi P. In silico screening of the key cellular remodeling targets in chronic atrial fibrillation. PLoS Comput Biol. 2014;10(5):e1003620. Published 2014 May 22. doi:10.1371/journal.pcbi.1003620. 6. Morotti S, McCulloch AD, Bers DM, Edwards AG, Grandi E. Atrial-selective targeting of arrhythmogenic phase-3 early afterdepolarizations in human myocytes. J Mol Cell Cardiol. 2016;96:63-71. doi:10.1016/j.yjmcc.2015.07.030. 7. Tsai CT, Chiang FT, Tseng CD, et al. Increased expression of mineralocorticoid receptor in human atrial fibrillation and a cellular model of atrial fibrillation. J Am Coll Cardiol. 2010;55(8):758-770. doi:10.1016/j.jacc.2009.09.045. 8. Lee YS, Hwang M, Song JS, et al. The Contribution of Ionic Currents to Rate-Dependent Action Potential Duration and Pattern of Reentry in a Mathematical Model of Human Atrial Fibrillation. PLoS One. 2016;11(3):e0150779. doi:10.1371/journal.pone.0150779. 9. Severi S, Pogliani D, Fantini G, et al. Alterations of atrial electrophysiology induced by electrolyte variations: combined computational and P-wave analysis. Europace. 2010;12(6):842-849. doi:10.1093/europace/euq042 10. Cherry EM, Evans SJ. Properties of two human atrial cell models in tissue: restitution, memory, propagation, and reentry. J Theor Biol. 2008;254(3):674-690. doi:10.1016/j.jtbi.2008.06.030. 11. Grandi E, Pandit SV, Voigt N, et al. Human atrial action potential and Ca2+ model: sinus rhythm and chronic atrial fibrillation. Circ Res. 2011;109(9):1055-1066. doi:10.1161/CIRCRESAHA.111.253955. 12. Adeniran I, MacIver DH, Garratt CJ, Ye J, Hancox JC, Zhang H. Effects of Persistent Atrial Fibrillation-Induced Electrical Remodeling on Atrial Electro-Mechanics - Insights from a 3D Model of the Human Atria. PLoS One. 2015;10(11):e0142397. doi:10.1371/journal.pone.0142397. 13. Jeong DU, Lim KM. Influence of the KCNQ1 S140G Mutation on Human Ventricular Arrhythmogenesis and Pumping Performance: Simulation Study. Front Physiol. 2018;9:926. Published 2018 Jul 31. doi:10.3389/fphys.2018.00926. 14. Courtemanche M, Ramirez RJ, Nattel S. Ionic mechanisms underlying human atrial action potential properties: insights from a mathematical model. Am J Physiol. 1998;275(1):H301-H321. doi:10.1152/ajpheart.1998.275.1.H301. 15. Pandit SV, Berenfeld O, Anumonwo JM, et al. Ionic determinants of functional reentry in a 2-D model of human atrial cells during simulated chronic atrial fibrillation. Biophys J. 2005;88(6):3806-3821. doi:10.1529/biophysj.105.060459. 16. Wolf RM, Glynn P, Hashemi S, et al. Atrial fibrillation and sinus node dysfunction in human ankyrin-B syndrome: a computational analysis. Am J Physiol Heart Circ Physiol. 2013;304(9):H1253-H1266. doi:10.1152/ajpheart.00734.2012. 17. Grégoire-Lacoste F, Jacquemet V, Vinet A. Bifurcations, sustained oscillations and torus bursting involving ionic concentrations dynamics in a canine atrial cell model. Math Biosci. 2014;250:10-25. doi:10.1016/j.mbs.2014.01.010. 18. Shy D, Gillet L, Abriel H. Cardiac sodium channel NaV1.5 distribution in myocytes via interacting proteins: the multiple pool model. Biochim Biophys Acta. 2013;1833(4):886-894. doi:10.1016/j.bbamcr.2012.10.026. 19. Courtemanche M, Ramirez RJ, Nattel S. Ionic targets for drug therapy and atrial fibrillation-induced electrical remodeling: insights from a mathematical model. Cardiovasc Res. 1999;42(2):477-489. doi:10.1016/s0008-6363(99)00034-6. 20. Redpath CJ, Bou Khalil M, Drozdzal G, Radisic M, McBride HM. Mitochondrial hyperfusion during oxidative stress is coupled to a dysregulation in calcium handling within a C2C12 cell model. PLoS One. 2013;8(7):e69165. Published 2013 Jul 8. doi:10.1371/journal.pone.0069165. 21. Walmsley J, Rodriguez JF, Mirams GR, Burrage K, Efimov IR, Rodriguez B. mRNA expression levels in failing human hearts predict cellular electrophysiological remodeling: a population-based simulation study. PLoS One. 2013;8(2):e56359. doi:10.1371/journal.pone.0056359. 22. Ashihara T, Namba T, Ito M, Kinoshita M, Nakazawa K. The dynamics of vortex-like reentry wave filaments in three-dimensional computer models. J Electrocardiol. 1999;32 Suppl:129-138. doi:10.1016/s0022-0736(99)90062-6. 23. Kerckhoffs RC, Omens JH, McCulloch AD. Mechanical discoordination increases continuously after the onset of left bundle branch block despite constant electrical dyssynchrony in a computational model of cardiac electromechanics and growth. Europace. 2012;14 Suppl 5(Suppl 5):v65-v72. doi:10.1093/europace/eus274. 24. Schild L, Bukowska A, Gardemann A, et al. Rapid pacing of embryoid bodies impairs mitochondrial ATP synthesis by a calcium-dependent mechanism--a model of in vitro differentiated cardiomyocytes to study molecular effects of tachycardia. Biochim Biophys Acta. 2006;1762(6):608-615. doi:10.1016/j.bbadis.2006.03.005. 25. Onal B, Gratz D, Hund TJ. Ca2+/calmodulin-dependent kinase II-dependent regulation of atrial myocyte late Na+ current, Ca2+ cycling, and excitability: a mathematical modeling study. Am J Physiol Heart Circ Physiol. 2017;313(6):H1227-H1239. doi:10.1152/ajpheart.00185.2017. 26. Shuen-Hsin Liu, Ya-Wen Hsiao, Eric Chong, et. al. Rhodiola Inhibits Atrial Arrhythmogenesis in a Heart Failure Model. Journal of Cardiovascualr Electrophysiology. 2016; 27(9): 1093-1101. doi: 10.1111/jce.13026. 27. Lim, B., Kim, J., Hwang, M. et al. In situ procedure for high-efficiency computational modeling of atrial fibrillation reflecting personal anatomy, fiber orientation, fibrosis, and electrophysiology. Sci Rep. 2020;10: 2417. | |
| **23 articles were controlled trials or case reports:**   1. Yan L, Jiang T, Yang X, Xu M. Spontaneous conversion of atrial fibrillation caused by severe hyperkalemia: A case report. Medicine (Baltimore). 2018;97(15):e0442. doi:10.1097/MD.0000000000010442. 2. Hoshi Y, Nozawa Y, Ogasawara M, et al. Atrial electromechanical interval may predict cardioembolic stroke in apparently low risk elderly patients with paroxysmal atrial fibrillation. Echocardiography. 2014;31(2):140-148. doi:10.1111/echo.12329. 3. Viland J, Langørgen J, Wendelbo Ø. A somnolent woman in her fifties with acute circulatory failure. Tidsskrift for den Norske Laegeforening: Tidsskrift for Praktisk Medicin, ny Raekke. 2019;139(9). DOI: 10.4045/tidsskr.18.0683. 4. De Vriese AS, Caluwé R, Pyfferoen L, et al. Multicenter Randomized Controlled Trial of Vitamin K Antagonist Replacement by Rivaroxaban with or without Vitamin K2 in Hemodialysis Patients with Atrial Fibrillation: the Valkyrie Study. J Am Soc Nephrol. 2020;31(1):186-196. doi:10.1681/ASN.2019060579. 5. Cleland JG, Coletta AP, Abdellah AT, Cullington D, Clark AL, Rigby AS. Clinical trials update from the American Heart Association 2007: CORONA, RethinQ, MASCOT, AF-CHF, HART, MASTER, POISE and stem cell therapy. Eur J Heart Fail. 2008;10(1):102-108. doi:10.1016/j.ejheart.2007.12.004. 6. Houltz B, Darpö B, Swedberg K, et al. Effects of the Ikr-blocker almokalant and predictors of conversion of chronic atrial tachyarrhythmias to sinus rhythm. A prospective study. Cardiovasc Drugs Ther. 1999;13(4):329-338. doi:10.1023/a:1007759500274. 7. Heller DA, Ahern FM, Kozak M. Changes in rates of beta-blocker use between 1994 and 1997 among elderly survivors of acute myocardial infarction. Am Heart J. 2000;140(4):663-671. doi:10.1067/mhj.2000.109650. 8. Suárez G, Herrera M, Vera A, Torrado E, Férriz J, Arboleda JA. Prediction on admission of in-hospital mortality in patients older than 70 years with acute myocardial infarction. Chest. 1995;108(1):83-88. doi:10.1378/chest.108.1.83. 9. Proietti M, Raparelli V, Laroche C, et al. Adverse outcomes in patients with atrial fibrillation and peripheral arterial disease: a report from the EURObservational research programme pilot survey on atrial fibrillation. Europace. 2017;19(9):1439-1448. doi:10.1093/europace/euw169. 10. Corley SD, Epstein AE, DiMarco JP, et al. Relationships between sinus rhythm, treatment, and survival in the Atrial Fibrillation Follow-Up Investigation of Rhythm Management (AFFIRM) Study. Circulation. 2004;109(12):1509-1513. doi:10.1161/01.CIR.0000121736.16643.11. 11. Hinton W, McGovern A, Coyle R, et al. Incidence and prevalence of cardiovascular disease in English primary care: a cross-sectional and follow-up study of the Royal College of General Practitioners (RCGP) Research and Surveillance Centre (RSC). BMJ Open. 2018;8(8):e020282. doi:10.1136/bmjopen-2017-020282. 12. Shuey MM, Gandelman JS, Chung CP, et al. Characteristics and treatment of African-American and European-American patients with resistant hypertension identified using the electronic health record in an academic health centre: a case-control study. BMJ Open. 2018;8(6):e021640. Published 2018 Jun 27. doi:10.1136/bmjopen-2018-021640. 13. Meinertz T, Kirch W, Rosin L, et al. Management of atrial fibrillation by primary care physicians in Germany: baseline results of the ATRIUM registry. Clin Res Cardiol. 2011;100(10):897-905. doi:10.1007/s00392-011-0320-5. 14. Fu S, Hirte H, Welch S, et al. First-in-human phase I study of SOR-C13, a TRPV6 calcium channel inhibitor, in patients with advanced solid tumors Invest New Drugs. 2017;35(3):324-333. doi:10.1007/s10637-017-0438-z. 15. Gibson AO, Blaha MJ, Arnan MK, et al. Coronary artery calcium and incident cerebrovascular events in an asymptomatic cohort. The MESA Study. JACC Cardiovasc Imaging. 2014;7(11):1108-1115. doi:10.1016/j.jcmg.2014.07.009. 16. Hypothyroidism in adults. Levothyroxine if warranted by clinical and laboratory findings, not for simple TSH elevation. Prescrire Int. 2015;24(164):241-4, 246. PMID: 26594730. 17. Umemoto S, Ogihara T, Rakugi H, et al. Effects of a benidipine-based combination therapy on the risk of stroke according to stroke subtype: the COPE trial. Hypertens Res. 2013;36(12):1088-1095. doi:10.1038/hr.2013.100. 18. Toivonen L, Raatikainen P, Walfridsson H, et al. A randomized invasive cardiac electrophysiology study of the combined ion channel blocker AZD1305 in patients after catheter ablation of atrial flutter. J Cardiovasc Pharmacol. 2010;56(3):300-308. doi:10.1097/FJC.0b013e3181ed0859. 19. Podesser BK, Schwarzacher S, Zwoelfer W, Binder TM, Wolner E, Seitelberger R. Comparison of perioperative myocardial protection with nifedipine versus nifedipine and metoprolol in patients undergoing elective coronary artery bypass grafting. J Thorac Cardiovasc Surg. 1995;110(5):1461-1469. doi:10.1016/S0022-5223(95)70069-2. 20. Avezum A, Makdisse M, Spencer F, et al. Impact of age on management and outcome of acute coronary syndrome: observations from the Global Registry of Acute Coronary Events (GRACE). Am Heart J. 2005;149(1):67-73. doi:10.1016/j.ahj.2004.06.003. 21. Kishima H, Mine T, Fukuhara E, Ashida K, Ishihara M. Predictors of left atrial appendage stunning after electrical cardioversion in patients with atrial fibrillation. Int J Cardiovasc Imaging. 2019;35(9):1549-1555. doi:10.1007/s10554-019-01592-y. 22. Cho Y, Jeong W, Kim S, et al. Serial measurement of glyphosate blood concentration in a glyphosate potassium herbicide-intoxicated patient: A case report. Am J Emerg Med. 2019;37(8):1600.e5-1600.e6. doi:10.1016/j.ajem.2019.04.042 23. Yujing W, Congxin H, Shaning Y, et al. Digitalis does not improve left atrial mechanical dysfunction after successful electrical cardioversion of chronic atrial fibrillation. Cell Biochem Biophys. 2010;57(1):27-34. doi:10.1007/s12013-010-9080-5. | |
| **13 articles only included signaling pathway(s) but not calcium waves, transients and/or sparks:**   1. Du J, Xie J, Zhang Z, et al. TRPM7-mediated Ca2+ signals confer fibrogenesis in human atrial fibrillation. Circ Res. 2010;106(5):992-1003. doi:10.1161/CIRCRESAHA.109.206771. 2. Li X, Zima AV, Sheikh F, Blatter LA, Chen J. Endothelin-1-induced arrhythmogenic Ca2+ signaling is abolished in atrial myocytes of inositol-1,4,5-trisphosphate(IP3)-receptor type 2-deficient mice. Circ Res. 2005;96(12):1274-1281. doi:10.1161/01.RES.0000172556.05576.4c. 3. Liang X, Xie H, Zhu PH, et al. Enhanced activity of inositol-1,4,5-trisphosphate receptors in atrial myocytes of atrial fibrillation patients. Cardiology. 2009;114(3):180-191. doi:10.1159/000228584 4. Wiersma M, Meijering RAM, Qi XY, et al. Endoplasmic Reticulum Stress Is Associated With Autophagy and Cardiomyocyte Remodeling in Experimental and Human Atrial Fibrillation. J Am Heart Assoc. 2017;6(10):e006458. Published 2017 Oct 24. doi:10.1161/JAHA.117.006458. 5. DeSantiago J, Bare DJ, Varma D, Solaro RJ, Arora R, Banach K. Loss of p21-activated kinase 1 (Pak1) promotes atrial arrhythmic activity. Heart Rhythm. 2018;15(8):1233-1241. doi:10.1016/j.hrthm.2018.03.041. 6. Liu CH, Hua N, Fu X, Pan YL, Li B, Li XD. Metformin regulates atrial SK2 and SK3 expression through inhibiting the PKC/ERK signaling pathway in type 2 diabetic rats. BMC Cardiovasc Disord. 2018;18(1):236. Published 2018 Dec 13. doi:10.1186/s12872-018-0950-x. 7. Sun J, Ailiman M. Regulation of calcium pump through Notch/Jagged/Hes signaling pathway in canine model of chronic atrial fibrillation. Int J Clin Exp Pathol. 2019;12(11):4034-4040. Published 2019 Nov 1. 8. Bukowska A, Lendeckel U, Hirte D, et al. Activation of the calcineurin signaling pathway induces atrial hypertrophy during atrial fibrillation. Cell Mol Life Sci. 2006;63(3):333-342. doi:10.1007/s00018-005-5353-3. 9. Yeh YH, Kuo CT, Chang GJ, et al. Rosuvastatin suppresses atrial tachycardia-induced cellular remodeling via Akt/Nrf2/heme oxygenase-1 pathway. J Mol Cell Cardiol. 2015;82:84-92. doi:10.1016/j.yjmcc.2015.03.004. 10. Wang W, Zhu Y, Yi J, Cheng W. Nkx2.5/CARP signaling pathway contributes to the regulation of ion channel remodeling induced by rapid pacing in rat atrial myocytes. Mol Med Rep. 2016;14(4):3848-3854. doi:10.3892/mmr.2016.5727. 11. Tang M, Yuan W, Fan X, et al. Pygopus maintains heart function in aging Drosophila independently of canonical Wnt signaling. Circ Cardiovasc Genet. 2013;6(5):472-480. doi:10.1161/CIRCGENETICS.113.000253. 12. Zhang H, Bryson V, Luo N, Sun AY, Rosenberg P. STIM1-Ca2+ signaling in coronary sinus cardiomyocytes contributes to interatrial conduction. Cell Calcium. 2020;87:102163. doi:10.1016/j.ceca.2020.102163. 13. Yan J, Thomson JK, Zhao W, Gao X, Huang F, Chen B, Liang Q, Song LS, Fill M, Ai X. Role of Stress Kinase JNK in Binge Alcohol-Evoked Atrial Arrhythmia. J Am Coll Cardiol. 2018;71(13):1459-1470. doi: 10.1016/j.jacc.2018.01.060. | |
| **136 review articles:**   1. Denham NC, Pearman CM, Caldwell JL, et al. Calcium in the Pathophysiology of Atrial Fibrillation and Heart Failure. Front Physiol. 2018;9:1380. doi:10.3389/fphys.2018.01380. 2. Jalife J, Kaur K. Atrial remodeling, fibrosis, and atrial fibrillation. Trends Cardiovasc Med. 2015;25(6):475-484. doi:10.1016/j.tcm.2014.12.015. 3. Stephan LS, Almeida ED, Markoski MM, Garavaglia J, Marcadenti A. Red Wine, Resveratrol and Atrial Fibrillation. Nutrients. 2017;9(11):1190. doi:10.3390/nu9111190. 4. Andrade J, Khairy P, Dobrev D, Nattel S. The clinical profile and pathophysiology of atrial fibrillation: relationships among clinical features, epidemiology, and mechanisms. Circ Res. 2014;114(9):1453-1468. doi:10.1161/CIRCRESAHA.114.303211. 5. Heijman J, Voigt N, Nattel S, Dobrev D. Cellular and molecular electrophysiology of atrial fibrillation initiation, maintenance, and progression. Circ Res. 2014;114(9):1483-1499. doi:10.1161/CIRCRESAHA.114.302226. 6. Nattel S, Harada M. Atrial remodeling and atrial fibrillation: recent advances and translational perspectives. J Am Coll Cardiol. 2014;63(22):2335-2345. doi:10.1016/j.jacc.2014.02.555. 7. Smith GL, Eisner DA. Calcium Buffering in the Heart in Health and Disease. Circulation. 2019;139(20):2358-2371. doi:10.1161/CIRCULATIONAHA.118.039329. 8. Greiser M. Calcium signalling silencing in atrial fibrillation. J Physiol. 2017;595(12):4009-4017. doi:10.1113/JP273045. 9. Landstrom AP, Dobrev D, Wehrens XHT. Calcium Signaling and Cardiac Arrhythmias. Circ Res. 2017;120(12):1969-1993. doi:10.1161/CIRCRESAHA.117.310083. 10. Atrial Electrophysiological Remodeling and Fibrillation in Heart Failure. Sandeep V. Pandit and Antony J. Workman. Clin Med Insights Cardiol. 2016;10(Suppl 1):41-46. 11. Husser D, Ueberham L, Dinov B, et al. Genomic contributors to atrial electroanatomical remodeling and atrial fibrillation progression: Pathway enrichment analysis of GWAS data. Sci Rep. 2016;6:36630. Published 2016 Nov 18. doi:10.1038/srep36630. 12. Heijman J, Voigt N, Ghezelbash S, Schirmer I, Dobrev D. Calcium Handling Abnormalities as a Target for Atrial Fibrillation Therapeutics: How Close to Clinical Implementation? J Cardiovasc Pharmacol. 2015;66(6):515-522. doi:10.1097/FJC.0000000000000253. 13. Chang, K., Trayanova, N. Mechanisms of arrhythmogenesis related to calcium-driven alternans in a model of human atrial fibrillation. Sci Rep 6, 36395 (2016). doi: 10.1038/srep36395. 14. Nattel S, Dobrev D. Deciphering the fundamental mechanisms of atrial fibrillation: a quest for over a century. Cardiovasc Res. 2016;109(4):465-466. doi:10.1093/cvr/cvw028. 15. Hamilton S, Terentyev D. Altered Intracellular Calcium Homeostasis and Arrhythmogenesis in the Aged Heart. Int J Mol Sci. 2019;20(10):2386. doi:10.3390/ijms20102386. 16. Weirich J. Remodeling des Altersherzens: Sinusknotendysfunktion und Vorhofflimmern [Remodeling of the aging heart : Sinus node dysfunction and atrial fibrillation]. Herzschrittmacherther Elektrophysiol. 2017;28(1):29-38. doi:10.1007/s00399-017-0485-3. 17. Hegyi B, Bers DM, Bossuyt J. CaMKII signaling in heart diseases: Emerging role in diabetic cardiomyopathy. J Mol Cell Cardiol. 2019;127:246-259. doi:10.1016/j.yjmcc.2019.01.001. 18. Gomez-Hurtado N, Knollmann BC. Calcium in atrial fibrillation - pulling the trigger or not?. J Clin Invest. 2014;124(11):4684-4686. doi:10.1172/JCI77986. 19. Shiferaw Y, Aistrup GL, Wasserstrom JA. Mechanism for Triggered Waves in Atrial Myocytes. Biophys J. 2017;113(3):656-670. doi:10.1016/j.bpj.2017.06.026. 20. Wijesurendra RS, Casadei B. Atrial fibrillation: effects beyond the atrium. Cardiovasc Res. 2015;105(3):238-247. doi:10.1093/cvr/cvv001. 21. Schotten U, Dobrev D, Platonov PG, Kottkamp H, Hindricks G. Current controversies in determining the main mechanisms of atrial fibrillation. J Intern Med. 2016;279(5):428-438. doi:10.1111/joim.12492. 22. Brandenburg S, Arakel EC, Schwappach B, Lehnart SE. The molecular and functional identities of atrial cardiomyocytes in health and disease. Biochim Biophys Acta. 2016;1863(7 Pt B):1882-1893. doi:10.1016/j.bbamcr.2015.11.025. 23. Scott L Jr, Li N, Dobrev D. Role of inflammatory signaling in atrial fibrillation. Int J Cardiol. 2019;287:195-200. doi:10.1016/j.ijcard.2018.10.020. 24. Ninio DM, Saint DA. The role of stretch-activated channels in atrial fibrillation and the impact of intracellular acidosis. Prog Biophys Mol Biol. 2008;97(2-3):401-416. doi:10.1016/j.pbiomolbio.2008.02.016. 25. Brocklehurst P, Ni H, Zhang H, Ye J. Electro-mechanical dynamics of spiral waves in a discrete 2D model of human atrial tissue. PLoS One. 2017;12(5):e0176607. doi:10.1371/journal.pone.0176607. 26. Chen PS, Tan AY. Autonomic nerve activity and atrial fibrillation. Heart Rhythm. 2007;4(3 Suppl):S61-S64. doi:10.1016/j.hrthm.2006.12.006. 27. Kettlewell S, Burton FL, Smith GL, Workman AJ. Chronic myocardial infarction promotes atrial action potential alternans, afterdepolarizations, and fibrillation. Cardiovasc Res. 2013;99(1):215-224. doi:10.1093/cvr/cvt087. 28. Colman MA. Arrhythmia mechanisms and spontaneous calcium release: Bi-directional coupling between re-entrant and focal excitation. PLoS Comput Biol. 2019;15(8):e1007260. Published 2019 Aug 8. doi:10.1371/journal.pcbi.1007260. 29. Workman AJ. Cardiac adrenergic control and atrial fibrillation. Naunyn Schmiedebergs Arch Pharmacol. 2010;381(3):235-249. doi:10.1007/s00210-009-0474-0. 30. Donahue JK. Biological Therapies for Atrial Fibrillation: Ready for Prime Time?. J Cardiovasc Pharmacol. 2016;67(1):19-25. doi:10.1097/FJC.0000000000000293. 31. Dobrev D, Wehrens XH. Calmodulin kinase II, sarcoplasmic reticulum Ca2+ leak, and atrial fibrillation. Trends Cardiovasc Med. 2010;20(1):30-34. doi:10.1016/j.tcm.2010.03.004. 32. Husser D, Büttner P, Ueberham L, et al. Genomic Contributors to Rhythm Outcome of Atrial Fibrillation Catheter Ablation - Pathway Enrichment Analysis of GWAS Data. PLoS One. 2016;11(11):e0167008. Published 2016 Nov 21. doi:10.1371/journal.pone.0167008. 33. Dobrev D, Voigt N, Wehrens XH. The ryanodine receptor channel as a molecular motif in atrial fibrillation: pathophysiological and therapeutic implications. Cardiovasc Res. 2011;89(4):734-743. doi:10.1093/cvr/cvq324. 34. Van Wagoner, D. R., Pond, A. L., Lamorgese, M., Rossie, S. S., McCarthy, P. M., & Nerbonne, J. M. Atrial L-type Ca2+ currents and human atrial fibrillation. Circulation research. 1999;85(5): 428-436. doi: 10.1161/01.RES.85.5.428. 35. Sridhar A, Nishijima Y, Terentyev D, et al. Chronic heart failure and the substrate for atrial fibrillation. Cardiovasc Res. 2009;84(2):227-236. doi:10.1093/cvr/cvp216. 36. Yue L, Xie J, Nattel S. Molecular determinants of cardiac fibroblast electrical function and therapeutic implications for atrial fibrillation. Cardiovasc Res. 2011;89(4):744-753. doi:10.1093/cvr/cvq329. 37. Schönleitner P, Schotten U, Antoons G. Mechanosensitivity of microdomain calcium signalling in the heart. Prog Biophys Mol Biol. 2017;130(Pt B):288-301. doi:10.1016/j.pbiomolbio.2017.06.013. 38. Goyal R, Singh A, Fan R. Not Your Usual Pre-Excitation. Circulation. 2017;135(18):1759-1761. doi:10.1161/CIRCULATIONAHA.117.028386. 39. Harada M, Luo X, Murohara T, Yang B, Dobrev D, Nattel S. MicroRNA regulation and cardiac calcium signaling: role in cardiac disease and therapeutic potential. Circ Res. 2014;114(4):689-705. doi:10.1161/CIRCRESAHA.114.301798. 40. Dobrev D. 5-hydroxytryptamine and atrial arrhythmogenesis: a "culprit mechanism" or bystander in patients with chronic atrial fibrillation? J Mol Cell Cardiol. 2007;42(1):51-53. doi:10.1016/j.yjmcc.2006.09.014. 41. Kanaporis G, Blatter LA. Alternans in atria: Mechanisms and clinical relevance. Medicina (Kaunas). 2017;53(3):139-149. doi:10.1016/j.medici.2017.04.004. 42. Bootman MD, Smyrnias I, Thul R, Coombes S, Roderick HL. Atrial cardiomyocyte calcium signalling. Biochim Biophys Acta. 2011;1813(5):922-934. doi:10.1016/j.bbamcr.2011.01.030. 43. Whittaker DG, Ni H, El Harchi A, Hancox JC, Zhang H. Atrial arrhythmogenicity of KCNJ2 mutations in short QT syndrome: Insights from virtual human atria [published correction appears in PLoS Comput Biol. 2019;15(6):e1007145]. PLoS Comput Biol. 2017;13(6):e1005593. doi:10.1371/journal.pcbi.1005593. 44. Sutanto H, Dobrev D, Heijman J. Resveratrol: an effective pharmacological agent to prevent inflammation-induced atrial fibrillation? Naunyn Schmiedebergs Arch Pharmacol. 2018 Nov;391(11):1163-1167. doi: 10.1007/s00210-018-1566-5. 45. Tanaka H. [Fluorescence imaging of the living heart for understanding the basis of arrhythmias]. Nihon Yakurigaku Zasshi. 2019;154(4):171-177. doi: 10.1254/fpj.154.171. 46. Ripplinger CM. A leap(frog) forward in understanding focal arrhythmia. J Physiol. 2015;593(6):1383-1384. doi:10.1113/jphysiol.2014.287128. 47. Voigt N, Nattel S, Dobrev D. Proarrhythmic atrial calcium cycling in the diseased heart. Adv Exp Med Biol. 2012;740:1175-1191. doi:10.1007/978-94-007-2888-2_53. 48. Joung B, Chen PS. Function and dysfunction of human sinoatrial node. Korean Circ J. 2015;45(3):184-191. doi:10.4070/kcj.2015.45.3.184. 49. Zhu H, Xue H, Jin QH, Guo J, Chen YD. Increased expression of ryanodine receptor type-2 during atrial fibrillation by miR-106-25 cluster independent mechanism. Exp Cell Res. 2019;375(2):113-117. doi:10.1016/j.yexcr.2018.11.025. 50. Li Q, O'Neill SC, Tao T, Li Y, Eisner D, Zhang H. Mechanisms by which cytoplasmic calcium wave propagation and alternans are generated in cardiac atrial myocytes lacking T-tubules-insights from a simulation study. Biophys J. 2012;102(7):1471-82. doi: 10.1016/j.bpj.2012.03.007. 51. Boulkroun S, Fernandes-Rosa FL, Zennaro MC. Molecular and Cellular Mechanisms of Aldosterone Producing Adenoma Development. Front Endocrinol (Lausanne). 2015;6:95. doi:10.3389/fendo.2015.00095. 52. Nattel S. Ionic determinants of atrial fibrillation and Ca2+ channel abnormalities : cause, consequence, or innocent bystander?. Circ Res. 1999;85(5):473-476. doi:10.1161/01.res.85.5.473. 53. Rankin AC, Workman AJ. Duration of heart failure and the risk of atrial fibrillation: different mechanisms at different times? Cardiovasc Res. 2009;84(2):180-181. doi:10.1093/cvr/cvp299. 54. Hatem S. Mécanismes moléculaires et cellulaires de la fibrillation auriculaire : existe-t-il de nouvelles stratégies thérapeutiques ? [Biology of the substrate of atrial fibrillation]. Biol Aujourdhui. 2012;206(1):5-9. doi:10.1051/jbio/2012004. 55. Simon JN, Duglan D, Casadei B, Carnicer R. Nitric oxide synthase regulation of cardiac excitation-contraction coupling in health and disease. J Mol Cell Cardiol. 2014;73:80-91. doi:10.1016/j.yjmcc.2014.03.004. 56. Carnicer R, Crabtree MJ, Sivakumaran V, Casadei B, Kass DA. Nitric oxide synthases in heart failure. Antioxid Redox Signal. 2013;18(9):1078-1099. doi:10.1089/ars.2012.4824. 57. Dun W, Boyden PA. Aged atria: electrical remodeling conducive to atrial fibrillation. J Interv Card Electrophysiol. 2009;25(1):9-18. doi:10.1007/s10840-008-9358-3. 58. Gawałko M, Balsam P, Lodziński P, Grabowski M, Krzowski B, Opolski G, Kosiuk J. Cardiac Arrhythmias in Autoimmune Diseases. Circ J. 2020;84(5):685-694. doi: 10.1253/circj.CJ-19-0705. 59. Umehara S, Tan X, Okamoto Y, et al. Mechanisms Underlying Spontaneous Action Potential Generation Induced by Catecholamine in Pulmonary Vein Cardiomyocytes: A Simulation Study. Int J Mol Sci. 2019;20(12):2913. doi:10.3390/ijms20122913. 60. Kaneko N, Matsuda R, Hata Y, Shimamoto K. Pharmacological characteristics and clinical applications of K201. Curr Clin Pharmacol. 2009;4(2):126-131. doi:10.2174/157488409788184972. 61. Ferrantini C, Crocini C, Coppini R, et al. The transverse-axial tubular system of cardiomyocytes. Cell Mol Life Sci. 2013;70(24):4695-4710. doi:10.1007/s00018-013-1410-5. 62. Gonzalez DR, Treuer A, Sun QA, Stamler JS, Hare JM. S-Nitrosylation of cardiac ion channels. J Cardiovasc Pharmacol. 2009;54(3):188-195. doi:10.1097/FJC.0b013e3181b72c9f. 63. Tanaka H, Matsuyama TA, Takamatsu T. Towards an integrated understanding of cardiac arrhythmogenesis - Growing roles of experimental pathology. Pathol Int. 2017;67(1):8-16. doi:10.1111/pin.12487. 64. Chen PS, Joung B, Shinohara T, Das M, Chen Z, Lin SF. The initiation of the heart beat. Circ J. 2010;74(2):221-225. doi:10.1253/circj.cj-09-0712. 65. Burashnikov A, Antzelevitch C. New developments in atrial antiarrhythmic drug therapy. Nat Rev Cardiol. 2010;7(3):139-148. doi:10.1038/nrcardio.2009.245. 66. Anumonwo JM. Activation of I sac promotes atrial fibrillation initiation and perpetuation: is this a stretch?. Heart Rhythm. 2011;8(3):437-438. doi:10.1016/j.hrthm.2010.12.020. 67. Nishida K, Qi XY, Wakili R, Comtois P, Chartier D, Harada M, Iwasaki YK, Romeo P, Maguy A, Dobrev D, Michael G, Talajic M, Nattel S. Mechanisms of atrial tachyarrhythmias associated with coronary artery occlusion in a chronic canine model. Circulation. 2011;123(2):137-46. doi: 10.1161/CIRCULATIONAHA.110.972778. 68. Louch WE, Sejersted OM, Swift F. There goes the neighborhood: pathological alterations in T-tubule morphology and consequences for cardiomyocyte Ca2+ handling. J Biomed Biotechnol. 2010;2010:503906. doi:10.1155/2010/503906. 69. Remo BF, Fishman GI. A colorful explanation for atrial arrhythmias. Pigment Cell Melanoma Res. 2010;23(1):3-4. doi:10.1111/j.1755-148X.2009.00658.x. 70. Dillmann W. Cardiac hypertrophy and thyroid hormone signaling. Heart Fail Rev. 2010;15(2):125-132. doi:10.1007/s10741-008-9125-7. 71. Marsh JD, Telemaque S, Rhee SW, Stimers JR, Rusch NJ. Delivery of ion channel genes to treat cardiovascular diseases. Trans Am Clin Climatol Assoc. 2008;119:171-183. 72. Gheorghiade M, Adams KF Jr, Colucci WS. Digoxin in the management of cardiovascular disorders. Circulation. 2004;109(24):2959-2964. doi:10.1161/01.CIR.0000132482.95686.87. 73. Zicha S, Tsuji Y, Shiroshita-Takeshita A, Nattel S. Beta-blockers as antiarrhythmic agents. Handb Exp Pharmacol. 2006;(171):235-266. 74. Ochi R, Gupte SA. Ryanodine receptor: a novel therapeutic target in heart disease. Recent Pat Cardiovasc Drug Discov. 2007;2(2):110-118. doi:10.2174/157489007780832524. 75. Bollmann A, Husser D, Stridh M, et al. Atrial fibrillatory rate and risk of left atrial thrombus in atrial fibrillation. Europace. 2007;9(8):621-626. doi:10.1093/europace/eum125. 76. Schwartz M, Rodman D, Lowenstein SR. Recognition and treatment of multifocal atrial tachycardia: a critical review. J Emerg Med. 1994;12(3):353-360. doi:10.1016/0736-4679(94)90278-x. 77. Kanaporis G, Blatter LA. The mechanisms of calcium cycling and action potential dynamics in cardiac alternans. Circ Res. 2015;116(5):846-856. doi:10.1161/CIRCRESAHA.116.305404. 78. Morales GX, Bodiwala K, Elayi CS. Giant J-wave (Osborn wave) unrelated to hypothermia. Europace. 2011;13(2):283. doi:10.1093/europace/euq424. 79. Crotti L, Odening KE, Sanguinetti MC. Heritable arrhythmias associated with abnormal function of cardiac potassium channels. Cardiovasc Res. 2020;116(9):1542-1556. doi:10.1093/cvr/cvaa068. 80. Markham A, Brogden RN. Diltiazem. A review of its pharmacology and therapeutic use in older patients. Drugs Aging. 1993;3(4):363-390. doi:10.2165/00002512-199303040-00007. 81. Donnan GA. Therapy in cerebrovascular disease: current status and future directions. Med J Aust. 1991;155(8):563-571. 82. Libbus I, Rosenbaum DS. Remodeling of cardiac repolarization: mechanisms and noll 83. Noll G, Kaufmann U, Wenzel RR, Lüscher TF. Therapeutische Massnahmen nach akutem Myokardinfarkt: differentieller Einsatz von PTCA, Chirurgie und Medikamenten [Therapeutic measures following acute myocardial infarct: differential use of PTCA, surgery and drugs]. Schweiz Med Wochenschr. 1996;126(5):164-176. 84. Harada M, Tadevosyan A, Qi X, et al. Atrial Fibrillation Activates AMP-Dependent Protein Kinase and its Regulation of Cellular Calcium Handling: Potential Role in Metabolic Adaptation and Prevention of Progression. J Am Coll Cardiol. 2015;66(1):47-58. doi:10.1016/j.jacc.2015.04.056. 85. Valdivia HH. Mechanisms of cardiac alternans in atrial cells: intracellular Ca2⁺ disturbances lead the way. Circ Res. 2015;116(5):778-780. doi:10.1161/CIRCRESAHA.115.305923. 86. Sun H, Gaspo R, Leblanc N, Nattel S. Cellular mechanisms of atrial contractile dysfunction caused by sustained atrial tachycardia. Circulation. 1998;98(7):719-727. doi:10.1161/01.cir.98.7.719. 87. Dong G, Liang F, Sun B, et al. Presence and function of stress granules in atrial fibrillation. PLoS One. 2019;14(4):e0213769. Published 2019 Apr 3. doi:10.1371/journal.pone.0213769. 88. Francis J, Antzelevitch C. Atrial fibrillation and Brugada syndrome. J Am Coll Cardiol. 2008;51(12):1149-1153. doi:10.1016/j.jacc.2007.10.062. 89. Christ T, Rozmaritsa N, Engel A, et al. Arrhythmias, elicited by catecholamines and serotonin, vanish in human chronic atrial fibrillation [published correction appears in Proc Natl Acad Sci U S A. 2014 Sep 23;111(38):14003]. Proc Natl Acad Sci U S A. 2014;111(30):11193-11198. doi:10.1073/pnas.1324132111. 90. Merritt RE, Shrager JB. Prophylaxis and management of atrial fibrillation after general thoracic surgery. Thorac Surg Clin. 2012;22(1):13-v. doi:10.1016/j.thorsurg.2011.08.016. 91. Kanjwal Y, Imran N, Grubb B. Deglutition induced atrial tachycardia and atrial fibrillation. Pacing Clin Electrophysiol. 2007;30(12):1575-1578. doi:10.1111/j.1540-8159.2007.00911.x. 92. Bateman RM, Sharpe MD, Jagger JE, et al. 36th International Symposium on Intensive Care and Emergency Medicine : Brussels, Belgium. 15-18 March 2016 [published correction appears in Crit Care. 2016 Oct 24;20:347]. Crit Care. 2016;20(Suppl 2):94. Published 2016 Apr 20. doi:10.1186/s13054-016-1208-6. 93. McDivitt JD, Barstow C. Cardiovascular Disease Update: Atrial Fibrillation. FP Essent. 2017;454:11-17. 94. Tian G, Sun Y, Liu S, et al. Therapeutic Effects of Wenxin Keli in Cardiovascular Diseases: An Experimental and Mechanism Overview. Front Pharmacol. 2018;9:1005. doi:10.3389/fphar.2018.01005. 95. Khan IA. Atrial stunning: determinants and cellular mechanisms. Am Heart J. 2003;145(5):787-794. doi:10.1016/S0002-8703(03)00086-3. 96. Shin DG, Cho I, Hartaigh Bó, et al. Cardiovascular Events of Electrical Cardioversion Under Optimal Anticoagulation in Atrial Fibrillation: The Multicenter Analysis. Yonsei Med J. 2015;56(6):1552-1558. doi:10.3349/ymj.2015.56.6.1552. 97. Khan IA. Atrial stunning: basics and clinical considerations. Int J Cardiol. 2003;92(2-3):113-128. doi:10.1016/s0167-5273(03)00107-4. 98. Mayson SE, Greenspon AJ, Adams S, et al. The changing face of postoperative atrial fibrillation prevention: a review of current medical therapy. Cardiol Rev. 2007;15(5):231-241. doi:10.1097/CRD.0b013e31813e62bb. 99. Bosch RF, Zeng X, Grammer JB, Popovic K, Mewis C, Kühlkamp V. Ionic mechanisms of electrical remodeling in human atrial fibrillation. Cardiovasc Res. 1999;44(1):121-131. doi:10.1016/s0008-6363(99)00178-9. 100. Ho KM, Sheridan DJ, Paterson T. Use of intravenous magnesium to treat acute onset atrial fibrillation: a meta-analysis. Heart. 2007;93(11):1433-1440. doi:10.1136/hrt.2006.111492. 101. Rücker-Martin C, Pecker F, Godreau D, Hatem SN. Dedifferentiation of atrial myocytes during atrial fibrillation: role of fibroblast proliferation in vitro. Cardiovasc Res. 2002;55(1):38-52. doi:10.1016/s0008-6363(02)00338-3. 102. Van Wagoner DR, Nerbonne JM. Molecular basis of electrical remodeling in atrial fibrillation. J Mol Cell Cardiol. 2000;32(6):1101-1117. doi:10.1006/jmcc.2000.1147. 103. Xi Y, Wu G, Ai T, et al. Ionic mechanisms underlying the effects of vasoactive intestinal polypeptide on canine atrial myocardium. Circ Arrhythm Electrophysiol. 2013;6(5):976-983. doi:10.1161/CIRCEP.113.000518. 104. Gravez B, Tarjus A, Jaisser F. Mineralocorticoid receptor and cardiac arrhythmia. Clin Exp Pharmacol Physiol. 2013;40(12):910-915. doi:10.1111/1440-1681.12156. 105. Abrol R, Page RL. Azimilide dihydrochloride: a new class III anti-arrhythmic agent. Expert Opin Investig Drugs. 2000;9(11):2705-2715. doi:10.1517/13543784.9.11.2705. 106. Connell P, Word TA, Wehrens XHT. Targeting pathological leak of ryanodine receptors: preclinical progress and the potential impact on treatments for cardiac arrhythmias and heart failure. Expert Opin Ther Targets. 2020;24(1):25-36. doi:10.1080/14728222.2020.1708326. 107. Borchard U, Hafner D. Ionenkanäle und Herzrhythmusstörungen [Ion channels and arrhythmias]. Z Kardiol. 2000;89 Suppl 3:6-12. 108. Gloor HO. Medikamentöse Therapie des Vorhofflimmerns [Drug therapy of atrial fibrillation]. Schweiz Med Wochenschr. 1996 Oct 12;126(41):1739-47. 109. Podesser B, Schwarzacher S, Zwölfer W, et al. Combined perioperative infusion of nifedipine and metoprolol provides antiischemic and antiarrhythmic protection in patients undergoing elective aortocoronary bypass surgery. Thorac Cardiovasc Surg. 1993;41(3):173-179. doi:10.1055/s-2007-1013848. 110. Zhou Y, Wu HJ, Zhang YH, Sun HY, Wong TM, Li GR. Ionic mechanisms underlying cardiac toxicity of the organochloride solvent trichloromethane. Toxicology. 2011;290(2-3):295-304. doi:10.1016/j.tox.2011.10.009. 111. Li D, Zhang L, Kneller J, Nattel S. Potential ionic mechanism for repolarization differences between canine right and left atrium. Circ Res. 2001;88(11):1168-1175. doi:10.1161/hh1101.091266. 112. Thodeti CK, Paruchuri S, Meszaros JG. A TRP to cardiac fibroblast differentiation. Channels (Austin). 2013;7(3):211-214. doi:10.4161/chan.24328. 113. Sueda S, Kohno H, Oshita A, Izoe Y, Nomoto T, Fukuda H. Vasospastic heart failure: multiple spasm may cause transient heart failure? J Cardiol. 2009;54(3):452-459. doi:10.1016/j.jjcc.2009.07.007. 114. Vlase H, Lungu G, Vlase L. Cardiac disturbances in thyrotoxicosis: diagnosis, incidence, clinical features and management. Endocrinologie. 1991;29(3-4):155-160. 115. Ai X. SR calcium handling dysfunction, stress-response signaling pathways, and atrial fibrillation. Front Physiol. 2015;6:46. Published 2015 Feb 19. doi:10.3389/fphys.2015.00046. 116. Bers DM. Stabilizing ryanodine receptor gating quiets arrhythmogenic events in human heart failure and atrial fibrillation. Heart Rhythm. 2017;14(3):420-421. doi:10.1016/j.hrthm.2016.10.005. 117. Dobrev D. Unique cardiomyocyte ultrastructure in atria: Role of T tubules in subcellular Ca2+ signaling and atrial arrhythmogenesis. Heart Rhythm. 2017;14(2):282-283. doi:10.1016/j.hrthm.2016.10.013. 118. Blatter LA, Kockskämper J, Sheehan KA, Zima AV, Hüser J, Lipsius SL. Local calcium gradients during excitation-contraction coupling and alternans in atrial myocytes. J Physiol. 2003;546(Pt 1):19-31. doi:10.1113/jphysiol.2002.025239. 119. Trafford AW, Clarke JD, Richards MA, Eisner DA, Dibb KM. Calcium signalling microdomains and the t-tubular system in atrial mycoytes: potential roles in cardiac disease and arrhythmias. Cardiovasc Res. 2013;98(2):192-203. doi:10.1093/cvr/cvt018. 120. Voigt N, Dobrev D. Cellular and molecular correlates of ectopic activity in patients with atrial fibrillation. Europace. 2012;14 Suppl 5:v97-v105. doi:10.1093/europace/eus282. 121. Dobrev D. Atrial Ca2+ signaling in atrial fibrillation as an antiarrhythmic drug target. Naunyn Schmiedebergs Arch Pharmacol. 2010;381(3):195-206. doi:10.1007/s00210-009-0457-1. 122. Greiser M, Schotten U. Dynamic remodeling of intracellular Ca²⁺ signaling during atrial fibrillation. J Mol Cell Cardiol. 2013;58:134-142. doi:10.1016/j.yjmcc.2012.12.020. 123. Rietdorf K, Masoud S, McDonald F, Sanderson MJ, Bootman MD. Pulmonary vein sleeve cell excitation-contraction-coupling becomes dysynchronized by spontaneous calcium transients. Biochem Soc Trans. 2015;43(3):410-416. doi:10.1042/BST20140299. 124. Luczak ED, Anderson ME. CaMKII oxidative activation and the pathogenesis of cardiac disease. J Mol Cell Cardiol. 2014;73:112-116. doi:10.1016/j.yjmcc.2014.02.004. 125. Kim GH. MicroRNA regulation of cardiac conduction and arrhythmias. Transl Res. 2013;161(5):381-392. doi:10.1016/j.trsl.2012.12.004. 126. Rottbauer W, Baker K, Wo ZG, Mohideen MA, Cantiello HF, Fishman MC. Growth and function of the embryonic heart depend upon the cardiac-specific L-type calcium channel alpha1 subunit. Dev Cell. 2001;1(2):265-275. doi:10.1016/s1534-5807(01)00023-5. 127. Wan E, Abrams J, Weinberg RL, et al. Aberrant sodium influx causes cardiomyopathy and atrial fibrillation in mice. J Clin Invest. 2016;126(1):112-122. doi:10.1172/JCI84669. 128. Van Wagoner DR. Electrophysiological remodeling in human atrial fibrillation. Pacing Clin Electrophysiol. 2003;26(7):1572-1575. doi:10.1046/j.1460-9592.2003.t01-1-00234.x. 129. Huang CF, Chen YC, Yeh HI, Chen SA. Mononucleated and binucleated cardiomyocytes in left atrium and pulmonary vein have different electrical activity and calcium dynamics. Prog Biophys Mol Biol. 2012;108(1-2):64-73. doi:10.1016/j.pbiomolbio.2011.09.007. 130. Zhang J, Tao R, Campbell KF, et al. Functional cardiac fibroblasts derived from human pluripotent stem cells via second heart field progenitors. Nat Commun. 2019;10(1):2238. Published 2019 May 20. doi:10.1038/s41467-019-09831-5. 131. Pandit SV, Workman AJ. Atrial Electrophysiological Remodeling and Fibrillation in Heart Failure. Clin Med Insights Cardiol. 2016;10(Suppl 1):41-46. Published 2016 Oct 31. doi:10.4137/CMC.S39713. 132. Choi E, Cha MJ, Hwang KC. Roles of Calcium Regulating MicroRNAs in Cardiac Ischemia-Reperfusion Injury. Cells. 2014;3(3):899-913. Published 2014 Sep 11. doi:10.3390/cells3030899. 133. Seferović PM, Ristić AD, Maksimović R, et al. Cardiac arrhythmias and conduction disturbances in autoimmune rheumatic diseases. Rheumatology. 2006;45:iv39-iv42. doi:10.1093/rheumatology/kel315. 134. Xi Y, Cheng J. Dysfunction of the autonomic nervous system in atrial fibrillation. J Thorac Dis. 2015;7(2):193-198. doi:10.3978/j.issn.2072-1439.2015.01.12. 135. Musco S, Conway EL, Kowey PR. Drug therapy for atrial fibrillation. Med Clin North Am. 2008;92(1):121-xi. doi:10.1016/j.mcna.2007.08.002. 136. Kneller J, Sun H, Leblanc N, Nattel S. Remodeling of Ca(2+)-handling by atrial tachycardia: evidence for a role in loss of rate-adaptation. Cardiovasc Res. 2002;54(2):416-426. doi:10.1016/s0008-6363(02)00274-2. | |
| **25 did not report on atrial fibrillation:**  [1] Aistrup GL, Arora R, Grubb S, et al. Triggered intracellular calcium waves in dog and human left atrial myocytes from normal and failing hearts. Cardiovasc Res. 2017;113(13):1688-1699. doi:10.1093/cvr/cvx167.  [2] Kim JC, Son MJ, Wang J, Woo SH. Regulation of cardiac Ca2+ and ion channels by shear mechanotransduction. Arch Pharm Res. 2017;40(7):783-795. doi:10.1007/s12272-017-0929-7.  [3] Fujii A, Inoue K, Nagai T, et al. Clinical Utility of Atrial Electromechanical Conduction Time Measured with Speckle Tracking Echocardiography after Catheter Ablation in Patients with Atrial Fibrillation: A Validation Study with Electroanatomical Mapping. Echocardiography. 2016;33(9):1317-1325. doi:10.1111/echo.13259.  [4] Efe TH, Cimen T, Ertem AG, et al. Atrial Electromechanical Properties in Inflammatory Bowel Disease. Echocardiography. 2016;33(9):1309-1316. doi:10.1111/echo.13261.  [5] Shiferaw Y, Aistrup GL, Louch WE, Wasserstrom JA. Remodeling Promotes Proarrhythmic Disruption of Calcium Homeostasis in Failing Atrial Myocytes. Biophys J. 2020;118(2):476-491. doi:10.1016/j.bpj.2019.12.012.  [6] Hannon JD, Housmans PR. Ca2+: is there something new for the cardiovascular anesthesiologist?. Curr Opin Anaesthesiol. 2009;22(1):114-120. doi:10.1097/ACO.0b013e32831a43c7.  [7] Mao T, Zhang J, Qiao Y, Liu B, Zhang S. Uncovering Synergistic Mechanism of Chinese Herbal Medicine in the Treatment of Atrial Fibrillation with Obstructive Sleep Apnea Hypopnea Syndrome by Network Pharmacology. Evid Based Complement Alternat Med. 2019;2019:8691608. Published 2019 Dec 23. doi:10.1155/2019/8691608.  [8] Zimering MB, Pan Z. Increased Neuronal Depolarization Evoked by Autoantibodies in Diabetic Obstructive Sleep Apnea: Role for Inflammatory Protease(s) in Generation of Neurotoxic Immunoglobulin Fragment. J Endocrinol Diabetes. 2017;4(1):10.15226/2374-6890/4/1/00168. doi:10.15226/2374-6890/4/1/00168.  [9] Murata M, Cingolani E, McDonald AD, Donahue JK, Marbán E. Creation of a genetic calcium channel blocker by targeted gem gene transfer in the heart. Circ Res. 2004;95(4):398-405. doi:10.1161/01.RES.0000138449.85324.c5.  [10] Francia P, Adduci C, Ricotta A, et al. Common genetic variants in selected Ca²⁺ signaling genes and the risk of appropriate ICD interventions in patients with heart failure. J Interv Card Electrophysiol. 2013;38(3):169-177. doi:10.1007/s10840-013-9827-1.  [11] Dixen U, Wallevik L, Hansen MS, et al. Prolonged signal-averaged P wave duration as a prognostic marker for morbidity and mortality in patients with congestive heart failure. Scand Cardiovasc J. 2003;37(4):193-198. doi:10.1080/14017430310002202.  [12] Cha TJ, Ehrlich JR, Zhang L, et al. Dissociation between ionic remodeling and ability to sustain atrial fibrillation during recovery from experimental congestive heart failure. Circulation. 2004;109(3):412-418. doi:10.1161/01.CIR.0000109501.47603.0C.  [13] Li D, Melnyk P, Feng J, et al. Effects of experimental heart failure on atrial cellular and ionic electrophysiology. Circulation. 2000;101(22):2631-2638. doi:10.1161/01.cir.101.22.2631.  [14] Jones RJ, Brace SR, Vander Tuin EL. Probable propafenone-induced transient global amnesia. Ann Pharmacother. 1995;29(6):586-590. doi:10.1177/106002809502900606.  [15] Shepard RK, Natale A, Stambler BS, Wood MA, Gilligan DM, Ellenbogen KA. Physiology of the escape rhythm after radiofrequency atrioventricular junctional ablation. Pacing Clin Electrophysiol. 1998;21(5):1085-1092. doi:10.1111/j.1540-8159.1998.tb00154.x.  [16] Leow MK, Loh KC, Kon WY, Wong DE, Tan BS, Soon PC. Clinical utility of selective intra-arterial calcium-stimulated hepatic venous sampling in regionalisation of insulinomas--the Singapore experience. Ann Acad Med Singapore. 2003;32(1):86-91.  [17] Nof E, Belhassen B, Arad M, et al. Postpacing abnormal repolarization in catecholaminergic polymorphic ventricular tachycardia associated with a mutation in the cardiac ryanodine receptor gene. Heart Rhythm. 2011;8(10):1546-1552. doi:10.1016/j.hrthm.2011.05.016.  [18] Han L, Li J. Canonical transient receptor potential 3 channels in atrial fibrillation. Eur J Pharmacol. 2018;837:1-7. doi:10.1016/j.ejphar.2018.08.030.  [19] Nikolova-Krstevski V, Wagner S, Yu ZY, et al. Endocardial TRPC-6 Channels Act as Atrial Mechanosensors and Load-Dependent Modulators of Endocardial/Myocardial Cross-Talk. JACC Basic Transl Sci. 2017;2(5):575-590. Published 2017 Oct 30. doi:10.1016/j.jacbts.2017.05.006.  [20] Tadevosyan A, Xiao J, Surinkaew S, et al. Intracellular Angiotensin-II Interacts With Nuclear Angiotensin Receptors in Cardiac Fibroblasts and Regulates RNA Synthesis, Cell Proliferation, and Collagen Secretion. J Am Heart Assoc. 2017;6(4):e004965. Published 2017 Apr 5. doi:10.1161/JAHA.116.004965.  [21] Yeh YH, Wakili R, Qi XY, et al. Calcium-handling abnormalities underlying atrial arrhythmogenesis and contractile dysfunction in dogs with congestive heart failure. Circ Arrhythm Electrophysiol. 2008;1(2):93-102. doi:10.1161/CIRCEP.107.754788.  [22] Ling LH, Khammy O, Byrne M, et al. Irregular rhythm adversely influences calcium handling in ventricular myocardium: implications for the interaction between heart failure and atrial fibrillation. Circ Heart Fail. 2012;5(6):786-793. doi:10.1161/CIRCHEARTFAILURE.112.968321.  [23] Yasushige Shingu, Suguru Kubota, Satoru Wakasa, Noriyoshi Ebuoka, Daisuke Mori, Tomonori Ooka, Tsuyoshi Tachibana, Yoshiro Matsui, Left-ventricular electromechanical delay is prolonged in patients with postoperative atrial fibrillation, European Journal of Cardio-Thoracic Surgery, Volume 39, Issue 5, May 2011, Pages 684–688, doi: 10.1016/j.ejcts.2010.08.015.  [24] Schwarzwald CC, Bonagura JD, Luis-Fuentes V. Effects of diltiazem on hemodynamic variables and ventricular function in healthy horses. J Vet Intern Med. 2005;19(5):703-711. doi:10.1892/0891-6640(2005)19[703:eodohv]2.0.co;2.  [25] Medvedowsky JL, Barnay C, Arnaud C, Bonet P, Quittet F, Lam LF. Le bépridil dans le traitement des tachycardies paroxystiques supraventriculaires [Bepridil in the treatment of supraventricular paroxysmal tachycardias]. Arch Mal Coeur Vaiss. 1985;78 Spec No:67-74. |  |
| **162 articles included atrial fibrillation but not calcium:**  [1] Canpolat U, Aytemir K, Özer N, Oto A. The impact of cryoballoon-based catheter ablation on left atrial structural and potential electrical remodeling in patients with paroxysmal atrial fibrillation. J Interv Card Electrophysiol. 2015;44(2):131-139. doi:10.1007/s10840-015-0041-1.  [2] Hori Y, Nakahara S, Anjo N, et al. Investigation of the atrial conduction time measured by tissue Doppler imaging at the left atrial appendage and the actual electrical conduction time: consideration of left atrial remodeling in atrial fibrillation patients. J Interv Card Electrophysiol. 2017;48(1):89-97. doi:10.1007/s10840-016-0185-7.  [3] Chung CC, Lin YK, Chen YC, Kao YH, Yeh YH, Chen YJ. Factor Xa inhibition by rivaroxaban regulates fibrogenesis in human atrial fibroblasts with modulation of nitric oxide synthesis and calcium homeostasis. J Mol Cell Cardiol. 2018;123:128-138. doi:10.1016/j.yjmcc.2018.09.003.  [4] Tingting H, Guangzhong L, Yanxiang Z, DongDong Y, Li S, Li W. Qiliqiangxin attenuates atrial structural remodeling in prolonged pacing-induced atrial fibrillation in rabbits. Naunyn Schmiedebergs Arch Pharmacol. 2019;392(5):585-592. doi:10.1007/s00210-018-01611-0.  [5] Niwano S, Fukaya H, Sasaki T, Hatakeyama Y, Fujiki A, Izumi T. Effect of oral L-type calcium channel blocker on repetitive paroxysmal atrial fibrillation: spectral analysis of fibrillation waves in the Holter monitoring. Europace. 2007;9(12):1209-1215. doi:10.1093/europace/eum199.  [6] Aksan G, Nar G, Soylu K, et al. Assessment of atrial electromechanical delay and left atrial mechanical functions in patients with psoriasis vulgaris. Echocardiography. 2015;32(4):615-622. doi:10.1111/echo.12706.  [7] Páll A, Czifra Á, Sebestyén V, et al. Hemodiafiltration and hemodialysis differently affect P wave duration and dispersion on the surface electrocardiogram. Int Urol Nephrol. 2016;48(2):271-277. doi:10.1007/s11255-015-1144-4.  [8] Tezcan UK, Amasyali B, Can I, et al. Increased P wave dispersion and maximum P wave duration after hemodialysis. Ann Noninvasive Electrocardiol. 2004;9(1):34-38. doi:10.1111/j.1542-474x.2004.91529.x.  [9] Rago A, Russo V, Papa AA, et al. The role of the atrial electromechanical delay in predicting atrial fibrillation in beta-thalassemia major patients. J Interv Card Electrophysiol. 2017;48(2):147-157. doi:10.1007/s10840-016-0201-y.  [10] Xi Q, Sahakian AV, Frohlich TG, Ng J, Swiryn S. Relationship between pattern of occurrence of atrial fibrillation and surface electrocardiographic fibrillatory wave characteristics. Heart Rhythm. 2004;1(6):656-663. doi:10.1016/j.hrthm.2004.09.010.  [11] Phung TN, Moyer CB, Norton PT, Ferguson JD, Holmes JW. Effect of ablation pattern on mechanical function in the atrium. Pacing Clin Electrophysiol. 2017;40(6):648-654. doi:10.1111/pace.13086.  [12] Barth AS, Merk S, Arnoldi E, et al. Reprogramming of the human atrial transcriptome in permanent atrial fibrillation: expression of a ventricular-like genomic signature. Circ Res. 2005;96(9):1022-1029. doi:10.1161/01.RES.0000165480.82737.33.  [13] Nar G, Ergul B, Aksan G, Inci S. Assessment of Atrial Electromechanical Delay and Left Atrial Mechanical Functions in Patients with Ulcerative Colitis. Echocardiography. 2016;33(7):970-976. doi:10.1111/echo.13213.  [14] Ozportakal H, Ozkok A, Alkan O, et al. Hemodialysis-induced repolarization abnormalities on ECG are influenced by serum calcium levels and ultrafiltration volumes. Int Urol Nephrol. 2017;49(3):509-515. doi: 10.1007/s11255-016-1472-z.  [15] Akyel A, Oksüz F, Karadeniz M, et al. Atrial electromechanical delay in type 2 diabetes mellitus. Wien Klin Wochenschr. 2014;126(3-4):101-105. doi: 10.1007/s00508-013-0477-1.  [16] You MJ, Langfield P, Campanari L, Dobbs M, Shrier A, Glass L. Demonstration of cardiac rotor and source mapping techniques in embryonic chick monolayers. Chaos. 2017;27(9):093938. doi:10.1063/1.5001459.  [17] Aoyama Y, Niwano S, Niwano H, et al. Repetitive evaluation of fibrillation cycle length predicts the efficacy of bepridil for interruption of long-lasting persistent atrial fibrillation. Int Heart J. 2011;52(6):353-358. doi:10.1536/ihj.52.353.  [18] Nomura M, Kawano T, Nakayasu K, Nakaya Y. The effects of losartan on signal-averaged P wave in patients with atrial fibrillation. Int J Cardiol. 2008;126(1):21-27. doi:10.1016/j.ijcard.2007.03.106.  [19] Huang F, Huang JP, Pan JY, Bai ZL, Tang L, Zhou SH. Intracellular heat shock protein 70: a possible therapeutic target for preventing postoperative atrial fibrillation. Pharmazie. 2012;67(9):747-755.  [20] Pérez Riera AR, Paixão-Almeida A, Barbosa-Barros R, et al. Congenital short QT syndrome: landmarks of the newest arrhythmogenic cardiac channelopathy. Cardiol J. 2013;20(5):464-471. doi:10.5603/CJ.a2013.0052.  [21] Chang MG, Sato D, de Lange E, et al. Bi-stable wave propagation and early afterdepolarization-mediated cardiac arrhythmias. Heart Rhythm. 2012;9(1):115-122. doi:10.1016/j.hrthm.2011.08.014.  [22] Hannes W, Fasol R, Zajonc H, et al. Diltiazem provides anti-ischemic and anti-arrhythmic protection in patients undergoing coronary bypass grafting. Eur J Cardiothorac Surg. 1993;7(5):239-245. doi: 10.1016/1010-7940(93)90211-s.  [23] Decher N, Uyguner O, Scherer CR, et al. hKChIP2 is a functional modifier of hKv4.3 potassium channels: cloning and expression of a short hKChIP2 splice variant. Cardiovasc Res. 2001;52(2):255-264. doi:10.1016/s0008-6363(01)00374-1.  [24] Seitelberger R, Hannes W, Gleichauf M, et al. Effects of diltiazem on perioperative ischemia, arrhythmias, and myocardial function in patients undergoing elective coronary bypass grafting. The Journal of Thoracic and Cardiovascular Surgery. 1994 Mar;107(3):811-821.  [25] Gonsorcík J, Palko S, Takác M, et al. Holterovo monitorovanie EKG u chorých s chronickým zlyhaním obliciek v dlhodobom dialyzacnom programe [ECG Holter monitoring in patients with chronic renal failure in a long-term dialysis program]. Vnitr Lek. 1992;38(3):258-264.  [26] Li X, Xue YM, Guo HM, et al. High hydrostatic pressure induces atrial electrical remodeling through upregulation of inflammatory cytokines. Life Sciences. 2020 Feb;242:117209. DOI: 10.1016/j.lfs.2019.117209.  [27] Ni H, Adeniran I, Zhang H. In-silico investigations of the functional impact of KCNA5 mutations on atrial mechanical dynamics. Journal of Molecular and Cellular Cardiology. 2017 Oct;111:86-95. DOI: 10.1016/j.yjmcc.2017.08.005.  [28] Morota S, Manolopoulos T, Eyjolfsson A, et al. Functional and pharmacological characteristics of permeability transition in isolated human heart mitochondria. PLoS One. 2013;8(6):e67747. Published 2013 Jun 28. doi:10.1371/journal.pone.0067747.  [29] Leboeuf J, Basiez M, Massingham R. Electrophysiological effects of Org 7797 in the closed-chest anaesthetized dog. Br J Pharmacol. 1993;110(1):23-28. doi:10.1111/j.1476-5381.1993.tb13766.x.  [30] Lu YY, Chen YC, Kao YH, Chen SA, Chen YJ. Extracellular matrix of collagen modulates arrhythmogenic activity of pulmonary veins through p38 MAPK activation. J Mol Cell Cardiol. 2013;59:159-166. doi:10.1016/j.yjmcc.2013.03.008.  [31] Ritterman JB, Hossack KF, Bruce RA. Acute and chronic effects of diltiazem on A-V conduction at rest and during exercise. J Electrocardiol. 1982;15(1):41-46. doi:10.1016/s0022-0736(82)80043-5.  [32] Guray U, Guray Y, Mecit B, Yilmaz MB, Sasmaz H, Korkmaz S. Maximum p wave duration and p wave dispersion in adult patients with secundum atrial septal defect: the impact of surgical repair. Ann Noninvasive Electrocardiol. 2004;9(2):136-141. doi:10.1111/j.1542-474X.2004.92532.x.  [33] Ingemansson MP, Carlson J, Platonov P, Olsson SB. Effects of MgSO4 and glucose, insulin and potassium (GIK) on atrial conduction during the first 12 hours after DC-conversion of chronic atrial fibrillation. Scandinavian Cardiovascular Journal: SCJ. 2001 Oct;35(5):340-346. DOI: 10.1080/140174301317116325.  [34] Oikawa J, Niwano S, Niwano H, et al. Prophylactic statin administration may prevent shortening of the fibrillation cycle length in patients with new-onset atrial fibrillation. Int Heart J. 2013;54(6):371-376. doi:10.1536/ihj.54.371.  [35] Jafari Dehkordi A, Nasser Mohebi A, Heidari Soreshjani M. Frequency of cardiac arrhythmias in high- and low- yielding dairy cows. Vet Res Forum. 2014;5(1):1-5.  [36] Etemad T, Hosseinsabet A. Determinants of left intra-atrial electromechanical delay as evaluated by tissue Doppler imaging in candidates for coronary artery bypass graft surgery. Echocardiography (Mount Kisco, N.Y.). 2020 Feb;37(2):260-269. DOI: 10.1111/echo.14599.  [37] Büttner P, Ueberham L, Shoemaker MB, et al. Identification of Central Regulators of Calcium Signaling and ECM-Receptor Interaction Genetically Associated With the Progression and Recurrence of Atrial Fibrillation. Front Genet. 2018;9:162. Published 2018 May 16. doi:10.3389/fgene.2018.00162.  [38] Cerit L, Kemal H, Gulsen K, Ozcem B, Cerit Z, Duygu H. Relationship between Vitamin D and the development of atrial fibrillation after on-pump coronary artery bypass graft surgery. Cardiovasc J Afr. 2017;28(2):104-107. doi:10.5830/CVJA-2016-064.  [39] Pedersen KB, Madsen C, Sandgaard NCF, et al. Predictive Markers of Atrial Fibrillation in Patients with Transient Ischemic Attack. J Stroke Cerebrovasc Dis. 2020;29(4):104643. doi:10.1016/j.jstrokecerebrovasdis.2020.104643.  [40] Tsadok MA, Jackevicius CA, Essebag V, et al. Rhythm versus rate control therapy and subsequent stroke or transient ischemic attack in patients with atrial fibrillation. Circulation. 2012;126(23):2680-2687. doi:10.1161/CIRCULATIONAHA.112.092494.  [41] Wettwer E, Christ T, Endig S, et al. The new antiarrhythmic drug vernakalant: ex vivo study of human atrial tissue from sinus rhythm and chronic atrial fibrillation. Cardiovasc Res. 2013;98(1):145-154. doi:10.1093/cvr/cvt006.  [42] Yao C, Veleva T, Scott L Jr, et al. Enhanced Cardiomyocyte NLRP3 Inflammasome Signaling Promotes Atrial Fibrillation [published correction appears in Circulation. 2019 Apr 23;139(17):e889]. Circulation. 2018;138(20):2227-2242. doi:10.1161/CIRCULATIONAHA.118.035202.  [43] Liu Z, Finet JE, Wolfram JA, Anderson ME, Ai X, Donahue JK. Calcium/calmodulin-dependent protein kinase II causes atrial structural remodeling associated with atrial fibrillation and heart failure. Heart Rhythm. 2019;16(7):1080-1088. doi:10.1016/j.hrthm.2019.01.013.  [44] Zou D, Geng N, Chen Y, et al. Ranolazine improves oxidative stress and mitochondrial function in the atrium of acetylcholine-CaCl2 induced atrial fibrillation rats. Life Sci. 2016;156:7-14. doi:10.1016/j.lfs.2016.05.026  [45] Sossalla S, Kallmeyer B, Wagner S, et al. Altered Na(+) currents in atrial fibrillation effects of ranolazine on arrhythmias and contractility in human atrial myocardium. J Am Coll Cardiol. 2010;55(21):2330-2342. doi:10.1016/j.jacc.2009.12.055.  [46] Feola I, Volkers L, Majumder R, et al. Localized Optogenetic Targeting of Rotors in Atrial Cardiomyocyte Monolayers. Circ Arrhythm Electrophysiol. 2017;10(11):e005591. doi:10.1161/CIRCEP.117.005591.  [47] Arora R, Aistrup GL, Supple S, et al. Regional distribution of T-tubule density in left and right atria in dogs. Heart Rhythm. 2017;14(2):273-281. doi:10.1016/j.hrthm.2016.09.022.  [48] Cunha SR, Hund TJ, Hashemi S, et al. Defects in ankyrin-based membrane protein targeting pathways underlie atrial fibrillation. Circulation. 2011;124(11):1212-1222. doi:10.1161/CIRCULATIONAHA.111.023986.  [49] Sharma AK, Kishore K, Sharma D, et al. Cardioprotective activity of alcoholic extract of Tinospora cordifolia (Willd.) Miers in calcium chloride-induced cardiac arrhythmia in rats. J Biomed Res. 2011;25(4):280-286. doi:10.1016/S1674-8301(11)60038-9.  [50] Purohit A, Rokita AG, Guan X, et al. Oxidized Ca(2+)/calmodulin-dependent protein kinase II triggers atrial fibrillation. Circulation. 2013;128(16):1748-1757. doi:10.1161/CIRCULATIONAHA.113.003313.  [51] Makara MA, Curran J, Little SC, et al. Ankyrin-G coordinates intercalated disc signaling platform to regulate cardiac excitability in vivo. Circ Res. 2014;115(11):929-938. doi:10.1161/CIRCRESAHA.115.305154.  [52] Castro L, Mialet-Perez J, Guillemeau A, et al. Differential functional effects of two 5-HT4 receptor isoforms in adult cardiomyocytes. J Mol Cell Cardiol. 2005;39(2):335-344. doi:10.1016/j.yjmcc.2005.04.009.  [53] Patel BB, Raad M, Sebag IA, Chalifour LE. Sex-specific cardiovascular responses to control or high fat diet feeding in C57bl/6 mice chronically exposed to bisphenol A. Toxicol Rep. 2015;2:1310-1318. Published 2015 Oct 1. doi:10.1016/j.toxrep.2015.09.008.  [54] Molina CE, Abu-Taha IH, Wang Q, et al. Profibrotic, Electrical, and Calcium-Handling Remodeling of the Atria in Heart Failure Patients With and Without Atrial Fibrillation. Front Physiol. 2018;9:1383. Published 2018 Oct 9. doi:10.3389/fphys.2018.01383.  [55] Hu X, Li J, van Marion DMS, Zhang D, Brundel BJJM. Heat shock protein inducer GGA*-59 reverses contractile and structural remodeling via restoration of the microtubule network in experimental Atrial Fibrillation. J Mol Cell Cardiol. 2019;134:86-97. doi:10.1016/j.yjmcc.2019.07.006.  [56] Tsai CT, Chiang FT, Tseng CD, et al. Mechanical stretch of atrial myocyte monolayer decreases sarcoplasmic reticulum calcium adenosine triphosphatase expression and increases susceptibility to repolarization alternans. Journal of the American College of Cardiology. 2011 Nov;58(20):2106-2115. DOI: 10.1016/j.jacc.2011.07.039.  [57] Chen WJ, Yeh YH, Lin KH, Chang GJ, Kuo CT. Molecular characterization of thyroid hormone-inhibited atrial L-type calcium channel expression: implication for atrial fibrillation in hyperthyroidism. Basic Res Cardiol. 2011;106(2):163-174. doi:10.1007/s00395-010-0149-5.  [58] van Marion DM, Hu X, Zhang D, et al. Screening of novel HSP-inducing compounds to conserve cardiomyocyte function in experimental atrial fibrillation. Drug Des Devel Ther. 2019;13:345-364. Published 2019 Jan 18. doi:10.2147/DDDT.S176924.  [59] Akar JG, Everett TH, Ho R, et al. Intracellular chloride accumulation and subcellular elemental distribution during atrial fibrillation. Circulation. 2003;107(13):1810-1815. doi:10.1161/01.CIR.0000058462.23347.93.  [60] Thijssen VL, Ausma J, Gorza L, et al. Troponin I isoform expression in human and experimental atrial fibrillation. Circulation. 2004;110(7):770-775. doi:10.1161/01.CIR.0000138849.03311.C6.  [61] Gong D, Zhang Y, Cai B, et al. Characterization and comparison of Na+, K+ and Ca2+ currents between myocytes from human atrial right appendage and atrial septum. Cell Physiol Biochem. 2008;21(5-6):385-394. doi:10.1159/000129631.  [62] Oh S, Kim KB, Ahn H, Cho HJ, Choi YS. Remodeling of ion channel expression in patients with chronic atrial fibrillation and mitral valvular heart disease. Korean J Intern Med. 2010;25(4):377-385. doi:10.3904/kjim.2010.25.4.377.  [63] Zhang YH, Sun HY, Chen KH, et al. Evidence for functional expression of TRPM7 channels in human atrial myocytes. Basic Res Cardiol. 2012;107(5):282. doi:10.1007/s00395-012-0282-4.  [64] Cha TJ, Ehrlich JR, Zhang L, Nattel S. Atrial ionic remodeling induced by atrial tachycardia in the presence of congestive heart failure. Circulation. 2004;110(12):1520-1526. doi:10.1161/01.CIR.0000142052.03565.87.  [65] Lin, Y., Lu, Y., Chen, Y. et al. Nitroprusside modulates pulmonary vein arrhythmogenic activity. J Biomed Sci 17, 20 (2010). doi:10.1186/1423-0127-17-20.  [66] Watanabe H, Ma M, Washizuka T, et al. Thyroid hormone regulates mRNA expression and currents of ion channels in rat atrium. Biochem Biophys Res Commun. 2003;308(3):439-444. doi:10.1016/s0006-291x(03)01420-7.  [67] Cha TJ, Ehrlich JR, Zhang L, Nattel S. Atrial ionic remodeling induced by atrial tachycardia in the presence of congestive heart failure. Circulation. 2004;110(12):1520-1526. doi:10.1161/01.CIR.0000142052.03565.87.  [68] Yu J, Li W, Li Y, et al. Activation of β(3)-adrenoceptor promotes rapid pacing-induced atrial electrical remodeling in rabbits. Cell Physiol Biochem. 2011;28(1):87-96. doi:10.1159/000331717.  [69] Yue L, Melnyk P, Gaspo R, Wang Z, Nattel S. Molecular mechanisms underlying ionic remodeling in a dog model of atrial fibrillation. Circ Res. 1999;84(7):776-784. doi:10.1161/01.res.84.7.776.  [70] Dun W, Ozgen N, Hirose M, et al. Ionic mechanisms underlying region-specific remodeling of rabbit atrial action potentials caused by intermittent burst stimulation. Heart Rhythm. 2007;4(4):499-507. doi:10.1016/j.hrthm.2006.12.032.  [71] Chen YJ, Chen YC, Yeh HI, Lin CI, Chen SA. Electrophysiology and arrhythmogenic activity of single cardiomyocytes from canine superior vena cava. Circulation. 2002;105(22):2679-2685. doi:10.1161/01.cir.0000016822.96362.26.  [72] Ehrlich JR, Cha TJ, Zhang L, et al. Cellular electrophysiology of canine pulmonary vein cardiomyocytes: action potential and ionic current properties. J Physiol. 2003;551(Pt 3):801-813. doi:10.1113/jphysiol.2003.046417.  [73] CHEN, Y.‐C. CHEN, S.‐A, CHEN, Y.‐J, TAI, C.‐T, CHAN, P. and LIN, C.‐I. (2004), T‐Type Calcium Current in Electrical Activity of Cardiomyocytes Isolated from Rabbit Pulmonary Vein. Journal of Cardiovascular Electrophysiology, 15: 567-571. doi:10.1046/j.1540-8167.2004.03399.x.  [74] Shi L, Yang XC, Liu XL, Zong M, Wu YL. Zhonghua Yi Xue Za Zhi. 2009;89(30):2142-2146.  [75] Cheng CC, Huang CF, Chen YC, et al. Heat-stress responses modulate beta-adrenergic agonist and angiotensin II effects on the arrhythmogenesis of pulmonary vein cardiomyocytes. J Cardiovasc Electrophysiol. 2011;22(2):183-190. doi:10.1111/j.1540-8167.2010.01849.x.  [76] Laszlo R, Eick C, Schwiebert M, et al. Transient outward potassium current in rabbit atrium is depressed after short-time rapid atrial pacing but recovers after a longer pacing period. Gen Physiol Biophys. 2008;27(3):174-178.  [77] Laszlo R, Bentz K, Konior A, et al. Effects of selective mineralocorticoid receptor antagonism on atrial ion currents and early ionic tachycardia-induced electrical remodelling in rabbits. Naunyn Schmiedebergs Arch Pharmacol. 2010;382(4):347-356. doi:10.1007/s00210-010-0553-2.  [78] Huang SY, Lu YY, Chen YC, et al. Hydrogen Peroxide Modulates Electrophysiological Characteristics of Left Atrial Myocytes. Acta Cardiol Sin. 2014;30(1):38-45.  [79] Chang CJ, Chen YC, Lin YK, Huang JH, Chen SA, Chen YJ. Rivaroxaban modulates electrical and mechanical characteristics of left atrium. J Biomed Sci. 2013;20(1):17. Published 2013 Mar 15. doi:10.1186/1423-0127-20-17.  [80] Workman AJ, Kane KA, Russell JA, Norrie J, Rankin AC. Chronic beta-adrenoceptor blockade and human atrial cell electrophysiology: evidence of pharmacological remodelling. Cardiovasc Res. 2003;58(3):518-525. doi:10.1016/s0008-6363(03)00263-3.  [81] Chen YJ, Chen YC, Tai CT, Yeh HI, Lin CI, Chen SA. Angiotensin II and angiotensin II receptor blocker modulate the arrhythmogenic activity of pulmonary veins. Br J Pharmacol. 2006;147(1):12-22. doi:10.1038/sj.bjp.0706445.  [82] Udyavar AR, Chen YC, Chen YJ, Cheng CC, Lin CI, Chen SA. Endothelin-1 modulates the arrhythmogenic activity of pulmonary veins. J Cardiovasc Electrophysiol. 2008;19(3):285-292. doi:10.1111/j.1540-8167.2007.01033.x.  [83] Laszlo R, Eick C, Rueb N, et al. Inhibition of the renin-angiotensin system: effects on tachycardia-induced early electrical remodelling in rabbit atrium. J Renin Angiotensin Aldosterone Syst. 2008;9(3):125-132. doi:10.1177/1470320308095262.  [84] Cheng W, Zhu Y, Wang H. The MAPK pathway is involved in the regulation of rapid pacing-induced ionic channel remodeling in rat atrial myocytes. Mol Med Rep. 2016;13(3):2677-2682. doi:10.3892/mmr.2016.4862.  [85] Suita K, Fujita T, Cai W, et al. Vidarabine, an anti-herpesvirus agent, prevents catecholamine-induced arrhythmias without adverse effect on heart function in mice. Pflugers Arch. 2018;470(6):923-935. doi:10.1007/s00424-018-2121-4.  [86] Tobelaim WS, Dvir M, Lebel G, et al. Competition of calcified calmodulin N lobe and PIP2 to an LQT mutation site in Kv7.1 channel. Proc Natl Acad Sci U S A. 2017;114(5):E869-E878. doi:10.1073/pnas.1612622114.  [87] Aistrup GL, Villuendas R, Ng J, et al. Targeted G-protein inhibition as a novel approach to decrease vagal atrial fibrillation by selective parasympathetic attenuation. Cardiovasc Res. 2009;83(3):481-492. doi:10.1093/cvr/cvp148.  [88] Xiao Z, Guo W, Sun B, et al. Enhanced Cytosolic Ca2+ Activation Underlies a Common Defect of Central Domain Cardiac Ryanodine Receptor Mutations Linked to Arrhythmias. J Biol Chem. 2016;291(47):24528-24537. doi:10.1074/jbc.M116.756528.  [89] Qi XY, Yeh YH, Xiao L, et al. Cellular signaling underlying atrial tachycardia remodeling of L-type calcium current. Circ Res. 2008;103(8):845-854. doi:10.1161/CIRCRESAHA.108.175463.  [90] Ke L, Meijering RA, Hoogstra-Berends F, et al. HSPB1, HSPB6, HSPB7 and HSPB8 protect against RhoA GTPase-induced remodeling in tachypaced atrial myocytes. PLoS One. 2011;6(6):e20395. doi:10.1371/journal.pone.0020395.  [91] Baczko I, Liknes D, Yang W, et al. Characterization of a novel multifunctional resveratrol derivative for the treatment of atrial fibrillation. Br J Pharmacol. 2014;171(1):92-106. doi:10.1111/bph.12409.  [92] Huang Y, Lu CY, Yan W, Gao L, Chen Q, Zhang YJ. Zhonghua Xin Xue Guan Bing Za Zhi. 2009;37(2):112-114..  [93] Lin YK, Chen YC, Chen YA, Yeh YH, Chen SA, Chen YJ. B-Type Natriuretic Peptide Modulates Pulmonary Vein Arrhythmogenesis: A Novel Potential Contributor to the Genesis of Atrial Tachyarrhythmia in Heart Failure. J Cardiovasc Electrophysiol. 2016;27(12):1462-1471. doi:10.1111/jce.13093.  [94] Farkasfalvi K, Stagg MA, Coppen SR, et al. Direct effects of apelin on cardiomyocyte contractility and electrophysiology. Biochem Biophys Res Commun. 2007;357(4):889-895. doi:10.1016/j.bbrc.2007.04.017.  [95] Hou JW, Li W, Fei YD, et al. ICaL and Ito mediate rate-dependent repolarization in rabbit atrial myocytes. J Physiol Biochem. 2018;74(1):57-67. doi:10.1007/s13105-017-0603-z.  [96] Salari S, Silverå Ejneby M, Brask J, Elinder F. Isopimaric acid - a multi-targeting ion channel modulator reducing excitability and arrhythmicity in a spontaneously beating mouse atrial cell line. Acta Physiol (Oxf). 2018;222(1):10.1111/apha.12895. doi:10.1111/apha.12895.  [97] Molina CE, Llach A, Herraiz-Martínez A, et al. Prevention of adenosine A2A receptor activation diminishes beat-to-beat alternation in human atrial myocytes. Basic Res Cardiol. 2016;111(1):5. doi:10.1007/s00395-015-0525-2.  [98] Laszlo R, Menzel KA, Bentz K, et al. Atorvastatin treatment affects atrial ion currents and their tachycardia-induced remodeling in rabbits. Life Sci. 2010;87(15-16):507-513. doi:10.1016/j.lfs.2010.09.010.  [99] Wang T, Huang CX, Jiang H, Tang QZ, Yang B, Li GS. Zhonghua Yi Xue Za Zhi. 2009;89(44):3138-3142.  [100] Yue L, Feng J, Gaspo R, Li GR, Wang Z, Nattel S. Ionic remodeling underlying action potential changes in a canine model of atrial fibrillation. Circ Res. 1997;81(4):512-525. doi:10.1161/01.res.81.4.512.  [101] Liang X, Xie H, Zhu PH, et al. Enhanced activity of inositol-1,4,5-trisphosphate receptors in atrial myocytes of atrial fibrillation patients. Cardiology. 2009;114(3):180-191. doi:10.1159/000228584.  [102] Du J, Xie J, Zhang Z, et al. TRPM7-mediated Ca2+ signals confer fibrogenesis in human atrial fibrillation. Circ Res. 2010;106(5):992-1003. doi:10.1161/CIRCRESAHA.109.206771.  [103] Li X, Zima AV, Sheikh F, Blatter LA, Chen J. Endothelin-1-induced arrhythmogenic Ca2+ signaling is abolished in atrial myocytes of inositol-1,4,5-trisphosphate(IP3)-receptor type 2-deficient mice. Circ Res. 2005;96(12):1274-1281. doi:10.1161/01.RES.0000172556.05576.4c.  [104] Deng C, Rao F, Wu S, et al. Pharmacological effects of carvedilol on T-type calcium current in murine HL-1 cells. Eur J Pharmacol. 2009;621(1-3):19-25. doi:10.1016/j.ejphar.2009.08.032.  [105] Adela Herraiz-Martínez, Anna Llach, Carmen Tarifa, Jorge Gandía, Verónica Jiménez-Sabado, Estefanía Lozano-Velasco, Selma A Serra, Alexander Vallmitjana, Eduardo Vázquez Ruiz de Castroviejo, Raúl Benítez, Amelia Aranega, Christian Muñoz-Guijosa, Diego Franco, Juan Cinca, Leif Hove-Madsen, The 4q25 variant rs13143308T links risk of atrial fibrillation to defective calcium homoeostasis, Cardiovascular Research, Volume 115, Issue 3, 1 March 2019, Pages 578–589. doi: 10.1093/cvr/cvy215.  [106] Tsai FC, Lin YC, Chang SH, et al. Differential left-to-right atria gene expression ratio in human sinus rhythm and atrial fibrillation: Implications for arrhythmogenesis and thrombogenesis. Int J Cardiol. 2016;222:104-112. doi:10.1016/j.ijcard.2016.07.103.  [107] Cañón S, Caballero R, Herraiz-Martínez A, et al. miR-208b upregulation interferes with calcium handling in HL-1 atrial myocytes: Implications in human chronic atrial fibrillation. J Mol Cell Cardiol. 2016;99:162-173. doi:10.1016/j.yjmcc.2016.08.012.  [108] Ni L, Scott L Jr, Campbell HM, et al. Atrial-Specific Gene Delivery Using an Adeno-Associated Viral Vector. Circ Res. 2019;124(2):256-262. doi:10.1161/CIRCRESAHA.118.313811.  [109] Chiang DY, Kongchan N, Beavers DL, et al. Loss of microRNA-106b-25 cluster promotes atrial fibrillation by enhancing ryanodine receptor type-2 expression and calcium release. Circ Arrhythm Electrophysiol. 2014;7(6):1214-1222. doi:10.1161/CIRCEP.114.001973.  [110] Zhu Y, Feng Z, Cheng W, Xiao Y. MicroRNA‑34a mediates atrial fibrillation through regulation of Ankyrin‑B expression. Mol Med Rep. 2018;17(6):8457-8465. doi:10.3892/mmr.2018.8873.  [111] Nadadur RD, Broman MT, Boukens B, et al. Pitx2 modulates a Tbx5-dependent gene regulatory network to maintain atrial rhythm. Sci Transl Med. 2016;8(354):354ra115. doi:10.1126/scitranslmed.aaf4891.  [112] Chen J, Xu S, Li W, et al. Nkx2.5 insufficiency leads to atrial electrical remodeling through Wnt signaling in HL-1 cells. Exp Ther Med. 2019;18(6):4631-4636. doi:10.3892/etm.2019.8134.  [113] Takano K, Liu D, Tarpey P, et al. An X-linked channelopathy with cardiomegaly due to a CLIC2 mutation enhancing ryanodine receptor channel activity. Hum Mol Genet. 2012;21(20):4497-4507. doi:10.1093/hmg/dds292.  [114] Kirchhof P, Kahr PC, Kaese S, et al. PITX2c is expressed in the adult left atrium, and reducing Pitx2c expression promotes atrial fibrillation inducibility and complex changes in gene expression. Circ Cardiovasc Genet. 2011;4(2):123-133. doi:10.1161/CIRCGENETICS.110.958058.  [115] Ralph F Bosch, Constanze R Scherer, Norman Rüb, et.al. Molecular mechanisms of early electrical remodeling: transcriptional downregulation of ion channel subunits reduces ICa,L and Ito in rapid atrial pacing in rabbits. Journal of the American College of Cardiology. 2003; 41(5): 858-869. doi: 10.1016/S0735-1097(02)02922-4.  [116] Gaborit N, Steenman M, Lamirault G, et al. Human atrial ion channel and transporter subunit gene-expression remodeling associated with valvular heart disease and atrial fibrillation. Circulation. 2005;112(4):471-481. doi:10.1161/CIRCULATIONAHA.104.506857.  [117] Zhang JC, Wu HL, Chen Q, et al. Calcium-Mediated Oscillation in Membrane Potentials and Atrial-Triggered Activity in Atrial Cells of Casq2R33Q/R33Q Mutation Mice. Front Physiol. 2018;9:1447. Published 2018 Nov 2. doi:10.3389/fphys.2018.01447.  [118] Zhang D, Hu X, Li J, et al. Converse role of class I and class IIa HDACs in the progression of atrial fibrillation. J Mol Cell Cardiol. 2018;125:39-49. doi:10.1016/j.yjmcc.2018.09.010.  [119] Glukhov AV, Kalyanasundaram A, Lou Q, et al. Calsequestrin 2 deletion causes sinoatrial node dysfunction and atrial arrhythmias associated with altered sarcoplasmic reticulum calcium cycling and degenerative fibrosis within the mouse atrial pacemaker complex1. Eur Heart J. 2015;36(11):686-697. doi:10.1093/eurheartj/eht452.  [120] Sood S, Chelu MG, van Oort RJ, et al. Intracellular calcium leak due to FKBP12.6 deficiency in mice facilitates the inducibility of atrial fibrillation. Heart Rhythm. 2008;5(7):1047-1054. doi:10.1016/j.hrthm.2008.03.030.  [121] Lozano-Velasco E, Hernández-Torres F, Daimi H, et al. Pitx2 impairs calcium handling in a dose-dependent manner by modulating Wnt signalling. Cardiovasc Res. 2016;109(1):55-66. doi:10.1093/cvr/cvv207.  [122] Lou Q, Belevych AE, Radwański PB, et al. Alternating membrane potential/calcium interplay underlies repetitive focal activity in a genetic model of calcium-dependent atrial arrhythmias. J Physiol. 2015;593(6):1443-1458. doi:10.1113/jphysiol.2014.280784.  [123] Bögeholz N, Pauls P, Kaese S, et al. Triggered activity in atrial myocytes is influenced by Na+/Ca2+ exchanger activity in genetically altered mice. J Mol Cell Cardiol. 2016;101:106-115. doi:10.1016/j.yjmcc.2016.11.004.  [124] Zhang JC, Wu HL, Chen Q, et al. Calcium-Mediated Oscillation in Membrane Potentials and Atrial-Triggered Activity in Atrial Cells of Casq2R33Q/R33Q Mutation Mice. Front Physiol. 2018;9:1447. Published 2018 Nov 2. doi:10.3389/fphys.2018.01447.  [125] Tao Y, Zhang M, Li L, et al. Pitx2, an atrial fibrillation predisposition gene, directly regulates ion transport and intercalated disc genes. Circ Cardiovasc Genet. 2014;7(1):23-32. doi:10.1161/CIRCGENETICS.113.000259.  [126] Hong, Chang-Soo & Kwon, Soon-Jae & Cho, Myeong-Chan & Kwak, et. al. Overexpression of junctate induces cardiac hypertrophy and arrhythmia via altered calcium handling. Journal of molecular and cellular cardiology. 2008; 44: 672-82. 10.1016/j.yjmcc.2008.01.012.  [127] Kao YH, Hsu JC, Chen YC, et al. ZFHX3 knockdown increases arrhythmogenesis and dysregulates calcium homeostasis in HL-1 atrial myocytes. Int J Cardiol. 2016;210:85-92. doi:10.1016/j.ijcard.2016.02.091.  [128] Wiersma M, Meijering RAM, Qi XY, et al. Endoplasmic Reticulum Stress Is Associated With Autophagy and Cardiomyocyte Remodeling in Experimental and Human Atrial Fibrillation. J Am Heart Assoc. 2017;6(10):e006458. Published 2017 Oct 24. doi:10.1161/JAHA.117.006458.  [129] DeSantiago J, Bare DJ, Varma D, Solaro RJ, Arora R, Banach K. Loss of p21-activated kinase 1 (Pak1) promotes atrial arrhythmic activity. Heart Rhythm. 2018;15(8):1233-1241. doi:10.1016/j.hrthm.2018.03.041.  [130] Liu CH, Hua N, Fu X, Pan YL, Li B, Li XD. Metformin regulates atrial SK2 and SK3 expression through inhibiting the PKC/ERK signaling pathway in type 2 diabetic rats. BMC Cardiovasc Disord. 2018;18(1):236. Published 2018 Dec 13. doi:10.1186/s12872-018-0950-x.  [131] Sun J, Ailiman M. Regulation of calcium pump through Notch/Jagged/Hes signaling pathway in canine model of chronic atrial fibrillation. Int J Clin Exp Pathol. 2019;12(11):4034-4040. Published 2019 Nov 1.  [132] Bukowska A, Lendeckel U, Hirte D, et al. Activation of the calcineurin signaling pathway induces atrial hypertrophy during atrial fibrillation. Cell Mol Life Sci. 2006;63(3):333-342. doi:10.1007/s00018-005-5353-3.  [133] Yeh YH, Kuo CT, Chang GJ, et al. Rosuvastatin suppresses atrial tachycardia-induced cellular remodeling via Akt/Nrf2/heme oxygenase-1 pathway. J Mol Cell Cardiol. 2015;82:84-92. doi:10.1016/j.yjmcc.2015.03.004.  [134] Wang W, Zhu Y, Yi J, Cheng W. Nkx2.5/CARP signaling pathway contributes to the regulation of ion channel remodeling induced by rapid pacing in rat atrial myocytes. Mol Med Rep. 2016;14(4):3848-3854. doi:10.3892/mmr.2016.5727.  [135] Tang M, Yuan W, Fan X, et al. Pygopus maintains heart function in aging Drosophila independently of canonical Wnt signaling. Circ Cardiovasc Genet. 2013;6(5):472-480. doi:10.1161/CIRCGENETICS.113.000253.  [135] Chen PS, Chen LS, Fishbein MC, Lin SF, Nattel S. Role of the autonomic nervous system in atrial fibrillation: pathophysiology and therapy. Circ Res. 2014;114(9):1500-1515. doi:10.1161/CIRCRESAHA.114.303772.  [136] Zhang H, Bryson V, Luo N, Sun AY, Rosenberg P. STIM1-Ca2+ signaling in coronary sinus cardiomyocytes contributes to interatrial conduction. Cell Calcium. 2020;87:102163. doi:10.1016/j.ceca.2020.102163.  [137] Yin D, Chen M, Yang N, et al. Role of apamin-sensitive small conductance calcium-activated potassium currents in long-term cardiac memory in rabbits. Heart Rhythm. 2018;15(5):761-769. doi:10.1016/j.hrthm.2018.01.016.  [138] Hsueh CH, Chang PC, Hsieh YC, Reher T, Chen PS, Lin SF. Proarrhythmic effect of blocking the small conductance calcium activated potassium channel in isolated canine left atrium. Heart Rhythm. 2013;10(6):891-898. doi:10.1016/j.hrthm.2013.01.033.  [139] Jannie Ausma, Gerrit D Dispersyn, Hans Duimel, et. al. Changes in Ultrastructural Calcium Distribution in Goat Atria During Atrial Fibrillation. Journal of Molecular and Cellular Cardiology. 2000; 32(3): 355-364,doi: 10.1006/jmcc.1999.1090.  [140] Ono N, Hayashi H, Kawase A, et al. Spontaneous atrial fibrillation initiated by triggered activity near the pulmonary veins in aged rats subjected to glycolytic inhibition. Am J Physiol Heart Circ Physiol. 2007;292(1):H639-H648. doi:10.1152/ajpheart.00445.2006.  [141] Schreieck J, Wang Y, Overbeck M, Schömig A, Schmitt C. Altered transient outward current in human atrial myocytes of patients with reduced left ventricular function. J Cardiovasc Electrophysiol. 2000;11(2):180-192. doi:10.1111/j.1540-8167.2000.tb00318.x.  [142] Lerman BB, Ellenbogen KA, Kadish A, et al. Electrophysiologic effects of a novel selective adenosine A1 agonist (CVT-510) on atrioventricular nodal conduction in humans. J Cardiovasc Pharmacol Ther. 2001;6(3):237-245. doi:10.1177/107424840100600304.  [143] Kebbati A. Hafid, Huang Cong Xin, Wang Xi, Zhao Qing Yan, Yang Bo. Difference between electrical remodelling after pulmonary veins and right atrium appendage pacing. EP Europace. 2007; 9(8): 608–612.  [144] Workman AJ, Pau D, Redpath CJ, et al. Atrial cellular electrophysiological changes in patients with ventricular dysfunction may predispose to AF. Heart Rhythm. 2009;6(4):445-451. doi:10.1016/j.hrthm.2008.12.028.  [145] Hirose M, Laurita KR. Calcium-mediated triggered activity is an underlying cellular mechanism of ectopy originating from the pulmonary vein in dogs. Am J Physiol Heart Circ Physiol. 2007;292(4):H1861-H1867. doi:10.1152/ajpheart.00826.2006.  [146] Yan J, Zhao W, Thomson JK, et al. Stress Signaling JNK2 Crosstalk With CaMKII Underlies Enhanced Atrial Arrhythmogenesis. Circ Res. 2018;122(6):821-835. doi:10.1161/CIRCRESAHA.117.312536.  [147] Lin Y, Yang B, Garcia FC, et al. Comparison of left atrial electrophysiologic abnormalities during sinus rhythm in patients with different type of atrial fibrillation. J Interv Card Electrophysiol. 2014;39(1):57-67. doi:10.1007/s10840-013-9838-y.  [148] O'Connell RP, Musa H, Gomez MS, et al. Free Fatty Acid Effects on the Atrial Myocardium: Membrane Ionic Currents Are Remodeled by the Disruption of T-Tubular Architecture. PLoS One. 2015;10(8):e0133052. Published 2015 Aug 14. doi:10.1371/journal.pone.0133052.  [149] Honjo H, Boyett MR, Niwa R, et al. Pacing-induced spontaneous activity in myocardial sleeves of pulmonary veins after treatment with ryanodine. Circulation. 2003;107(14):1937-1943. doi:10.1161/01.CIR.0000062645.38670.BD.  [150] Rietdorf K, Bootman MD, Sanderson MJ. Spontaneous, pro-arrhythmic calcium signals disrupt electrical pacing in mouse pulmonary vein sleeve cells. PLoS One. 2014;9(2):e88649. Published 2014 Feb 20. doi:10.1371/journal.pone.0088649.  [151] Nakagawa H, Scherlag BJ, Patterson E, Ikeda A, Lockwood D, Jackman WM. Pathophysiologic basis of autonomic ganglionated plexus ablation in patients with atrial fibrillation. Heart Rhythm. 2009;6(12 Suppl):S26-S34. doi:10.1016/j.hrthm.2009.07.029.  [152]Bukowska A, Schild L, Keilhoff G, et al. Mitochondrial dysfunction and redox signaling in atrial tachyarrhythmia. Exp Biol Med (Maywood). 2008;233(5):558-574. doi:10.3181/0706-RM-155.  [153] Numata A, Miyauchi Y, Ono N, et al. Spontaneous atrial fibrillation initiated by tyramine in canine atria with increased sympathetic nerve sprouting. J Cardiovasc Electrophysiol. 2012;23(4):415-422. doi:10.1111/j.1540-8167.2011.02197.x.  [154] Choi EK, Chang PC, Lee YS, et al. Triggered firing and atrial fibrillation in transgenic mice with selective atrial fibrosis induced by overexpression of TGF-β1. Circ J. 2012;76(6):1354-1362. doi:10.1253/circj.cj-11-1301.  [155] Kuijpers NH, Potse M, van Dam PM, et al. Mechanoelectrical coupling enhances initiation and affects perpetuation of atrial fibrillation during acute atrial dilation. Heart Rhythm. 2011;8(3):429-436. doi:10.1016/j.hrthm.2010.11.020.  [156] Suita K, Fujita T, Hasegawa N, et al. Norepinephrine-Induced Adrenergic Activation Strikingly Increased the Atrial Fibrillation Duration through β1- and α1-Adrenergic Receptor-Mediated Signaling in Mice. PLoS One. 2015;10(7):e0133664. Published 2015 Jul 23. doi:10.1371/journal.pone.0133664.  [157] Prajapati R, Fujita T, Suita K, et al. Usefulness of Exchanged Protein Directly Activated by cAMP (Epac)1-Inhibiting Therapy for Prevention of Atrial and Ventricular Arrhythmias in Mice. Circ J. 2019;83(2):295-303. doi:10.1253/circj.CJ-18-0743.  [158] Zhao ZH, Zhang HC, Xu Y, et al. Inositol-1,4,5-trisphosphate and ryanodine-dependent Ca2+ signaling in a chronic dog model of atrial fibrillation. Cardiology. 2007;107(4):269-276. doi:10.1159/000095517.  [159] Huang CF, Chen YC, Yeh HI, Chen SA. Mononucleated and binucleated cardiomyocytes in left atrium and pulmonary vein have different electrical activity and calcium dynamics. Prog Biophys Mol Biol. 2012;108(1-2):64-73. doi:10.1016/j.pbiomolbio.2011.09.007.  [160] Baczko I, Liknes D, Yang W, et al. Characterization of a novel multifunctional resveratrol derivative for the treatment of atrial fibrillation. Br J Pharmacol. 2014;171(1):92-106. doi:10.1111/bph.12409.  [161] Husser D, Büttner P, Ueberham L, et al. Genomic Contributors to Rhythm Outcome of Atrial Fibrillation Catheter Ablation - Pathway Enrichment Analysis of GWAS Data. PLoS One. 2016;11(11):e0167008. Published 2016 Nov 21. doi:10.1371/journal.pone.0167008.  [162] Barana A, Matamoros M, Dolz-Gaitón P, et al. Chronic atrial fibrillation increases microRNA-21 in human atrial myocytes decreasing L-type calcium current. Circ Arrhythm Electrophysiol. 2014;7(5):861-868. doi:10.1161/CIRCEP.114.001709. |  |
| **135 papers were review articles:**  [1] Denham NC, Pearman CM, Caldwell JL, et al. Calcium in the Pathophysiology of Atrial Fibrillation and Heart Failure. Front Physiol. 2018;9:1380. Published 2018 Oct 4. doi:10.3389/fphys.2018.01380.  [2] Jalife J, Kaur K. Atrial remodeling, fibrosis, and atrial fibrillation. Trends Cardiovasc Med. 2015;25(6):475-484. doi:10.1016/j.tcm.2014.12.015.  [3] Stephan LS, Almeida ED, Markoski MM, Garavaglia J, Marcadenti A. Red Wine, Resveratrol and Atrial Fibrillation. Nutrients. 2017;9(11):1190. Published 2017 Oct 30. doi:10.3390/nu9111190.  [4] Andrade J, Khairy P, Dobrev D, Nattel S. The clinical profile and pathophysiology of atrial fibrillation: relationships among clinical features, epidemiology, and mechanisms. Circ Res. 2014;114(9):1453-1468. doi:10.1161/CIRCRESAHA.114.303211.  [5] Heijman J, Voigt N, Nattel S, Dobrev D. Cellular and molecular electrophysiology of atrial fibrillation initiation, maintenance, and progression. Circ Res. 2014;114(9):1483-1499. doi:10.1161/CIRCRESAHA.114.302226.  [6] Nattel S, Harada M. Atrial remodeling and atrial fibrillation: recent advances and translational perspectives. J Am Coll Cardiol. 2014;63(22):2335-2345. doi:10.1016/j.jacc.2014.02.555.  [7] Smith GL, Eisner DA. Calcium Buffering in the Heart in Health and Disease. Circulation. 2019;139(20):2358-2371. doi:10.1161/CIRCULATIONAHA.118.039329.  [8] Greiser M. Calcium signalling silencing in atrial fibrillation. J Physiol. 2017;595(12):4009-4017. doi:10.1113/JP273045.  [9] Van Wagoner DR. Electrophysiological remodeling in human atrial fibrillation. Pacing Clin Electrophysiol. 2003;26(7 Pt 2):1572-1575. doi:10.1046/j.1460-9592.2003.t01-1-00234.x.  [10] Ai X. SR calcium handling dysfunction, stress-response signaling pathways, and atrial fibrillation. Front Physiol. 2015;6:46. Published 2015 Feb 19. doi:10.3389/fphys.2015.00046.  [11] Bers DM. Stabilizing ryanodine receptor gating quiets arrhythmogenic events in human heart failure and atrial fibrillation. Heart Rhythm. 2017;14(3):420-421. doi:10.1016/j.hrthm.2016.10.005.  [12] Dobrev D. Unique cardiomyocyte ultrastructure in atria: Role of T tubules in subcellular Ca2+ signaling and atrial arrhythmogenesis. Heart Rhythm. 2017;14(2):282-283. doi:10.1016/j.hrthm.2016.10.013.  [13] Blatter LA, Kockskämper J, Sheehan KA, Zima AV, Hüser J, Lipsius SL. Local calcium gradients during excitation-contraction coupling and alternans in atrial myocytes. J Physiol. 2003;546(Pt 1):19-31. doi:10.1113/jphysiol.2002.025239.  [14] Trafford AW, Clarke JD, Richards MA, Eisner DA, Dibb KM. Calcium signalling microdomains and the t-tubular system in atrial mycoytes: potential roles in cardiac disease and arrhythmias. Cardiovasc Res. 2013;98(2):192-203. doi:10.1093/cvr/cvt018.  [15] Voigt N, Dobrev D. Cellular and molecular correlates of ectopic activity in patients with atrial fibrillation. Europace. 2012;14 Suppl 5:v97-v105. doi:10.1093/europace/eus282.  [16] Dobrev D. Atrial Ca2+ signaling in atrial fibrillation as an antiarrhythmic drug target. Naunyn Schmiedebergs Arch Pharmacol. 2010;381(3):195-206. doi:10.1007/s00210-009-0457-1.  [17] Greiser M, Schotten U. Dynamic remodeling of intracellular Ca²⁺ signaling during atrial fibrillation. J Mol Cell Cardiol. 2013;58:134-142. doi:10.1016/j.yjmcc.2012.12.020.  [18] Rietdorf K, Masoud S, McDonald F, Sanderson MJ, Bootman MD. Pulmonary vein sleeve cell excitation-contraction-coupling becomes dysynchronized by spontaneous calcium transients. Biochem Soc Trans. 2015;43(3):410-416. doi:10.1042/BST20140299.  [19] Luczak ED, Anderson ME. CaMKII oxidative activation and the pathogenesis of cardiac disease. J Mol Cell Cardiol. 2014;73:112-116. doi:10.1016/j.yjmcc.2014.02.004.  [20] Kim GH. MicroRNA regulation of cardiac conduction and arrhythmias. Transl Res. 2013;161(5):381-392. doi:10.1016/j.trsl.2012.12.004.  [21] Rottbauer W, Baker K, Wo ZG, Mohideen MA, Cantiello HF, Fishman MC. Growth and function of the embryonic heart depend upon the cardiac-specific L-type calcium channel alpha1 subunit. Dev Cell. 2001;1(2):265-275. doi:10.1016/s1534-5807(01)00023-5.  [22] Wan E, Abrams J, Weinberg RL, et al. Aberrant sodium influx causes cardiomyopathy and atrial fibrillation in mice. J Clin Invest. 2016;126(1):112-122. doi:10.1172/JCI84669.  [23] Pandit SV, Workman AJ. Atrial Electrophysiological Remodeling and Fibrillation in Heart Failure. Clin Med Insights Cardiol. 2016;10(Suppl 1):41-46. Published 2016 Oct 31. doi:10.4137/CMC.S39713.  [24] Landstrom AP, Dobrev D, Wehrens XHT. Calcium Signaling and Cardiac Arrhythmias. Circ Res. 2017;120(12):1969-1993. doi:10.1161/CIRCRESAHA.117.310083.  [25] Heijman J, Voigt N, Ghezelbash S, Schirmer I, Dobrev D. Calcium Handling Abnormalities as a Target for Atrial Fibrillation Therapeutics: How Close to Clinical Implementation?. J Cardiovasc Pharmacol. 2015;66(6):515-522. doi:10.1097/FJC.0000000000000253.  [26] Husser D, Ueberham L, Dinov B, et al. Genomic contributors to atrial electroanatomical remodeling and atrial fibrillation progression: Pathway enrichment analysis of GWAS data. Sci Rep. 2016;6:36630. Published 2016 Nov 18. doi:10.1038/srep36630.  [27] Chang, K., Trayanova, N. Mechanisms of arrhythmogenesis related to calcium-driven alternans in a model of human atrial fibrillation. Sci Rep 6, 36395 (2016). doi: 10.1038/srep36395.  [28] Nattel S, Dobrev D. Deciphering the fundamental mechanisms of atrial fibrillation: a quest for over a century. Cardiovasc Res. 2016;109(4):465-466. doi:10.1093/cvr/cvw028.  [29] Hamilton S, Terentyev D. Altered Intracellular Calcium Homeostasis and Arrhythmogenesis in the Aged Heart. Int J Mol Sci. 2019;20(10):2386. Published 2019 May 14. doi:10.3390/ijms20102386.  [30] Weirich J. Remodeling des Altersherzens : Sinusknotendysfunktion und Vorhofflimmern [Remodeling of the aging heart : Sinus node dysfunction and atrial fibrillation]. Herzschrittmacherther Elektrophysiol. 2017;28(1):29-38. doi:10.1007/s00399-017-0485-3.  [31] Hegyi B, Bers DM, Bossuyt J. CaMKII signaling in heart diseases: Emerging role in diabetic cardiomyopathy. J Mol Cell Cardiol. 2019;127:246-259. doi:10.1016/j.yjmcc.2019.01.001.  [32] Gomez-Hurtado N, Knollmann BC. Calcium in atrial fibrillation - pulling the trigger or not?. J Clin Invest. 2014;124(11):4684-4686. doi:10.1172/JCI77986.  [33] Shiferaw Y, Aistrup GL, Wasserstrom JA. Mechanism for Triggered Waves in Atrial Myocytes. Biophys J. 2017;113(3):656-670. doi:10.1016/j.bpj.2017.06.026.  [34] Shiferaw Y, Aistrup GL, Wasserstrom JA. Synchronization of Triggered Waves in Atrial Tissue. Biophys J. 2018;115(6):1130-1141. doi:10.1016/j.bpj.2018.08.015.  [35] Yoo S, Aistrup G, Shiferaw Y, et al. Oxidative stress creates a unique, CaMKII-mediated substrate for atrial fibrillation in heart failure. JCI Insight. 2018;3(21):e120728. Published 2018 Nov 2. doi:10.1172/jci.insight.120728.  [36] Wijesurendra RS, Casadei B. Atrial fibrillation: effects beyond the atrium?. Cardiovasc Res. 2015;105(3):238-247. doi:10.1093/cvr/cvv001.  [37] Schotten U, Dobrev D, Platonov PG, Kottkamp H, Hindricks G. Current controversies in determining the main mechanisms of atrial fibrillation. J Intern Med. 2016;279(5):428-438. doi:10.1111/joim.12492.  [38] Brandenburg S, Arakel EC, Schwappach B, Lehnart SE. The molecular and functional identities of atrial cardiomyocytes in health and disease. Biochim Biophys Acta. 2016;1863(7 Pt B):1882-1893. doi:10.1016/j.bbamcr.2015.11.025.  [39] Scott L Jr, Li N, Dobrev D. Role of inflammatory signaling in atrial fibrillation. Int J Cardiol. 2019;287:195-200. doi:10.1016/j.ijcard.2018.10.020.  [40] Ninio DM, Saint DA. The role of stretch-activated channels in atrial fibrillation and the impact of intracellular acidosis. Prog Biophys Mol Biol. 2008;97(2-3):401-416. doi:10.1016/j.pbiomolbio.2008.02.016.  [41] Brocklehurst P, Ni H, Zhang H, Ye J. Electro-mechanical dynamics of spiral waves in a discrete 2D model of human atrial tissue. PLoS One. 2017;12(5):e0176607. Published 2017 May 16. doi:10.1371/journal.pone.0176607.  [42] Chen PS, Tan AY. Autonomic nerve activity and atrial fibrillation. Heart Rhythm. 2007;4(3 Suppl):S61-S64. doi:10.1016/j.hrthm.2006.12.006.  [43] Kettlewell S, Burton FL, Smith GL, Workman AJ. Chronic myocardial infarction promotes atrial action potential alternans, afterdepolarizations, and fibrillation. Cardiovasc Res. 2013;99(1):215-224. doi:10.1093/cvr/cvt087.  [44] Donahue JK. Biological Therapies for Atrial Fibrillation: Ready for Prime Time?. J Cardiovasc Pharmacol. 2016;67(1):19-25. doi:10.1097/FJC.0000000000000293.  [45] Colman MA. Arrhythmia mechanisms and spontaneous calcium release: Bi-directional coupling between re-entrant and focal excitation. PLoS Comput Biol. 2019;15(8):e1007260. Published 2019 Aug 8. doi:10.1371/journal.pcbi.1007260.  [46] Workman AJ. Cardiac adrenergic control and atrial fibrillation. Naunyn Schmiedebergs Arch Pharmacol. 2010;381(3):235-249. doi:10.1007/s00210-009-0474-0.  [47] Dobrev D, Voigt N, Wehrens XH. The ryanodine receptor channel as a molecular motif in atrial fibrillation: pathophysiological and therapeutic implications. Cardiovasc Res. 2011;89(4):734-743. doi:10.1093/cvr/cvq324.  [48] Dobrev D, Wehrens XH. Calmodulin kinase II, sarcoplasmic reticulum Ca2+ leak, and atrial fibrillation. Trends Cardiovasc Med. 2010;20(1):30-34. doi:10.1016/j.tcm.2010.03.004.  [49] Denham Nathan C., Pearman Charles M., Caldwell Jessica L., et. al. Calcium in the Pathophysiology of Atrial Fibrillation and Heart Failure. Front. Physiol. 2018. doi: 10.3389/fphys.2018.01380.  [50] Van Wagoner, D. R., Pond, A. L., Lamorgese, M., Rossie, S. S., McCarthy, P. M., & Nerbonne, J. M. (1999). Atrial L-type Ca2+ currents and human atrial fibrillation. Circulation research, 85(5), 428-436. doi: 10.1161/01.RES.85.5.428.  [51] Sridhar A, Nishijima Y, Terentyev D, et al. Chronic heart failure and the substrate for atrial fibrillation. Cardiovasc Res. 2009;84(2):227-236. doi:10.1093/cvr/cvp216.  [52] Yue L, Xie J, Nattel S. Molecular determinants of cardiac fibroblast electrical function and therapeutic implications for atrial fibrillation. Cardiovasc Res. 2011;89(4):744-753. doi:10.1093/cvr/cvq329.  [53] Schönleitner P, Schotten U, Antoons G. Mechanosensitivity of microdomain calcium signalling in the heart. Prog Biophys Mol Biol. 2017;130(Pt B):288-301. doi:10.1016/j.pbiomolbio.2017.06.013.  [54] Choi E, Cha MJ, Hwang KC. Roles of Calcium Regulating MicroRNAs in Cardiac Ischemia-Reperfusion Injury. Cells. 2014;3(3):899-913. Published 2014 Sep 11. doi:10.3390/cells3030899.  [55] Harada M, Luo X, Murohara T, Yang B, Dobrev D, Nattel S. MicroRNA regulation and cardiac calcium signaling: role in cardiac disease and therapeutic potential. Circ Res. 2014;114(4):689-705. doi:10.1161/CIRCRESAHA.114.301798.  [56] Dobrev D. 5-hydroxytryptamine and atrial arrhythmogenesis: a "culprit mechanism" or bystander in patients with chronic atrial fibrillation?. J Mol Cell Cardiol. 2007;42(1):51-53. doi:10.1016/j.yjmcc.2006.09.014.  [57] Kanaporis G, Blatter LA. Alternans in atria: Mechanisms and clinical relevance. Medicina (Kaunas). 2017;53(3):139-149. doi:10.1016/j.medici.2017.04.004.  [58] Bootman MD, Smyrnias I, Thul R, Coombes S, Roderick HL. Atrial cardiomyocyte calcium signalling. Biochim Biophys Acta. 2011;1813(5):922-934. doi:10.1016/j.bbamcr.2011.01.030.  [59] Whittaker DG, Ni H, El Harchi A, Hancox JC, Zhang H. Atrial arrhythmogenicity of KCNJ2 mutations in short QT syndrome: Insights from virtual human atria [published correction appears in PLoS Comput Biol. 2019 Jun 13;15(6):e1007145]. PLoS Comput Biol. 2017;13(6):e1005593. Published 2017 Jun 13. doi:10.1371/journal.pcbi.1005593.  [60] Goyal R, Singh A, Fan R. Not Your Usual Pre-Excitation. Circulation. 2017;135(18):1759-1761. doi:10.1161/CIRCULATIONAHA.117.028386.  [61] Tanaka H. Nihon Yakurigaku Zasshi. 2019;154(4):171-177. doi:10.1254/fpj.154.171.  [62] Ripplinger CM. A leap(frog) forward in understanding focal arrhythmia. J Physiol. 2015;593(6):1383-1384. doi:10.1113/jphysiol.2014.287128.  [63] Voigt N, Nattel S, Dobrev D. Proarrhythmic atrial calcium cycling in the diseased heart. Adv Exp Med Biol. 2012;740:1175-1191. doi:10.1007/978-94-007-2888-2_53.  [64] Joung B, Chen PS. Function and dysfunction of human sinoatrial node. Korean Circ J. 2015;45(3):184-191. doi:10.4070/kcj.2015.45.3.184.  [65] Zhu H, Xue H, Jin QH, Guo J, Chen YD. Increased expression of ryanodine receptor type-2 during atrial fibrillation by miR-106-25 cluster independent mechanism. Exp Cell Res. 2019;375(2):113-117. doi:10.1016/j.yexcr.2018.11.025.  [66] Li Q, O'Neill SC, Tao T, Li Y, Eisner D, Zhang H. Mechanisms by which cytoplasmic calcium wave propagation and alternans are generated in cardiac atrial myocytes lacking T-tubules-insights from a simulation study. Biophys J. 2012;102(7):1471-1482. doi:10.1016/j.bpj.2012.03.007.  [67] Boulkroun S, Fernandes-Rosa FL, Zennaro MC. Molecular and Cellular Mechanisms of Aldosterone Producing Adenoma Development. Front Endocrinol (Lausanne). 2015;6:95. Published 2015 Jun 11. doi:10.3389/fendo.2015.00095.  [68] Nattel S. Ionic determinants of atrial fibrillation and Ca2+ channel abnormalities : cause, consequence, or innocent bystander?. Circ Res. 1999;85(5):473-476. doi:10.1161/01.res.85.5.473.  [69] Rankin AC, Workman AJ. Duration of heart failure and the risk of atrial fibrillation: different mechanisms at different times?. Cardiovasc Res. 2009;84(2):180-181. doi:10.1093/cvr/cvp299.  [70] Hatem S. Mécanismes moléculaires et cellulaires de la fibrillation auriculaire : existe-t-il de nouvelles stratégies thérapeutiques ? [Biology of the substrate of atrial fibrillation]. Biol Aujourdhui. 2012;206(1):5-9. doi:10.1051/jbio/2012004.  [71] Simon JN, Duglan D, Casadei B, Carnicer R. Nitric oxide synthase regulation of cardiac excitation-contraction coupling in health and disease. J Mol Cell Cardiol. 2014;73:80-91. doi:10.1016/j.yjmcc.2014.03.004.  [72] Carnicer R, Crabtree MJ, Sivakumaran V, Casadei B, Kass DA. Nitric oxide synthases in heart failure. Antioxid Redox Signal. 2013;18(9):1078-1099. doi:10.1089/ars.2012.4824.  [73] Dun W, Boyden PA. Aged atria: electrical remodeling conducive to atrial fibrillation. J Interv Card Electrophysiol. 2009;25(1):9-18. doi:10.1007/s10840-008-9358-3.  [74] Seferović PM, Ristić AD, Maksimović R, et al. Cardiac arrhythmias and conduction disturbances in autoimmune rheumatic diseases. Rheumatology (Oxford). 2006;45 Suppl 4:iv39-iv42. doi:10.1093/rheumatology/kel315.  [75] Umehara S, Tan X, Okamoto Y, et al. Mechanisms Underlying Spontaneous Action Potential Generation Induced by Catecholamine in Pulmonary Vein Cardiomyocytes: A Simulation Study. Int J Mol Sci. 2019;20(12):2913. Published 2019 Jun 14. doi:10.3390/ijms20122913.  [76] Kaneko N, Matsuda R, Hata Y, Shimamoto K. Pharmacological characteristics and clinical applications of K201. Curr Clin Pharmacol. 2009;4(2):126-131. doi:10.2174/157488409788184972.  [77] Ferrantini C, Crocini C, Coppini R, et al. The transverse-axial tubular system of cardiomyocytes. Cell Mol Life Sci. 2013;70(24):4695-4710. doi:10.1007/s00018-013-1410-5.  [78] Gonzalez DR, Treuer A, Sun QA, Stamler JS, Hare JM. S-Nitrosylation of cardiac ion channels. J Cardiovasc Pharmacol. 2009;54(3):188-195. doi:10.1097/FJC.0b013e3181b72c9f.  [79] Tanaka H, Matsuyama TA, Takamatsu T. Towards an integrated understanding of cardiac arrhythmogenesis - Growing roles of experimental pathology. Pathol Int. 2017;67(1):8-16. doi:10.1111/pin.12487.  [80] Chen PS, Joung B, Shinohara T, Das M, Chen Z, Lin SF. The initiation of the heart beat. Circ J. 2010;74(2):221-225. doi:10.1253/circj.cj-09-0712.  [81] Burashnikov A, Antzelevitch C. New developments in atrial antiarrhythmic drug therapy. Nat Rev Cardiol. 2010;7(3):139-148. doi:10.1038/nrcardio.2009.245.  [82] Anumonwo JM. Activation of I sac promotes atrial fibrillation initiation and perpetuation: is this a stretch?. Heart Rhythm. 2011;8(3):437-438. doi:10.1016/j.hrthm.2010.12.020.  [83] Xi Y, Cheng J. Dysfunction of the autonomic nervous system in atrial fibrillation. J Thorac Dis. 2015;7(2):193-198. doi:10.3978/j.issn.2072-1439.2015.01.12.  [84] Louch WE, Sejersted OM, Swift F. There goes the neighborhood: pathological alterations in T-tubule morphology and consequences for cardiomyocyte Ca2+ handling. J Biomed Biotechnol. 2010;2010:503906. doi:10.1155/2010/503906.  [85] Remo BF, Fishman GI. A colorful explanation for atrial arrhythmias. Pigment Cell Melanoma Res. 2010;23(1):3-4. doi:10.1111/j.1755-148X.2009.00658.x.  [86] Dillmann W. Cardiac hypertrophy and thyroid hormone signaling. Heart Fail Rev. 2010;15(2):125-132. doi:10.1007/s10741-008-9125-7.  [87] Marsh JD, Telemaque S, Rhee SW, Stimers JR, Rusch NJ. Delivery of ion channel genes to treat cardiovascular diseases. Trans Am Clin Climatol Assoc. 2008;119:171-183.  [88] Gheorghiade M, Adams KF Jr, Colucci WS. Digoxin in the management of cardiovascular disorders. Circulation. 2004;109(24):2959-2964. doi:10.1161/01.CIR.0000132482.95686.87.  [89] Zicha S, Tsuji Y, Shiroshita-Takeshita A, Nattel S. Beta-blockers as antiarrhythmic agents. Handb Exp Pharmacol. 2006;(171):235-266.  [90] Thodeti CK, Paruchuri S, Meszaros JG. A TRP to cardiac fibroblast differentiation. Channels (Austin). 2013;7(3):211-214. doi:10.4161/chan.24328.  [91] Ochi R, Gupte SA. Ryanodine receptor: a novel therapeutic target in heart disease. Recent Pat Cardiovasc Drug Discov. 2007;2(2):110-118. doi:10.2174/157489007780832524.  [92] Bollmann A, Husser D, Stridh M, et al. Atrial fibrillatory rate and risk of left atrial thrombus in atrial fibrillation. Europace. 2007;9(8):621-626. doi:10.1093/europace/eum125.  [93] Schwartz M, Rodman D, Lowenstein SR. Recognition and treatment of multifocal atrial tachycardia: a critical review. J Emerg Med. 1994;12(3):353-360. doi:10.1016/0736-4679(94)90278-x.  [94] Kanaporis G, Blatter LA. The mechanisms of calcium cycling and action potential dynamics in cardiac alternans. Circ Res. 2015;116(5):846-856. doi:10.1161/CIRCRESAHA.116.305404.  [95] Morales GX, Bodiwala K, Elayi CS. Giant J-wave (Osborn wave) unrelated to hypothermia. Europace. 2011;13(2):283. doi:10.1093/europace/euq424.  [96] Crotti L, Odening KE, Sanguinetti MC. Heritable arrhythmias associated with abnormal function of cardiac potassium channels. Cardiovasc Res. 2020;116(9):1542-1556. doi:10.1093/cvr/cvaa068.  [97] Markham A, Brogden RN. Diltiazem. A review of its pharmacology and therapeutic use in older patients. Drugs Aging. 1993;3(4):363-390. doi:10.2165/00002512-199303040-00007.  [98] Shevelev VI, Kanorskiĭ SG. Klin Med (Mosk). 2012;90(9):59-63.  [99] Donnan GA. Therapy in cerebrovascular disease: current status and future directions. Med J Aust. 1991;155(8):563-571.  [100] Libbus I, Rosenbaum DS. Remodeling of cardiac repolarization: mechanisms and implications of memory. Card Electrophysiol Rev. 2002;6(3):302-310. doi:10.1023/a:1016349613464.  [101] Noll G, Kaufmann U, Wenzel RR, Lüscher TF. Therapeutische Massnahmen nach akutem Myokardinfarkt: differentieller Einsatz von PTCA, Chirurgie und Medikamenten [Therapeutic measures following acute myocardial infarct: differential use of PTCA, surgery and drugs]. Schweiz Med Wochenschr. 1996;126(5):164-176.  [102] Harada M, Tadevosyan A, Qi X, et al. Atrial Fibrillation Activates AMP-Dependent Protein Kinase and its Regulation of Cellular Calcium Handling: Potential Role in Metabolic Adaptation and Prevention of Progression. J Am Coll Cardiol. 2015;66(1):47-58. doi:10.1016/j.jacc.2015.04.056.  [103] Baczko I, Liknes D, Yang W, et al. Characterization of a novel multifunctional resveratrol derivative for the treatment of atrial fibrillation. Br J Pharmacol. 2014;171(1):92-106. doi:10.1111/bph.12409.  [104] Valdivia HH. Mechanisms of cardiac alternans in atrial cells: intracellular Ca2⁺ disturbances lead the way. Circ Res. 2015;116(5):778-780. doi:10.1161/CIRCRESAHA.115.305923.  [105] Sun H, Gaspo R, Leblanc N, Nattel S. Cellular mechanisms of atrial contractile dysfunction caused by sustained atrial tachycardia. Circulation. 1998;98(7):719-727. doi:10.1161/01.cir.98.7.719.  [106] Dong G, Liang F, Sun B, et al. Presence and function of stress granules in atrial fibrillation. PLoS One. 2019;14(4):e0213769. Published 2019 Apr 3. doi:10.1371/journal.pone.0213769.  [107] Francis J, Antzelevitch C. Atrial fibrillation and Brugada syndrome. J Am Coll Cardiol. 2008;51(12):1149-1153. doi:10.1016/j.jacc.2007.10.062.  [108] Christ T, Rozmaritsa N, Engel A, et al. Arrhythmias, elicited by catecholamines and serotonin, vanish in human chronic atrial fibrillation [published correction appears in Proc Natl Acad Sci U S A. 2014 Sep 23;111(38):14003]. Proc Natl Acad Sci U S A. 2014;111(30):11193-11198. doi:10.1073/pnas.1324132111.  [109] Merritt RE, Shrager JB. Prophylaxis and management of atrial fibrillation after general thoracic surgery. Thorac Surg Clin. 2012;22(1):13-v. doi:10.1016/j.thorsurg.2011.08.016.  [110] Kanjwal Y, Imran N, Grubb B. Deglutition induced atrial tachycardia and atrial fibrillation. Pacing Clin Electrophysiol. 2007;30(12):1575-1578. doi:10.1111/j.1540-8159.2007.00911.x.  [111] Bateman RM, Sharpe MD, Jagger JE, et al. 36th International Symposium on Intensive Care and Emergency Medicine : Brussels, Belgium. 15-18 March 2016 [published correction appears in Crit Care. 2016 Oct 24;20:347]. Crit Care. 2016;20(Suppl 2):94. Published 2016 Apr 20. doi:10.1186/s13054-016-1208-6.  [112] McDivitt JD, Barstow C. Cardiovascular Disease Update: Atrial Fibrillation. FP Essent. 2017;454:11-17.  [113] Tian G, Sun Y, Liu S, et al. Therapeutic Effects of Wenxin Keli in Cardiovascular Diseases: An Experimental and Mechanism Overview. Front Pharmacol. 2018;9:1005. Published 2018 Sep 5. doi:10.3389/fphar.2018.01005.  [114] Khan IA. Atrial stunning: determinants and cellular mechanisms. Am Heart J. 2003;145(5):787-794. doi:10.1016/S0002-8703(03)00086-3.  [115] Shin DG, Cho I, Hartaigh Bó, et al. Cardiovascular Events of Electrical Cardioversion Under Optimal Anticoagulation in Atrial Fibrillation: The Multicenter Analysis. Yonsei Med J. 2015;56(6):1552-1558. doi:10.3349/ymj.2015.56.6.1552.  [116] Khan IA. Atrial stunning: basics and clinical considerations. Int J Cardiol. 2003;92(2-3):113-128. doi:10.1016/s0167-5273(03)00107-4.  [117] Mayson SE, Greenspon AJ, Adams S, et al. The changing face of postoperative atrial fibrillation prevention: a review of current medical therapy. Cardiol Rev. 2007;15(5):231-241. doi:10.1097/CRD.0b013e31813e62bb.  [118] Bosch RF, Zeng X, Grammer JB, Popovic K, Mewis C, Kühlkamp V. Ionic mechanisms of electrical remodeling in human atrial fibrillation. Cardiovasc Res. 1999;44(1):121-131. doi:10.1016/s0008-6363(99)00178-9.  [119] Ho KM, Sheridan DJ, Paterson T. Use of intravenous magnesium to treat acute onset atrial fibrillation: a meta-analysis. Heart. 2007;93(11):1433-1440. doi:10.1136/hrt.2006.111492.  [120] Rücker-Martin C, Pecker F, Godreau D, Hatem SN. Dedifferentiation of atrial myocytes during atrial fibrillation: role of fibroblast proliferation in vitro. Cardiovasc Res. 2002;55(1):38-52. doi:10.1016/s0008-6363(02)00338-3.  [121] Van Wagoner DR, Nerbonne JM. Molecular basis of electrical remodeling in atrial fibrillation. J Mol Cell Cardiol. 2000;32(6):1101-1117. doi:10.1006/jmcc.2000.1147.  [122] Xi Y, Wu G, Ai T, et al. Ionic mechanisms underlying the effects of vasoactive intestinal polypeptide on canine atrial myocardium. Circ Arrhythm Electrophysiol. 2013;6(5):976-983. doi:10.1161/CIRCEP.113.000518.  [123] Gravez B, Tarjus A, Jaisser F. Mineralocorticoid receptor and cardiac arrhythmia. Clin Exp Pharmacol Physiol. 2013;40(12):910-915. doi:10.1111/1440-1681.12156.  [124] Abrol R, Page RL. Azimilide dihydrochloride: a new class III anti-arrhythmic agent. Expert Opin Investig Drugs. 2000;9(11):2705-2715. doi:10.1517/13543784.9.11.2705.  [125] Connell P, Word TA, Wehrens XHT. Targeting pathological leak of ryanodine receptors: preclinical progress and the potential impact on treatments for cardiac arrhythmias and heart failure. Expert Opin Ther Targets. 2020;24(1):25-36. doi:10.1080/14728222.2020.1708326.  [126] Borchard U, Hafner D. Ionenkanäle und Herzrhythmusstörungen [Ion channels and arrhythmias]. Z Kardiol. 2000;89 Suppl 3:6-12.  [127] Musco S, Conway EL, Kowey PR. Drug therapy for atrial fibrillation. Med Clin North Am. 2008;92(1):121-xi. doi:10.1016/j.mcna.2007.08.002.  [128] Podesser B, Schwarzacher S, Zwölfer W, et al. Combined perioperative infusion of nifedipine and metoprolol provides antiischemic and antiarrhythmic protection in patients undergoing elective aortocoronary bypass surgery. Thorac Cardiovasc Surg. 1993;41(3):173-179. doi:10.1055/s-2007-1013848.  [129] Zhou Y, Wu HJ, Zhang YH, Sun HY, Wong TM, Li GR. Ionic mechanisms underlying cardiac toxicity of the organochloride solvent trichloromethane. Toxicology. 2011;290(2-3):295-304. doi:10.1016/j.tox.2011.10.009.  [130] Li D, Zhang L, Kneller J, Nattel S. Potential ionic mechanism for repolarization differences between canine right and left atrium. Circ Res. 2001;88(11):1168-1175. doi:10.1161/hh1101.091266.  [131] Thodeti CK, Paruchuri S, Meszaros JG. A TRP to cardiac fibroblast differentiation. Channels (Austin). 2013;7(3):211-214. doi:10.4161/chan.24328.  [132] Kneller J, Sun H, Leblanc N, Nattel S. Remodeling of Ca(2+)-handling by atrial tachycardia: evidence for a role in loss of rate-adaptation. Cardiovasc Res. 2002;54(2):416-426. doi:10.1016/s0008-6363(02)00274-2.  [133] Zhang J, Tao R, Campbell KF, et al. Functional cardiac fibroblasts derived from human pluripotent stem cells via second heart field progenitors. Nat Commun. 2019;10(1):2238. Published 2019 May 20. doi:10.1038/s41467-019-09831-5.  [134] Sueda S, Kohno H, Oshita A, Izoe Y, Nomoto T, Fukuda H. Vasospastic heart failure: multiple spasm may cause transient heart failure?. J Cardiol. 2009;54(3):452-459. doi:10.1016/j.jjcc.2009.07.007.  [135] Vlase H, Lungu G, Vlase L. Cardiac disturbances in thyrotoxicosis: diagnosis, incidence, clinical features and management. Endocrinologie. 1991;29(3-4):155-160. |  |
| **27 were mathematical models:**  [1] Zile MA, Trayanova NA. Increased thin filament activation enhances alternans in human chronic atrial fibrillation. Am J Physiol Heart Circ Physiol. 2018;315(5):H1453-H1462. doi:10.1152/ajpheart.00658.2017  [2] Chang KC, Bayer JD, Trayanova NA. Disrupted calcium release as a mechanism for atrial alternans associated with human atrial fibrillation. PLoS Comput Biol. 2014;10(12):e1004011. Published 2014 Dec 11. doi:10.1371/journal.pcbi.1004011.  [3] Sutanto H, van Sloun B, Schönleitner P, van Zandvoort MAMJ, Antoons G, Heijman J. The Subcellular Distribution of Ryanodine Receptors and L-Type Ca2+ Channels Modulates Ca2+-Transient Properties and Spontaneous Ca2+-Release Events in Atrial Cardiomyocytes. Front Physiol. 2018;9:1108. Published 2018 Aug 14. doi:10.3389/fphys.2018.01108.  [4] Yoo S, Aistrup G, Shiferaw Y, et al. Oxidative stress creates a unique, CaMKII-mediated substrate for atrial fibrillation in heart failure. JCI Insight. 2018;3(21):e120728. Published 2018 Nov 2. doi:10.1172/jci.insight.120728.  [5] Koivumäki JT, Seemann G, Maleckar MM, Tavi P. In silico screening of the key cellular remodeling targets in chronic atrial fibrillation. PLoS Comput Biol. 2014;10(5):e1003620. Published 2014 May 22. doi:10.1371/journal.pcbi.1003620.  [6] Morotti S, McCulloch AD, Bers DM, Edwards AG, Grandi E. Atrial-selective targeting of arrhythmogenic phase-3 early afterdepolarizations in human myocytes. J Mol Cell Cardiol. 2016;96:63-71. doi:10.1016/j.yjmcc.2015.07.030.  [7] Tsai CT, Chiang FT, Tseng CD, et al. Increased expression of mineralocorticoid receptor in human atrial fibrillation and a cellular model of atrial fibrillation. J Am Coll Cardiol. 2010;55(8):758-770. doi:10.1016/j.jacc.2009.09.045.  [8] Lee YS, Hwang M, Song JS, et al. The Contribution of Ionic Currents to Rate-Dependent Action Potential Duration and Pattern of Reentry in a Mathematical Model of Human Atrial Fibrillation. PLoS One. 2016;11(3):e0150779. Published 2016 Mar 10. doi:10.1371/journal.pone.0150779.  [9] Severi S, Pogliani D, Fantini G, et al. Alterations of atrial electrophysiology induced by electrolyte variations: combined computational and P-wave analysis. Europace. 2010;12(6):842-849. doi:10.1093/europace/euq042  [10] Cherry EM, Evans SJ. Properties of two human atrial cell models in tissue: restitution, memory, propagation, and reentry. J Theor Biol. 2008;254(3):674-690. doi:10.1016/j.jtbi.2008.06.030.  [11] Grandi E, Pandit SV, Voigt N, et al. Human atrial action potential and Ca2+ model: sinus rhythm and chronic atrial fibrillation. Circ Res. 2011;109(9):1055-1066. doi:10.1161/CIRCRESAHA.111.253955.  [12] Adeniran I, MacIver DH, Garratt CJ, Ye J, Hancox JC, Zhang H. Effects of Persistent Atrial Fibrillation-Induced Electrical Remodeling on Atrial Electro-Mechanics - Insights from a 3D Model of the Human Atria. PLoS One. 2015;10(11):e0142397. Published 2015 Nov 25. doi:10.1371/journal.pone.0142397.  [13] Jeong DU, Lim KM. Influence of the KCNQ1 S140G Mutation on Human Ventricular Arrhythmogenesis and Pumping Performance: Simulation Study. Front Physiol. 2018;9:926. Published 2018 Jul 31. doi:10.3389/fphys.2018.00926.  [14] Courtemanche M, Ramirez RJ, Nattel S. Ionic mechanisms underlying human atrial action potential properties: insights from a mathematical model. Am J Physiol. 1998;275(1):H301-H321. doi:10.1152/ajpheart.1998.275.1.H301.  [15] Pandit SV, Berenfeld O, Anumonwo JM, et al. Ionic determinants of functional reentry in a 2-D model of human atrial cells during simulated chronic atrial fibrillation. Biophys J. 2005;88(6):3806-3821. doi:10.1529/biophysj.105.060459.  [16] Wolf RM, Glynn P, Hashemi S, et al. Atrial fibrillation and sinus node dysfunction in human ankyrin-B syndrome: a computational analysis. Am J Physiol Heart Circ Physiol. 2013;304(9):H1253-H1266. doi:10.1152/ajpheart.00734.2012.  [17] Grégoire-Lacoste F, Jacquemet V, Vinet A. Bifurcations, sustained oscillations and torus bursting involving ionic concentrations dynamics in a canine atrial cell model. Math Biosci. 2014;250:10-25. doi:10.1016/j.mbs.2014.01.010.  [18] Shy D, Gillet L, Abriel H. Cardiac sodium channel NaV1.5 distribution in myocytes via interacting proteins: the multiple pool model. Biochim Biophys Acta. 2013;1833(4):886-894. doi:10.1016/j.bbamcr.2012.10.026.  [19] Courtemanche M, Ramirez RJ, Nattel S. Ionic targets for drug therapy and atrial fibrillation-induced electrical remodeling: insights from a mathematical model. Cardiovasc Res. 1999;42(2):477-489. doi:10.1016/s0008-6363(99)00034-6.  [20] Redpath CJ, Bou Khalil M, Drozdzal G, Radisic M, McBride HM. Mitochondrial hyperfusion during oxidative stress is coupled to a dysregulation in calcium handling within a C2C12 cell model. PLoS One. 2013;8(7):e69165. Published 2013 Jul 8. doi:10.1371/journal.pone.0069165.  [21] Walmsley J, Rodriguez JF, Mirams GR, Burrage K, Efimov IR, Rodriguez B. mRNA expression levels in failing human hearts predict cellular electrophysiological remodeling: a population-based simulation study. PLoS One. 2013;8(2):e56359. doi:10.1371/journal.pone.0056359.  [22] Ashihara T, Namba T, Ito M, Kinoshita M, Nakazawa K. The dynamics of vortex-like reentry wave filaments in three-dimensional computer models. J Electrocardiol. 1999;32 Suppl:129-138. doi:10.1016/s0022-0736(99)90062-6.  [23] Kerckhoffs RC, Omens JH, McCulloch AD. Mechanical discoordination increases continuously after the onset of left bundle branch block despite constant electrical dyssynchrony in a computational model of cardiac electromechanics and growth. Europace. 2012;14 Suppl 5(Suppl 5):v65-v72. doi:10.1093/europace/eus274.  [24] Schild L, Bukowska A, Gardemann A, et al. Rapid pacing of embryoid bodies impairs mitochondrial ATP synthesis by a calcium-dependent mechanism--a model of in vitro differentiated cardiomyocytes to study molecular effects of tachycardia. Biochim Biophys Acta. 2006;1762(6):608-615. doi:10.1016/j.bbadis.2006.03.005.  [25] Adeniran I, MacIver DH, Garratt CJ, Ye J, Hancox JC, Zhang H. Effects of Persistent Atrial Fibrillation-Induced Electrical Remodeling on Atrial Electro-Mechanics - Insights from a 3D Model of the Human Atria. PLoS One. 2015;10(11):e0142397. Published 2015 Nov 25. doi:10.1371/journal.pone.0142397.  [26] Onal B, Gratz D, Hund TJ. Ca2+/calmodulin-dependent kinase II-dependent regulation of atrial myocyte late Na+ current, Ca2+ cycling, and excitability: a mathematical modeling study. Am J Physiol Heart Circ Physiol. 2017;313(6):H1227-H1239. doi:10.1152/ajpheart.00185.2017.  [27] Shuen-Hsin Liu, Ya-Wen Hsiao, Eric Chong, et. al. Rhodiola Inhibits Atrial Arrhythmogenesis in a Heart Failure Model. Journal of Cardiovascualr Electrophysiology. 2016; 27(9): 1093-1101. doi: 10.1111/jce.13026. |  |
| **23 were controlled trials or case reports:**  [1] Yan L, Jiang T, Yang X, Xu M. Spontaneous conversion of atrial fibrillation caused by severe hyperkalemia: A case report. Medicine (Baltimore). 2018;97(15):e0442. doi:10.1097/MD.0000000000010442.  [2] Hoshi Y, Nozawa Y, Ogasawara M, et al. Atrial electromechanical interval may predict cardioembolic stroke in apparently low risk elderly patients with paroxysmal atrial fibrillation. Echocardiography. 2014;31(2):140-148. doi:10.1111/echo.12329.  [3] Viland J, Langørgen J, Wendelbo Ø. A somnolent woman in her fifties with acute circulatory failure [published correction appears in Tidsskr Nor Laegeforen. 2019 Jun 20;139(10):]. En somnolent kvinne i 50-årene med akutt sirkulasjonssvikt [published correction appears in Tidsskr Nor Laegeforen. 2019 Jun 20;139(10):]. Tidsskr Nor Laegeforen. 2019;139(9):10.4045/tidsskr.18.0683. Published 2019 May 27. doi:10.4045/tidsskr.18.0683.  [4] De Vriese AS, Caluwé R, Pyfferoen L, et al. Multicenter Randomized Controlled Trial of Vitamin K Antagonist Replacement by Rivaroxaban with or without Vitamin K2 in Hemodialysis Patients with Atrial Fibrillation: the Valkyrie Study. J Am Soc Nephrol. 2020;31(1):186-196. doi:10.1681/ASN.2019060579.  [5] Cleland JG, Coletta AP, Abdellah AT, Cullington D, Clark AL, Rigby AS. Clinical trials update from the American Heart Association 2007: CORONA, RethinQ, MASCOT, AF-CHF, HART, MASTER, POISE and stem cell therapy. Eur J Heart Fail. 2008;10(1):102-108. doi:10.1016/j.ejheart.2007.12.004.  [6] Houltz B, Darpö B, Swedberg K, et al. Effects of the Ikr-blocker almokalant and predictors of conversion of chronic atrial tachyarrhythmias to sinus rhythm. A prospective study. Cardiovasc Drugs Ther. 1999;13(4):329-338. doi:10.1023/a:1007759500274.  [7] Heller DA, Ahern FM, Kozak M. Changes in rates of beta-blocker use between 1994 and 1997 among elderly survivors of acute myocardial infarction. Am Heart J. 2000;140(4):663-671. doi:10.1067/mhj.2000.109650.  [8] Kishima H, Mine T, Fukuhara E, Ashida K, Ishihara M. Predictors of left atrial appendage stunning after electrical cardioversion in patients with atrial fibrillation. Int J Cardiovasc Imaging. 2019;35(9):1549-1555. doi:10.1007/s10554-019-01592-y.  [9] Cho Y, Jeong W, Kim S, et al. Serial measurement of glyphosate blood concentration in a glyphosate potassium herbicide-intoxicated patient: A case report. Am J Emerg Med. 2019;37(8):1600.e5-1600.e6. doi:10.1016/j.ajem.2019.04.042.  [10] Suárez G, Herrera M, Vera A, Torrado E, Férriz J, Arboleda JA. Prediction on admission of in-hospital mortality in patients older than 70 years with acute myocardial infarction. Chest. 1995;108(1):83-88. doi:10.1378/chest.108.1.83.  [11] Proietti M, Raparelli V, Laroche C, et al. Adverse outcomes in patients with atrial fibrillation and peripheral arterial disease: a report from the EURObservational research programme pilot survey on atrial fibrillation. Europace. 2017;19(9):1439-1448. doi:10.1093/europace/euw169.  [12] Corley SD, Epstein AE, DiMarco JP, et al. Relationships between sinus rhythm, treatment, and survival in the Atrial Fibrillation Follow-Up Investigation of Rhythm Management (AFFIRM) Study. Circulation. 2004;109(12):1509-1513. doi:10.1161/01.CIR.0000121736.16643.11.  [13] Hinton W, McGovern A, Coyle R, et al. Incidence and prevalence of cardiovascular disease in English primary care: a cross-sectional and follow-up study of the Royal College of General Practitioners (RCGP) Research and Surveillance Centre (RSC). BMJ Open. 2018;8(8):e020282. Published 2018 Aug 20. doi:10.1136/bmjopen-2017-020282.  [14] Shuey MM, Gandelman JS, Chung CP, et al. Characteristics and treatment of African-American and European-American patients with resistant hypertension identified using the electronic health record in an academic health centre: a case-control study. BMJ Open. 2018;8(6):e021640. Published 2018 Jun 27. doi:10.1136/bmjopen-2018-021640.  [15] Meinertz T, Kirch W, Rosin L, et al. Management of atrial fibrillation by primary care physicians in Germany: baseline results of the ATRIUM registry. Clin Res Cardiol. 2011;100(10):897-905. doi:10.1007/s00392-011-0320-5.  [16] Fu S, Hirte H, Welch S, et al. First-in-human phase I study of SOR-C13, a TRPV6 calcium channel inhibitor, in patients with advanced solid tumors [published correction appears in Invest New Drugs. 2017 Jun;35(3):397]. Invest New Drugs. 2017;35(3):324-333. doi:10.1007/s10637-017-0438-z.  [17] Gibson AO, Blaha MJ, Arnan MK, et al. Coronary artery calcium and incident cerebrovascular events in an asymptomatic cohort. The MESA Study. JACC Cardiovasc Imaging. 2014;7(11):1108-1115. doi:10.1016/j.jcmg.2014.07.009.  [18] Hypothyroidism in adults. Levothyroxine if warranted by clinical and laboratory findings, not for simple TSH elevation. Prescrire Int. 2015;24(164):241-246.  [19] Umemoto S, Ogihara T, Rakugi H, et al. Effects of a benidipine-based combination therapy on the risk of stroke according to stroke subtype: the COPE trial. Hypertens Res. 2013;36(12):1088-1095. doi:10.1038/hr.2013.100.  [20] Toivonen L, Raatikainen P, Walfridsson H, et al. A randomized invasive cardiac electrophysiology study of the combined ion channel blocker AZD1305 in patients after catheter ablation of atrial flutter. J Cardiovasc Pharmacol. 2010;56(3):300-308. doi:10.1097/FJC.0b013e3181ed0859.  [21] Podesser BK, Schwarzacher S, Zwoelfer W, Binder TM, Wolner E, Seitelberger R. Comparison of perioperative myocardial protection with nifedipine versus nifedipine and metoprolol in patients undergoing elective coronary artery bypass grafting. J Thorac Cardiovasc Surg. 1995;110(5):1461-1469. doi:10.1016/S0022-5223(95)70069-2.  [22] Avezum A, Makdisse M, Spencer F, et al. Impact of age on management and outcome of acute coronary syndrome: observations from the Global Registry of Acute Coronary Events (GRACE). Am Heart J. 2005;149(1):67-73. doi:10.1016/j.ahj.2004.06.003.  [23] Yujing W, Congxin H, Shaning Y, et al. Digitalis does not improve left atrial mechanical dysfunction after successful electrical cardioversion of chronic atrial fibrillation. Cell Biochem Biophys. 2010;57(1):27-34. doi:10.1007/s12013-010-9080-5. |  |

**Supplementary** **Table S4.** Summary of the mechanisms and experimental models involved in the primary prevention of AF

| **Author** | **Species & Model** | **Drug applied** | **I_CaL_** | **I_NCX_** | **Western blotting/ Immunostaining** | **APD** | **Other observations** |
| --- | --- | --- | --- | --- | --- | --- | --- |
| Cheng (2020) | Mouse (HL-1 cells) | MIF* | ↔ | ↑ | ↑RyR2, NCX1, pRyR SERCA2a, pCAMKII. | n/a | ↑SR Ca^2+^ load and leak, I_to_, I_Kur_, |
|  |  | Anti-CD74** | ↔ | ↔ | n/a | n/a | ↓I_K_. |
| Chan (2019) | Rats  (RA)  Diabetic model | High-fat diet*,  High fructose/ cholesterol diet* | n/a | n/a | ↑collagen, TGF-β1, ox-CAMKIIδ, pPLN, PLN pRYR2, NCX1.  ↔SERCA2a, RyR2, CAMKIIδ. | ↔APD_20,_ APD_50_, APD_80_ and APD_90_ | ↑spontaneous AF, ↔CaTD. |
| Opel  (2015) | Mouse | Carbachol* | n/a | n/a | n/a | n/a | Bradycardic, ↓QT, ↑atrial ectopics. |
| Zhang (2015) | Rat  (LA)  HTN model | AoB | n/a | n/a | ↓Ca_v_1.2. ↔RyR2, pRyR, SERCA2a, PLN, pPLN, NCX1. | n/a | ↓ SR Ca^2+^ load.  ↔rate of Ca^2+^ extrusion. |
| Tazmini  (2020) | Rat | Hypokalaemia | n/a | ↓ | ↑NCX1. | ↑APD ↑EAD^68%^ ↑DAD | ↓I_NKA_ and I_K1_.  ↑I_Na_, SR Ca^2+^ content. |
|  |  | TTX** | n/a | n/a | n/a | ↓EAD | n/a |
|  |  | 4-AP* | n/a | n/a | n/a | ↑APD_50_ | n/a |
| Chiang  (2014) | Mouse | Spinophilin deficiency | n/a | n/a | ↔RyR2, pRyR, PP1, PP2A, pPLN, PKA, CAMKII, fibrosis.  ↑RyR P_o_ and open time, pCAMKII^43%^, pRyR.  ↓PP1:RyR2, RyR closing time. | n/a | ↑ectopic beats, inducibility^83%^. |
| Li  (2012) | WT Mouse | ↓FK-506 Binding Protein 12.6 | n/a | n/a | ↔CAMKII, pCAMKII, SERCA, NCX1. | ↔APD  ↑DADs^73%^ | ↑atrial flutter^53%^ |
| Mancarella (2008) | WT Mouse | Atria | n/a | n/a | ↑α1D Ca_v_1.2 | n/a | n/a |
|  |  | Ventricles | n/a | n/a | ↓ α1D Ca_v_1.2 | n/a | n/a |
| Pluteanu  (2015) | Rat  (LA) | SHR | ↓^20%^ | ↓ | ↑Fibrosis, CTGF.  ↓RyR2, Ca_v_1.2. ↑pRyR.  ↔SERCA2a, PLN, pPLN, CSQ, NCX1. | n/a | ↑BP, HR, SR Ca^2+^ load. |
| Herraiz-Martinez  (2015) | Human  (RA) | Ageing | ↓ | ↔ | ↓ Ca_v_1.2, SERCA2a, CSQ2 | n/a | ↓SR Ca^2+^ content |
| Chang  (2011) | Rabbit  (PV) | HF | ↓^56%^ | ↑^73%^ | n/a | ↑APD, ↑DAD^45%^  ↔ EAD | ↑I_ti_, I_Na-Late_. ↓I_Na_ |
| Huang  (2019) | Rabbit  (PV) | Arginine* | ↔ | ↑ | ↑NCX1, pCAMKII. ↔CAMKII. | n/a | ↑SR Ca^2+^ leak. |
| Kanaporis (2019) | Rabbit  (LA) | NS1643** | n/a | n/a | n/a | ↓APD | ↑I_K_. |
|  |  | ML277** | n/a | n/a | n/a | ↓APD | ↑I_K_. |
| Tsai  (2013) | Rabbit | Male | ↔ | ↔ | n/a | ↑ DAD ^57%^ | ↔I_Na_.  ↑I_Na-Late_, SR Ca^2+^ content. |
|  |  | Female | ↔ | ↔ | ↑cell width | ↔ APD | ↑cell capacitance |
|  |  | Ranolazine** | n/a | n/a | n/a | ↓DAD | n/a |
|  |  | Ouabain* | n/a | n/a | n/a | ↑DAD | n/a |
| Lin (2012) | Rabbit (LA)  Obese model | Adipocytes | ↑ | n/a | n/a | ↑APD_90_  ↔ DAD | ↑I_Na-Late_, I_to._ ↔ I_Na_, I_Kur._  ↓I_Kr_, I_K1_ |
| Lebek (2018) | Human (RA) | GS-680** | n/a | n/a | n/a | ↓EAD ↓DAD | ↓SR Ca^2+^ leak. |
| Greer (2020) | Human | AF | n/a | n/a | ↑CAMKII, ↑Na_v_1.5 | n/a | ↑I_Na-Late_. |
|  |  | Riluzole** | n/a | n/a | n/a | n/a | ↓atrial events |
| Lu (2020) | Rabbit (PV) | ANP** | ↓^41%^ | ↔ | n/a | ↓APD | ↓spontaneous activity,  ↓I_Na-Late_^60%^ |
| Chen (2015) | Rabbit (PV) | Indoxyl sulfate* | n/a | n/a | n/a | ↔APD  ↑DAD | ↔SR Ca^2+^ content.  ↑SR Ca^2+^ leak. |
|  | Rabbit (SAN) |  | n/a | n/a | n/a | ↔APD | n/a |
|  | Rabbit (LA) |  | n/a | n/a | n/a | ↓APD | n/a |
| Suenari (2011) | Rabbit (PV) | EPA** | n/a | n/a | n/a | ↓DAD, ↓APD | ↓SR Ca^2+^ content. |
| Chen (2008) | Rabbit (PV) | K201** | ↓^52%^ | ↓^67%^ | n/a | ↑APD^18%^ | n/a |
| Chang (2017) | Rabbit (LA) | EGCG** | ↓^37%^ | ↓ | n/a | ↓APD | ↓I_Na-Late_, SR Ca^2+53%^, I_to_. ↔I_Na_, I_Kur_. |
| Gassanov (2006) | Human (RA) | ANGII* | ↑ | n/a | ↔ Cx40, Cx43 | ↔APD | ↑SR Ca^2+^. ↓I_to_,  ↔ I_K1_, I_f_ , I_Kur_ |
|  |  | Candesartan** | ↓ | n/a | n/a | ↔APD | n/a |
| Gu (2013) | Mouse (HL-1 cells) | ANG II* | ↑ | ↑ | ↑ Ca_v_1.2, Kir2.1, Kir2.2  ↓K_v_4.2, K_v_1.5 | ↓APD | ↓I_to_, I_Kur_, ↑I_K1_ |
|  |  | Pioglitazone** | ↓ | n/a | ↑K_v_4.2, K_v_1.5  ↓Kir2.1, Kir2.2, Ca_v_1.2 | n/a | ↑I_to_, I_Kur_, ↓I_K1_ |
| Hove-Madsen (2006) | Human (RA) | CGS21680* | ↑ | ↑ | n/a | n/a | n/a |
|  |  | ZM241385** | ↓ | ↓ | n/a | n/a | Reverses CGS21680. |
|  |  | Tetracaine** | ↓ | ↓ | n/a | n/a | Reverses CGS21680. |
|  |  | H89** | ↓ | ↓ | n/a | n/a | Reverses CGS21680. |
| Huang (2016) | Male Rabbit (PV) | FGF23* | ↑ | ↑ | ↔ SERCA, PLN, CAMKII,  ↑pCAMKII | ↓APD  ↑DAD | ↑I_Na-Late_, ROS. |
|  |  | Ranolazine** | ↓ | ↓ | n/a | ↓APD | ↓HR, I_Na-Late_, ROS |
|  |  | KN93** |  |  | n/a | n/a | ↓I_Na-Late_ |
| Lu (2016) | Male Rabbit (PV) | Latrunculin-B** | ↓^55%^ | ↓ | n/a | n/a | ↑I_Kr_, ↓I_Na-Late_, I_to_, I_Kur_, ↔ I_Ks_ |
| Jiang (2019) | WT Mouse | Ibrutinib* | n/a | n/a | ↑RyR, CAMKII, PLN.  ↑Fibrosis^40%^, LA dilation | ↑DAD^50%^ | ↑ Ca^2+^ leak, ↓Ca^2+^ content |
| Lee (2007) | Male rabbit (PV) | TNFα* | ↓ | ↑ | ↔ SERCA | ↓APD | ↓SR Ca^2+^ content, ↔I_K1_. ↑Spontaneous activity, I_to_ |
| Lin (2013) | Male rabbit (LA)  Obesity model | Leptin* | ↓ | ↑^40%^ | n/a | ↓APD | ↑I_Na_, ↔ I_Na-Late_ |
| Lin (2014) | Mouse  (HL-1 cells)  Hyper-lipidemic model | Oleic Acid* | ↑ | ↓ | ↔ NCX, ↑SERCA, CAMII,  ↓NKA | ↓APD | ↑DADs, I_Na-Late_ |
|  |  | DIDS** | n/a | n/a | n/a | ↓DAD | n/a |
| Lkhagva (2014) | Rabbit (PV)  op model | MPT0E014** | ↔ | ↓ | ↓NCX, RyR,  ↔ Ca_v_1.2, SERCA, PLN, pPLN | ↔APD | ↑ERP, ↓AF inducibility.  ↔ HR, PR, QT |
| Lu (2016) | Mouse (HL-1 cells) | Colchicine** | ↓ | ↓ | ↓NCX^9%^, PLN^18%^, CAMKII^15%^, Kv1.4^10%^, Kv1.5^14%^, ↔ RyR | ↑APD | ↓I_to_, I_Ksus_,  ↔ I_Kur_, I_KAS_ |
| Lu (2018) | Male rabbit (PV) | ANG-(1-7)** | ↓ | ↓ | n/a | ↓DAD^28%^ | ↓ I_Na-Late_^38%^, SR Ca^2+^,  ↔ I_Na_ |
| Rios-Perez (2016) | Male Wistar rats | Aldosterone* | ↑^100%^ | n/a | ↔ Ca_v_1.2 | ↑APD | ↑ I_Na_, ↑I_K_^100%^ |
|  |  | DPTA-NONOate** | ↓ | n/a | n/a |  | n/a |
| Tsai (2011) | Rabbit (PV and LA) | Aliskiren* | ↓ | ↑ | n/a | ↔APD | ↓beat rate, ↔ ERP, ↑contractibility |
|  | Mice (HL-1 cells) | ANGII* | ↑ | ↑ | ↑ Ca_v_1.2, NCX1,  ↔ RyR, SERCA, PLN, Cx43, CSQ | ↓APD | ↑I_Kr_^100%^, ↓I_f_  ↔I_to_, I_K1_ |
|  |  | Verapamil** | ↓ | n/a | n/a | n/a | n/a |
|  |  | SEA0400** |  | ↓ | n/a | n/a | n/a |
| Wang (2019) | Aged Rabbit (LA) | Digoxin* | ↑ | n/a | n/a | ↑APD | ↑AF inducibility, HR,↓QT |
| Yang (2018) | Rabbit (LA)  Diabetic model | Alloxan* | ↑ | n/a | ↑ Ca_v_1.2, RyR, fibrosis. ↓SERCA, PLN. | n/a | ↑ERP, CV |
|  |  | Allopurinol** | ↓ | n/a | ↓ Ca_v_1.2, RyR, ↑SERCA, PLN, pPLN | n/a | n/a |
| Zuo (2018) | C57BL/6 mouse | TNFα* | ↑^67%^ | n/a | ↔ RyR, CAMKII | n/a | ↑ROS, ↓SR Ca^2+^. |
| Fischer (2015) | WT mouse | ATX-II* | ↑ | n/a | ↑ RyR, pPLN, pCAMKII | ↑APD^80%^ | ↑SR Ca^2+^, I_Na-Late_, I_Na_. |
|  |  | Ranolazine** |  | n/a | n/a | n/a | ↓I_Na_ |
| Musa (2013) | Sheep | PDGF-AB* | ↓ | ↔ | ↓ Ca_v_1.2, ↔ NCX, ↑fibrosis | ↓APD | n/a |
| Wongcharoen (2006) | Rabbit (PV) | KB-R7943** | ↓ | ↓ | n/a | n/a | ↓I_Na_, SR Ca^2+^, I_to_, I_K_, I_K1_ |
| Huang (2017) | Rabbit  (PV) | CKD | ↓ | ↑ | ↔ RyR2, SERCA, PLN.  ↑PKA, pPLN, pRyR. | n/a | ↑I_Na-Late_, ROS SR Ca^2+^, SERCA activity^48%^. |
| Yan (2018) | WT Mouse | MKK7D* | n/a | n/a | ↑pCAMKII | n/a | ↑AF inducibility |
|  |  | Aged | n/a | n/a | n/a | n/a | ↑AF inducibility, ↔ atrial fibrosis, ↑SR Ca^2+^ leak. |
|  |  | AC3-I** | n/a | n/a | n/a | n/a | ↔AF inducibility |
|  | HL-1 cell | Anisomycin*/ MKK7D* | n/a | n/a | n/a | n/a | ↑AF inducibility, ↑SR Ca^2+^ leak and content, ↑RyR P_o_, |
|  |  | KN93** | n/a | n/a | n/a | n/a | ↓SR Ca^2+^ leak |
|  |  | KN92** | n/a | n/a | n/a | n/a | ↔SR Ca^2+^ leak |
|  | Rabbit | Anisomycin* | n/a | n/a | ↑CAMKII, pJNK, pRyR, and pPLN-17, ↔NCX, SERCA, PKA, and pPLN-16 | n/a | ↑SR Ca^2+^ leak |
|  | Human (RA) | Aged | n/a | n/a | ↑CAMKII, pJNK | n/a | ↑AF inducibility |
| Tsai (2011) | Mouse  HL-cell | ANGII* | ↑ | n/a | ↑Ca_v_1.2 and NCX1, ↔Cx43, RyR2, CSQ, PLN, SERCA2a, and calstabin | ↓APD | ↑ heterogenous and non-propagated Ca^2+^ waves, ↑conduction block, ↑I_Kur_, ↔I_K1_ and I_to_, ↓I_f_, ↑triggered activity |
|  |  | Losartan** | n/a | n/a | ↓Ca_v_1.2 | n/a | ↓chaotic Ca^2+^ transients |
|  |  | Verapamil** | n/a | n/a | ↓Ca_v_1.2 | n/a | n/a |
|  |  | Chelerythrine** | n/a | n/a | ↓Ca_v_1.2 | n/a | n/a |
|  |  | SEA0400**  (NCX blocker) | n/a | n/a | n/a | n/a | ↓arrhythmogenicity |
| Shafaattalab (2019) | hESCs-derived | Atrial cells | n/a | n/a | n/a | ↓APD_80_^18%^, ↓APD_30_^50%^ | n/a |
|  |  | Ibrutinib* 0.1, 0.5, 1.0﻿uM | n/a | n/a | n/a | ↓APD_80_^8%, 26%, 31%^, ↑DADs | ↑CaTD, ↓cell viability, ↑alternan patterns |
|  |  | TKIs* | n/a | n/a | n/a | n/a | ↑CaTD _80_ |
| Tsao (2012) | Male Rabbit  (PV) | Aß_25-35_ | ↓^31%^ | ↓^33% & 37%^ | ↓NCX1^55%^, Ca_v_1.2^46%^, PLN^47%^, pPLN^72%^, pPLN/PLN^48%^, ↔SERCA2a, RyR2, Kir2.1, Kir2.3, K_v_1.5, K_v_4.2 | ↓APD_80_^13%^, ↔APD_20_ and APD_50_ | ↓beating rate, SR Ca^2+^ load^71%^, CaTA^28%^,  ↔I_to_ and I_Ksus_ |

*Pro-arrhythmic drug/agent. **Anti-arrhythmic drug/agent. For all protein expressions, ‘p’ stands for the phosphorylated state of a protein. n/a stands for not applicable. I_CaL_, L-type calcium current; I_CaT_, T-type calcium current; I_NCX_, sodium-calcium exchanger current; I_Na_, sodium current; I_Na-Late_, late sodium current; I_NKA_, sodium-potassium ATPase current; I_K_, potassium current; I_K1_, inward-rectifier potassium current; I_to_, transient-outward potassium current; I_Kur_, ultra-rapid delayed rectified potassium current; I_Kr_, rapid delayed rectifier potassium current; I_Ks_, slow delayed rectifying potassium current; I_Ksus_, sustained outward potassium current; I_f_, funny (pacemaker) current; APD, action potential duration; DAD, delayed after-depolarisation; EAD, early after-depolarisation; CV, conduction velocity; ERP, effective refractory period; BP, blood pressure; HR, heart rate; RyR2, ryanodine receptor 2; SR Ca^2+^, sarcoplasmic reticulum calcium; Ca_v_1.2, L-type calcium alpha 1c subunit; K_v_1.4, potassium voltage-gated transient outward channel; K_v_1.5, potassium voltage-gated ultra-rapid delayed rectified channel; K_v_2.1 and K_v_2.2, neuronal voltage-gated potassium channel; K_v_4.2, A-type potassium current; NCX1, cardiac sodium-calcium exchanger 1; PLN, phospholamban; SERCA, sarco/endoplasmic reticulum Ca^2+^ ATPase 2a; CAMKII, calcium/calmodulin-dependent protein kinase I; ox-CAMKIIδ, oxidative-CAMKII; PKA, protein kinase A; Cx, connexion; CaM, camodulin; CSQ, calsequestrin; TGF, transforming growth factor; MIF, macrophage migration inhibitory factor; CD74, cluster of differentiation 74 gene; GIRK, G-protein-gated inwardly rectifying potassium channel; P_o_, open probability; PP, protein phosphatase; CTGF, connective tissue growth factor; ROS, redox oxidative species; LA, left atria; RA, right atria; PV, pulmonary vein; SAN, sinoatrial node; AF, atrial fibrillation; HF, heart failure; AoB, ascending aortic banding; HTN, hypertension; CKD, chronic kidney disease; SHR, spontaneously hypertensive rat; WT, wild-type; ANP, atrial natriuretic peptide; TTX, tetrodotoxin; 4-AP, 4-aminopyridine; EPA, eicosapentaenoic acid; EGCG, epigallocatechin-3-gallate; ANG, angiotensin; FGF23, fibroblast growth factor 23; DIDS, 4,4′-Diisothiocyanatostilbene-2,2′-disulfonic acid; NO, nitric oxide; DPTA NONOate, dipropylenetriamine NONOate; TNFα, tumour necrosis factor alpha; PDGF-AB, platelet-derived growth factor antibody; KB-R7943, N-methyl-D-aspartate receptor blocker; NS1643, rapid delayed rectifier channel agonist; ML277, slow delayed rectifier channel agonist; SEA0400, sodium-calcium exchanger blocker; GS-680, CAMKII inhibitor; K201, RyR stabiliser; CGS21680, 2-p-(2-Carboxyethyl)phenethylamino-5′-N-ethylcarboxamidoadenosine hydrochloride hydrate; ZM241385, adenosine 2A receptor antagonist; MPT0E014, histone deacetylase inhibitor; H89, protein kinase A inhibitor; KN93, selective CAMKII blocker; CaTD, calcium transient duration; JNK, c-Jun N-terminal kinase; MKK7D, mitogen-activated protein kinase 7 (cardiac specific upstream activator of JNK),AC3-I, autocamtide-3 derived inhibitory peptide (highly specific inhibitor of calmodulin-dependent protein kinase II); KN92, inactive analog of KN93; hESCs, human embryonic stem cell; uM, micromolar; TKIs, tyrosine kinase inhibitiors; Aß_25-35_, beta-amyloid fragment 25-35; Kir2.1 and Kir2.3, inward rectifier potassium current subunit; Na_v_1.5, integral membrane protein and tetrodotoxin-resistant voltage-gated sodium channel subunit.

**Supplementary** **Table S5.** Summary of the mechanisms and experimental models involved in the secondary prevention of AF

| **Author** | **Species & Model** | **Drug applied** | **I_CaL_** | **I_NCX_** | **Western blotting/ Immunostaining** | **APD** | **Other observations** |
| --- | --- | --- | --- | --- | --- | --- | --- |
| Llach (2011) | Human (RA) | AF | ↓ | ↑ | ↑pRyR | n/a | n/a |
|  |  | CGS21680* | ↔ | ↑ | ↑pRyR | n/a | ↔SR Ca^2+^ load |
|  |  | ZM241385** | n/a | ↓ | n/a | n/a | n/a |
|  |  | ADA** | n/a | ↓ | n/a | n/a | n/a |
| Macquaide (2015) | Sheep | Persistent AF | n/a | n/a | ↔RyR cluster size.  ↓RyR centroid distance^23%^. ↑total number: RyR and CRUs. | n/a | n/a |
| Pasqualin  (2018) | Rat  (PV/ LA) | PVCM | n/a | n/a | ↑density and tubular network. | n/a | ↔SR Ca^2+^ load. |
|  |  | LACM | n/a | n/a | ↓tubular network. | n/a |  |
| Lenaerts (2009) | Sheep | Persistent AF | ↓ | ↑ | ↓ Ca_v_1.2^68%^, ↓tubule density^55.5%^,  ↔SERCA, PLN, pPLN, RyR2. Cell hypertrophy. | n/a | ↔SR Ca^2+^ content. |
| Hove-Madsen  (2004) | Human  (RA) | AF | n/a | ↑ | n/a | n/a | ↔SR Ca^2+^ content. |
| Zhang  (2013) | Human | Atrial myocytes | n/a | n/a | ↑TRP1, STIM1, Orai1. ↓TRP3. | n/a | n/a |
|  |  | ANGII* or ET-1*. | n/a | n/a | n/a | n/a | ↑I_TRP1_ |
|  |  | La^3+^ ** | n/a | n/a | n/a | n/a | ↓I_TRP1_ |
| Zhou  (2018) | Dog | Fontan operation (atrial tachyarrhythmia) | ↓ | n/a | ↓SERCA, Ca_v_1.2.  ↑NCX1, CAMKII, pRyR2. | n/a | ↑SR Ca^2+^ content, inducibility^85.71%^, |
| Suenari  (2011) | Rabbit  (LA) | Anterior LA | ↔ | n/a | ↔cell morphology. | ↔APD_90_ | ↔I_to_, I_Kur_. |
|  |  | Posterior LA | ↔ | ↓ | ↓NCX1. ↑RyR.  ↓wall thickness. | ↑DAD^53%^ | ↑I_Na_, I_Na-late_, SR Ca^2+^ content. ↓I_K1_. |
| Coutu  (2006) | Dog  (PV/ LA) | PV | ↓ | n/a | n/a | n/a | ↔SR Ca^2+^. |
| Workman  (2001) | Human  (RA) | Chronic AF | ↓^63%^ | n/a | n/a | ↓APD^39%^ | ↓ERP^49%^.↓I_to_^65%^. ↑I_K1_. ↔ I_Ksus_. |
|  |  | Nifedipine** | ↓ | n/a | n/a | ↓APD | ↔ ERP. |
|  |  | 4-AP* |  | n/a | n/a | ↑APD^177%^ | ↓I_to_ and I_Ksus_. ↑ERP^33%^. |
| Lenaerts  (2011) | Sheep  tachypaced | Molsidomine** | ↑^84.8%^ | n/a |  | ↑APD | ↔PR, CO. ↓BP, ERP. |
|  |  | L-NAME* | n/a | n/a | n/a | n/a | ↑BP, PR, indicibility^33.3%^.  ↓CO, ERP. |
|  |  | Sodium nitroprusside** | ↑^64.1%^ | n/a | n/a | n/a | n/a |
| Voigt  (2014) | Human  (RA) | Paroxysmal AF | ↔ | ↔ | ↔NCX1, pCAMKII, CaM, PKA, PP1, PP2A, pRyR, pRyR. ↑RyR P_o_, pPKA, RyR. ↓SERCA. | ↔APD  ↑DAD | ↑SR Ca^2+^ content and load. |
| Hartmann  (2016) | Human  (RA) | Ranolazine** | n/a | n/a | n/a | ↑APD_90_ | ↓SR Ca^2+^ leak. |
|  |  | Ranolazine + Dronedarone** | n/a | n/a | n/a | ↑APD_90_ | n/a |
| Greiser (2014) | Rabbit RAP model | Nitrendipine** | ↓ | ↓ | ↔ NCX, pPLN, CAMKII, pCAMKII, ↓SERCA^30%^, ↑CaM | ↓APD | ↓ERP |
|  | Human (RA) Persistent AF |  |  |  |  |  |  |
| Neef (2010) | Human (RA) AF | KN93** | ↔ | ↓ | ↑pCAMKII^110%^, NCX^43%^, CAMKII^40%^, hypertrophy  ↓ RyR2^445%^, ↔ PLN, pPLN | ↓APD | ↑SR Ca^2+^ leak |
|  |  | KN92** | ↓ | n/a | n/a | n/a | ↑SR Ca^2+^ leak |
| Voigt (2012) | Human (RA) | Chronic AF | ↓^42%^ | ↑ | ↑^60%^CAMKII, ↑^40%^pCAMKII, pRyR2, RyR P_o_, ↔ RyR2 | ↓APD  ↑DAD | ↑Spontaneous activity, SR Ca^2+^ leak |
| Qi (2008) | Canine | Tachypacing | ↓ | n/a | ↓ Ca_v_1.2. ↔ cell morphology | n/a | n/a |
|  |  | Nimodipine** | ↑ | n/a | n/a | ↓APD | ↓I_to_, ↔ I_CaT_, I_Kr_, I_Ks_, ERP |
|  |  | BAPTA-AM** | ↑ | n/a | n/a | ↓APD | n/a |
|  |  | W-7** | ↑ | n/a | n/a | ↔APD | n/a |
|  |  | FK-506** | ↑ | n/a | ↑Ca_v_1.2 | ↓APD | n/a |
|  |  | INCA-6** | ↑ | n/a | ↑ Ca_v_1.2 | n/a | n/a |
| Avula (2018) | Sheep LAMI | Dantrolene** | n/a | n/a | ↑RyR2 binding and CaM. | n/a | ↔SR Ca^2+^. |
| Voigt (2012) | Human | Chronic AF | n/a | n/a | n/a | n/a | ↑SR Ca^2+^ leak |
|  |  | KN93** | n/a | n/a | ↓ RyR2, RyR P_o_ | n/a | ↓SR Ca^2+^ |
|  |  | KN92** | n/a | n/a | ↔ RyR P_o_ | n/a | ↑ SR Ca^2+^ leak |
|  |  | H89** | n/a | n/a | ↔ RyR P_o_ | n/a | ↑ SR Ca^2+^ leak |
| Wakili (2010) | Canine  (RA) | Atrial tachycardia remodelling | ↓^63%^ | ↑ | ↔RyR2, pRyR, pRyR, CSQ, SERCA, NCX, PLN, pPKA, pCAMKII. ↓tubular density^60%^. | n/a | ↓cell shortening^85%^, relaxation^81%^. |
| Hartmann (2017) | Human  (LA) | Persistent AF | n/a | n/a | n/a | ↑DADs, ↔APD_50_ and APD_90_. | n/a |
|  |  | Dantrolene** | n/a | n/a | n/a | ↓DADs | ↓total SR Ca^2+^ leak^56%^ |
| Kanaporis (2017) | AP_CaT_Small_ | n/a | n/a | n/a | n/a | n/a | ↑Ca^2+^ wave alternans ratio, ↑SR Ca^2+^ load. |
|  | AP_CaT_Large_ | n/a | ↑ | n/a | n/a |  | ↑Ca^2+^ release magnitude^17%^,  ↓t_80%_peak_. |
| Liang (2008) | Human (RA) | Persistent AF | n/a | n/a | n/a | n/a | ↔ SR Ca^2+^ load |
|  |  | Tetracaine**/ Ryanodine* | n/a | n/a | n/a | n/a | ↓local Ca^2+^ elevations |
| Henry (2018) | Rat  Spague-Dawley  (PV/ LA) | EFS at 1Hz, 3Hz, 5Hz, 9Hz | n/a | n/a | n/a | n/a | ↑length and width of PVCM than LACM,  ↑wave velocity at 3Hz^38%^ and 5Hz^65%^. |
| Jones (2008) | White Rabbits  (SA/ PV/ LA) | SA node  (EFS at 3Hz) | n/a | n/a | ↓RyR2 and NCX1, ↔SERCA2a | n/a | ↑cell structural heterogeneity, ↑narrow, ↓cell capacitance and cell volume, ↑spontaneous activity and diastolic Ca^2+^,  ↓peak systolic Ca^2+^ and SR Ca^2+^ content,  ↔Ca^2+^ transient rise time and CaTD. |
|  |  | Ryanodine* | n/a | n/a | n/a | n/a | ↓Ca transient decay, ↑diastolic Ca^2+^, ↑CaTD^139-164%^ |
|  |  | Nickel** (NCX blocker) | n/a | n/a | n/a | n/a | ↓Ca^2+^ transient decay rate |
| Logantha (2010) | Rat  Spague-Dawley  (PV) | EFS at 1.1-2.4Hz | n/a | n/a | n/a | n/a | ↑spontaneous, asynchronous and obliterated Ca^2+^ waves |
|  |  | Ryanodine* | n/a | n/a | n/a | n/a | ↓spontaneous Ca^2+^ waves^67%^. |
|  |  | Caffeine* | n/a | n/a | n/a | n/a | ↓spontaneous Ca^2+^ waves^86%^. |
|  |  | 2-APB**  (IP_3_R blocker) | n/a | n/a | n/a | n/a | ↓spontaneous Ca^2+^ waves^47%^. |
|  |  | CPA**  (SERCA inhibitor) | n/a | n/a | n/a | n/a | ↓spontaneous Ca^2+^ waves^43%^. |
| Zhang (2010) | WT Mouse | ﻿EFS at 1Hz | n/a | n/a | n/a | n/a | ↓subsidary peaks and SR Ca^2+^ load |
|  |  | FPL-64176* | n/a | n/a | n/a | n/a | ↑persistent and irregular Ca^2+^ transient pattern, ↑arrhythmic effects with AV block, ↔AERP |
|  |  | Nifedipine** | n/a | n/a | n/a | n/a | ↑irregular activity and diastolic Ca^2+^ waves |
|  |  | CPA** | n/a | n/a | n/a | n/a | ↑diastolic Ca^2+^ waves and irregularity |
| Voigt (2013) | Human | Chronic AF | ↓ | n/a | n/a | n/a | ↓cell viability and [Ca^2+^]_i_ |
| Uma (2018) | Sheep  3-4months | LAMI | n/a | n/a | n/a | n/a | ↔MAP, ischaemia, ↑AF episodes |
|  |  | IZ | n/a | n/a | ↓NOS-1, ↑CaM,  ↑NAPDH and XO activity,  ↑[^3^H] ryanodine binding to RyR | n/a | ↓cardiac contractibility, ↑interstitial fibrosis, ROS and conduction abnormalities, ↔SR Ca^2+^ load |
|  |  | Dantrolene** | n/a | n/a | ↓ [^3^H] ryanodine binding to RyR | n/a | ↔interstitial fibrosis and HR changes, ↓AF episodes and SFDs |
| Wakili (2010) | Adult Dog | ATR | ↓ | n/a | ↔RyR2, pRyR, CSQ, NCX1, SERCA2a, PLN, pPLN | n/a | ↓AERP, cell shortening, SR Ca^2+^ content^30%^,  ↔RA pressure, diastolic [Ca^2+^]_i_, ↑contractile dysfunction, Ca^2+^ transient decay rate |

*Pro-arrhythmic drug/agent. **Anti-arrhythmic drug/agent. For all protein expressions, ‘p’ stands for the phosphorylated state of a protein. n/a stands for not applicable. I_CaL_, L-type calcium current; I_Na_, sodium current; I_Na-Late_, late sodium current; I_K1_, inward-rectifier potassium current; I_to_, transient-outward potassium current; I_Kur_, ultra-rapid delayed rectified potassium current; I_Kr_, rapid delayed rectifier potassium current; I_Ks_, slow delayed rectifying potassium current; I_Ksus_, sustained outward potassium current; I_TRP1_, transient receptor potential 1 current; APD, action potential duration; DAD, delayed after-depolarisation; CO, cardiac output; ERP, effective refractory period; BP, blood pressure; HR, heart rate; RyR2, ryanodine receptor 2; SR Ca^2+^, sarcoplasmic reticulum calcium; Ca_v_1.2, L-type calcium alpha 1c subunit; NCX1, cardiac sodium-calcium exchanger 1; PLN, phospholamban; SERCA, sarco/endoplasmic reticulum Ca^2+^ ATPase 2a; CAMKII, calcium/calmodulin-dependent protein kinase I; CRUs, calcium-release units; PKA, protein kinase A; Cx, connexion; CaM, camodulin; CSQ, calsequestrin; ADA, adenosine deaminase; TRP, transient receptor potential channels; P_o_, open probability; STIM1, stromal interaction molecule 1; Orai1, calcium release-activated calcium modulator 1; PP, protein phosphatase; LA, left atria; RA, right atria; PV, pulmonary vein; AF, atrial fibrillation; RAP, rapid-atrial pacing; 4-AP, 4-aminopyridine; La^3+^, lanthanum; Molsidomine, N-ethoxycarbonyl-3-morpholino-sydnonimine; L-NAME, N^w^-nitro-L-arginine methylester; ANG, angiotensin; ET-1, endothelin-1; BAPTA-AM, N,N'-[1,2-ethanediylbis(oxy-2,1-phenylene)]*bis*[N-[2-[(acetyloxy)methoxy]-2-oxoethyl]-1,1'-*bis*[(acetyloxy)methyl] ester-glycine; INCA-6, nuclear factor of activated T-cells inhibitor; FK-506, calcineurin inhibitor; W-7, calcium-camodulin inhibitor; CGS21680, 2-p-(2-Carboxyethyl)phenethylamino-5′-N-ethylcarboxamidoadenosine hydrochloride hydrate; ZM241385, adenosine 2A receptor antagonist; H89, protein kinase A inhibitor; KN93, selective CAMKII blocker; KN92, inactive analog of KN93; AP_CaT_Small_, voltage command to record action potentials during small amplitude alternans calcium transient; AP_CaT_Large_, voltage command to record action potentials during large amplitude alternans calcium transient; t_80%_peak_, time to 80% of the calcium transient peak; FWHM, full width at half maximum intensity; FDHM, full duration at half maximum intensity; CM, cardiomyocytes; EFS, electrically field stimulation; CaTD, calcium transient duration; CaTA, calcium transient amplitude; CaSpF, calcium spark frequency; KCl, potassium chloride; 2-APB, 2-aminoethoxyl diphenylborinate; IP_3_, inositol triphosphate; CPA, cyproterone acetate; ﻿FPL-64176, novel nondihydropyridine Ca^2+^ channel activator; AERP, atrial effective refractory period; [Ca^2+^]_i_, intracellular calcium concentration; LAMI, left atrial myocardial infarction; IZ, ischaemic zone; HR, heart rate; MAP, mean aterial pressure; SFDs, spontaneous focal discharges; ROS, reactive oxygen species; NOS-1, nitric oxide synthase-1; NAPDH, nicotinamide adenine dinucleotide phosphate; XO, xanthine oxidase; [^3^H], ryanodine binding assay; ATR, atrial tachycardia remodeling.

**Supplementary Figures**

**Supplementary Figure S1.** Calcium spark frequency in the primary **(A)** diseased and **(B)** proarrhythmic subgroups, and the secondary **(C)** paroxysmal, and **(D)** permanent atrial fibrillation subgroups. ATXII, anemonia viridis toxin 2; CGS21680, adenosine 2A agonist; ANGII, angiotensin II; TNFa, tumour necrosis factor alpha; KN92, inactive analog of KN93; KN93, calcium/calmodulin-dependent protein kinase II inhibitor.

**Supplementary Figure S2.** Calcium transient amplitude (CaTA) in the primary prevention group in the presence of **(A)** proarrhythmic drug only, and a combined result of both **(B)** diseased and proarrhythmic subgroup. CaTA plots were shown in the secondary **(C)** paroxysmal, **(D)** permanent and **(E)** both paroxysmal and permanent atrial fibrillation subgroups. ANGII, angiotensin II; CGS21680, adenosine 2A agonist; FGF23, fibroblast growth factor 23; ATXII, anemonia viridis toxin 2; indoxyl, indoxyl sulphate; SIN-1, 3-Morpholino-Sydnonimine; TNFa, tumour necrosis factor alpha; Nitr, nitrendipine; KN92, Inactive analog of KN93; KN93, calcium/calmodulin-dependent protein kinase II inhibitor; ZM241385, adenosine 2A antagonists; ADO, adenosine.

**Supplementary Figure S3.** Calcium transient frequency in the primary **(A**) proarrhythmic and **(B)** antiarrhythmic subgroups. CGS21680, adenosine 2A agonist; ZM241385, adenosine 2A antagonists; ADO, adenosine.

**Supplementary Figure S4.** Calcium spark amplitude (CaSpA) in the primary **(A)** diseased, **(B)** proarrhythmic, **(C)** both diseased and proarrhythmic, and **(D)** antiarrhythmic drug subgroups, and secondary **(E)** paroxysmal, **(F)** permanent and **(G)** paroxysmal and permanent atrial fibrillation subgroups combined. TNFa, tumour necrosis factor alpha; ANGII, angiotensin II; ATXII, anemonia viridis toxin 2; CGS21680, adenosine 2A agonist; DTT, dithiothreitol; Can, candesartan; Ran, ranolazine; AIP, autocamide-2-related inhibitory peptide; TTX, tetrodotoxin; KBR7943, reverse-mode sodium/calcium exchanger inhibitor; H89, protein kinase inhibitor; GS680, calcium/calmodulin-dependent protein kinase II inhibitor.

**Supplementary Figure S5:** Sarcoplasmic reticulum (SR) calcium (Ca^2+^) load and leak. SR Ca^2+^ load in the primary **(A)** diseased, **(B)** proarrhythmic, and **(C)** both diseased and proarrhythmic drug subgroups, and secondary **(D)** paroxysmal, **(E)** permanent and **(F)** paroxysmal and permanent atrial fibrillation (AF) studies combined, and Ca^2+^ leak in secondary **(G)** paroxysmal, **(H)** permanent and **(I)** paroxysmal and permanent AF studies combined. TNFa, tumour necrosis factor alpha; ANGII, angiotensin II; ATXII, anemonia viridis toxin 2; FGF23, fibroblast growth factor 23; MIF, macrophage inhibitory factor; indoxyl, indoxyl sulphate; CGS21680, adenosine 2A agonist; AVP, arginine vasopressin; Ran, ranolazine; H89, protein kinase inhibitor; Dron, dronedarone; KN92, Inactive analog of KN93; KN93, calcium/calmodulin-dependent protein kinase II inhibitor.

**Supplementary Figure S6.** L-type calcium current (I_CaL_) and protein expression (Ca_v_1.2). I_CaL_ in the primary **(A)** proarrhythmic, **(B)** diseased and proarrhythmic subgroup and secondary **(C)** paroxysmal and **(D)** permanent atrial fibrillation (AF) subgroups, and its protein expression (Ca_v_1.2) in **(E)** primary diseased and **(F)** secondary permanent AF group. ANGII, angiotensin II; CGS21680, adenosine 2A agonist; TNFa, tumour necrosis factor alpha; Aldo, aldosterone; DPTA, nitric oxide donor; IBMX, 3-isobutyl-1-methylxanthine; AVP, arginine vasopressin.

**Supplementary Figure S7.** Sodium-calcium exchanger pump current density (I_NCX_) and protein expression (NCX1). I_NCX_ in the primary **(A)** diseased, and **(B)** proarrhythmic subgroups, and its protein expression (NCX1) in the primary **(C)** diseased, **(D)** proarrhythmic, and a **(E)** combination of diseased and proarrhythmic subgroups, and secondary **(F)** paroxysmal, **(G)** permanent, and **(H)** both paroxysmal and permanent atrial fibrillation subgroups. ANGII, angiotensin II; CGS21680, adenosine 2A agonist; TNFa, tumour necrosis factor alpha; MIF, macrophage inhibitory factor; AVP, arginine vasopressin.
